# Detecting mild traumatic brain injury with MEG scan data: One-vs-K-sample tests

**DOI:** 10.1162/IMAG.a.137

**Published:** 2025-09-08

**Authors:** Jian Zhang, Gary Green

**Affiliations:** School of Mathematics, Statistics and Actuarial Science, University of Kent, Canterbury, Kent, United Kingdom; Department of Psychology, University of York, York, United Kingdom; Innovision IP Ltd., London, United Kingdom

**Keywords:** MEG spectrum data, normal mixtures, likelihood ratio test in frequency domain, Anderson–Darling test and subject-heterogeneity

## Abstract

Magnetoencephalography (MEG) scanner has been shown to be more accurate than other medical devices in detecting mild traumatic brain injury (mTBI). However, MEG scan data in certain spectrum ranges can be skewed, multimodal, and heterogeneous which can mislead the conventional case–control analysis that requires the data to be homogeneous and normally distributed within the control group. To meet this challenge, we propose a flexible one-vs-K-sample testing procedure for detecting brain injury for a single-case versus heterogeneous controls. The new procedure begins with source magnitude imaging using MEG scan data in frequency domain, followed by region-wise contrast tests for abnormality between the case and controls. The critical values for these tests are automatically determined by cross-validation. We adjust the testing results for heterogeneity effects by similarity analysis. An asymptotic theory is established for the proposed test statistic. By simulated and real data analyses in the context of neurotrauma, we show that the proposed test outperforms commonly used nonparametric methods in terms of overall accuracy and ability in accommodating data non-normality and subject-heterogeneity.

## 1 Introduction

Around 8 to 12% of the global population have been estimated to live with traumatic brain injury (TBI) (Frost et al., 2013; James et al., 2019). Although mild TBIs, which include concussions, account for 70–90% of TBI cases, there is no generally accepted standard for diagnosing one. The early identification of mTBI and accurate assessment of recovery after a treatment are vital to ensuring the best treatment and rehabilitation outcomes. The state of the art in finding a neural signature of mTBI and classifying patterns of neural damages that determine behavioural recovery from early post-injury to sub-acute outcome is at an early stage of investigation. As clinical assessment tools, such as the Glasgow Coma Scale, which scores a person’s verbal and motor responses, as well as eye opening, are subjective, clinicians often turn to imaging techniques. The scanners currently used to diagnose these injuries, structural magnetic resonance imaging (sMRI) and computerised tomography (CT), have a less than 10% detection accuracy and are not sensitive enough to identify the microscopic damage that is characteristic of mTBIs. In contrast, MEG scanner can detect subtle pathology that often goes undetected in individuals with mTBI when using sMRI and CT (Huang et al., 2014). Allen et al. (2021) and Huang et al. (2020) provided a recent review of use of MEG to assess a brain injury. However, MEG scan data in certain spectrum ranges can be skewed, multimodal, and heterogeneous which raises a critical issue to the conventional case–control analysis that requires the data to be homogeneous and normally distributed. In this paper, we address the above issue through MEG-based one-vs-K-sample (OK) hypothesis tests for a single case compared with a group of healthy controls.

MEG is a non-invasive functional brain-mapping device that detects magnetic fields induced by neuronal electrical activity, with millisecond time scale resolution (Schwartz et al., 2010). Statistical modelling of MEG scan data can be found, for example, in Zhang et al. (2014). The MEG scans for brain injury are obtained when a subject is in a resting state with eyes open and eyes closed, and ideally repeated twice with sample rate 1 kHz for 8 minutes. Prior to acquisition, empty room data are acquired so that correct noise removal procedures can be exploited. The voxel-wise MEG source magnitude images were obtained using a high-resolution inverse imaging method called Fast-VESTAL (Huang et al., 2012). Brain activity is often described in terms of the amount of oscillatory activity in different frequency bands, for example, the delta band describes slow waves with frequencies between 1 and 4 Hz while the gamma band is for the spectrum ranging from 30 to 80 Hz. The bands with other ranges include theta (5–7 Hz), alpha (8–12 Hz), and beta (15–29 Hz). Huang et al. (2012) measured functional changes using MEG in both civilian and military personnel with mTBI showing an increase in delta power after head injury at both the group and individual levels. These changes in low frequency, considered pathologic in otherwise healthy adults, have been associated with brain lesions, Parkinson’s disease, hypoxia, schizophrenia, and states associated with abnormal or damaged brain tissue, in addition to mTBI (Knyazev, 2012). These facts suggest MEG measured delta wave power is a compelling diagnostic and prognostic tool for concussion in human brain (Davenport et al., 2022). Although non-invasive detection of gamma-band activity is challenging since coherently active source areas are small at such frequencies, Huang et al. (2020) revealed abnormal resting-state gamma activity in mTBI by using MEG scans. All these indicated that brain oscillatory wave at different frequency bands could provide promising features for differentiating mTBI patients from controls. The overall and group effects of these features have also been addressed by the utilisation of advanced machine learning and deep learning techniques in both source and sensor levels (Aaltonen et al., 2023; Huang et al., 2021 and references therein), based on the assumption of the existence of training and testing mTBI data which are often unavailable in single-subject studies.

In the context of multiple-sample studies, statistical tests such as two-sample t-test, Crawford–Garthwaite p-value test, two-sample or K-sample Anderson–Darling (AD) test and Disco analysis can be employed to infer the presence of cognitive impairments in a patient (Crawford & Garthwaite, 2007; Huang et al., 2016; Rizzo & Székely, 2010; Scholz & Stephens, 1987). The Crawford–Garthwaite test is a Bayesian t-test for a mean shift in a case compared with controls under a normality assumption. The two-sample and K-sample AD tests involve determining whether multiple samples are each drawn from the same distribution. Disco analysis extended the classical multivariate analysis of variance (MANOVA) with multiple samples. Like many non-parametric tests although the AD and Disco have not made strong distributional assumptions, tests based on specific distributional assumptions are generally believed to be more powerful than non-parametric techniques if the distributional assumptions can be validated. Researchers have witnessed a lot of development in finite mixture and non-parametric modelling in the context of one-sample tests, for example, the EM tests of Chen and Li (2009) and Chen et al. (2012), goodness of fit tests of Wichitchan et al. (2019), among others. However, not all these tests are applicable directly to the problem of OK testing in the context of a single case against multiple controls.

**Fig. 1.**
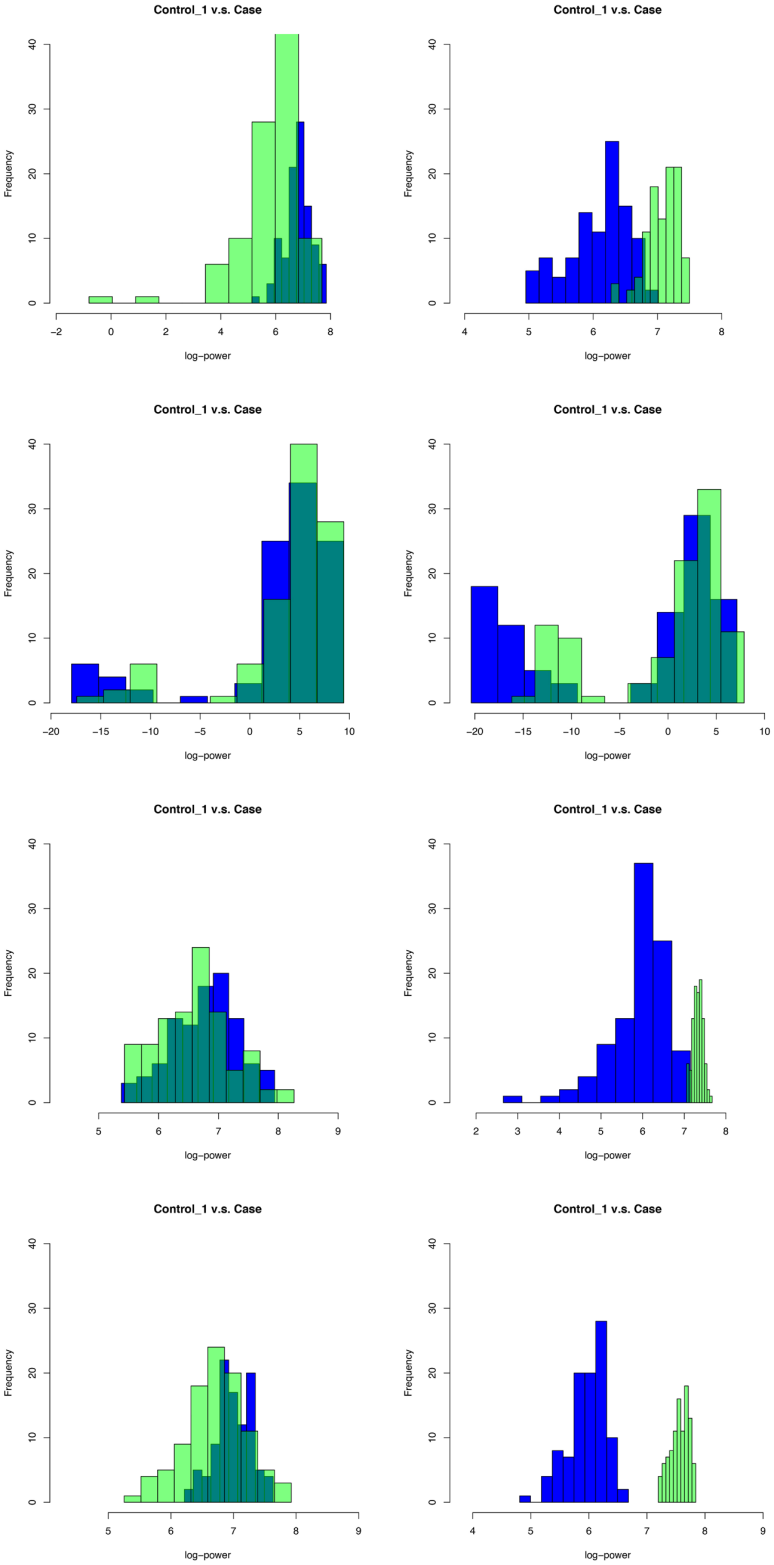
Histogram plots for a case and one of controls in the delta and gamma bands in areas 3 (ctx-lh-caudalmiddlefrontal), 6 (ctx-lh-frontalpole), 9 (ctx-lh-inferiortemporal), and 12 (ctx-lh-lateraloccipital). Row 1: delta and gamma bands in area 3. Row 2: delta and gamma bands in area 6. Row 3: delta and gamma bands in area 9. Row 4: delta and gamma bands in area 12. The blue and green histograms are for the control subject 1 and the case, respectively.

**Fig. 2.**
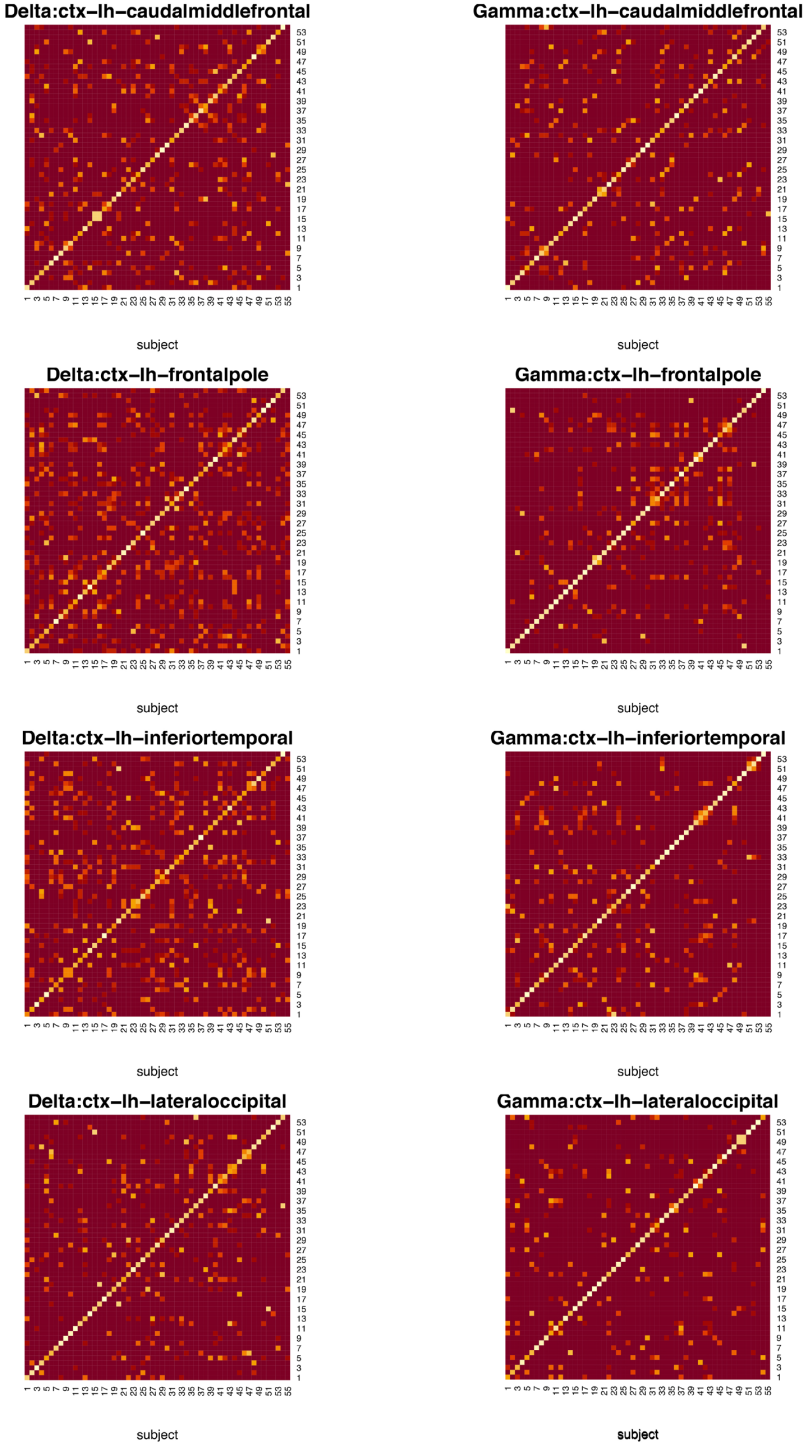
Heatmaps for (1-p)-values of pairwise Anderson–Darling tests between the controls and between the controls and the case. The controls are indexed by 1 to 54, while the case is indexed by 55 . Rows 1 to 4 are, respectively, for areas 3, 6, 9, and 12 as described in Figure 1. Each row displays the heatmaps of the (1-p)-values of pairwise Anderson–Darling tests for the delta and gamma bands from the left to the right. The colour changes from dark red to light white as (1-p)-value decreases from 1 to 0. Dark red indicates most significant p-values while the light colours stand for less significant p-values.

In a step toward understanding how delta- or gamma-band neural responses to brain injury, the present study concerns distribution changes of this adaptation in the context of source magnitudes/band powers with MEG scan data recently acquired by the Innovision IP Ltd. The data consist of MEG scans for a single testing subject and for an age-and-gender-matched control group of size K. In the data, according to the Desikan–Killiany Atlas, the brain was divided into A=68 functional regions of interest, indexed by 1, 2,…, 34 for the areas in the left hemisphere and by 35,…, 68 for the mirror areas in the right hemisphere (Desikan et al., 2006). Voxel-wise, MEG source magnitude/power data over grid points in different spectrum bands were calculated with these scans using the Fast-VESTAL. The average delta and gamma band powers were then calculated in each area and in each epoch for individual subjects, generating multiple A×N data matrices. Our exploratory data analysis on one of the above datasets raises the following questions for a further statistical analysis, necessitating the development of flexible and adaptable methodologies for the OK testing. First, the band power distributions are skewed and multimodal as shown by histogram plots in Figure 1. This raises the concern of robustness of the Crawford–Garthwaite test when the underlying distribution family is deviated from normals. Second, unlike the traditional case–control studies that requires the assumption of within-group homogeneity, in the current study, we compare a testing subject with a heterogeneous control group. The heterogeneity in the controls is manifested by pairwise AD tests for distributional shift displayed in Figure 2, where deep red highlights these p-values close to the low limit 0, whereas the white marks these p-values close to the up limit 1. The p-values increase from 0 to 1 in the brightness of colour. The first K=54 columns in the map demonstrate p-values derived from pairwise AD tests for each control against the remaining controls, respectively, while the last column shows p-values for the case against the controls. It can be seen from Figure 2 that for most of the pairs of subjects, the p-values derived from AD tests are close to 0 as the corresponding squares in Figure 2 have colours close to the red rather than yellow or white. In addition, for squares near diagonal line, they become white, which mean subjects have p-values of 1 when compared with themselves. These facts imply that some control subjects behave very similar to the case when they are tested against the remaining controls. For example, in Supplementary Figure S2, taking these pairwise p-values as similarity scores, we perform average linkage hierarchical clustering on K+1=55 subjects. The case will be expected to be the last subject merged into the dendrogram if the target brain area does differ the case from controls. In the cortical area 9 and in the gamma band, the case subject is the last to be merged in the dendrogram and well separated from most of the controls. However, the case behaves similar to the controls 35 , 27, and 54 in the above area. This finding is not by coincidence as similar phenomena are revealed in other cortical areas. The details are omitted. Furthermore, as anticipated, Supplementary Figure S2 indicates grouping structures in the controls in the areas 9 and 12 . Such a heterogeneity occurs in other brain conditions as the assumption of within-group homogeneity is reflected neither in clinical populations nor in the heterogeneous pathological nature of neurodegenerative diseases (Verdi et al., 2021). Third, permutation tests are increasingly being used as a reliable method for inference in neuroimaging (Winkler et al., 2016). For example, combining permutation techniques with AD test of a single case against multiple controls, we first pool the individual control samples into a single sample under the homogeneity assumption and then draw multiple random subsets of the same size as the case sample, against which the AD test is conducted for the case sample, obtaining multiple p-values. The testing is claimed significant at the level 0.01 if the average of these p-values is below 0.01. Unfortunately, the heterogeneity makes this permutation-based null model biased and causes the AD test over-sensitive to individual differences in the controls. This demonstrates that the failure to incorporate heterogeneity in inference may have a negative effect on the accuracy of diagnosis of brain conditions. In particular, for these group average-based studies (e.g., average case vs. average control), effects of heterogeneity have not been taken into account. For the current data, averaging non-diagonal entries for each column in the above heatmap shows that the average ((1-p)-value)/discrepancy between the case and the controls is larger than the within-group discrepancy of the controls. This suggests a possibility of adjusting the p-values in a single-case study to improve the accuracy of diagnosis. Fourthly, the concept of p-value has been widely used to measure the degree of discrepancy between the data and the null model. The significance of a traditional p-value is determined on a uniform scale as the p-value has a uniform null distribution. However, this uniformity deteriorates when the controls are heterogeneous. To remedy this difficulty, we need to develop a robust testing procedure that can automatically adjust the critical value when the controls are heterogeneous. For this purpose, we impose some penalty on non-uniformity of p-values when determining the critical value for the above diagnostic test. Finally, it is notoriously difficult to determine the null distribution of a mixture likelihood ratio test statistic as the classical Wilks’ asymptotic theory may not hold for mixture likelihood ratios. To overcome the difficulty, Dacunha-Castelle and Gassiat (1999) developed a local conic parametrisation approach for deriving the asymptotic distribution of a likelihood ratio test statistic. However, finding an explicit asymptotic distribution for a finite normal mixture model of unknown number of components is still an open problem.

Here, we develop a robust and flexible modelling strategy in which the case density is characterised by a finite normal mixture and the control density by a double mixture, which contains two layers: the first layer is for modelling subject-heterogeneity while the second layer is nested in the first layer for modelling heterogeneity within subjects. These models, allowing for different model dimensions in the case and controls, can explore the skewness and multi-modality in the data. Based on these models, we construct a novel likelihood ratio test for difference between the individual case and its age-matched control group. The critical value is automatically determined by imposing a bootstrap cross-validated penalty on p-value. We develop an asymptotic theory to support the proposed testing procedure. We apply the proposed procedure to the MEG scan data, reporting a list of brain damage areas for the patient and improving our understanding of the neuronal mechanism underpinning brain injury. To evaluate the performance of the new procedures, we compare the proposed likelihood ratio procedure with the average pairwise AD test, the pairwise permutated AD test, and the AD mean test by simulations. Overall, the proposed method shows its advantage over these AD methods, improving the existing methods by reducing not only false positive rate but also false negative rate even when the underlying distributions substantially deviate from normal mixtures.

The remaining paper is organised as follows. The details of the proposed methodology are provided in Section 2. The applications of the proposed methods to the brain injury dataset and synthetic data are presented in Section 3. The asymptotic theory is developed in Section 4. Discussion and conclusions are made in Section 5.

## 2 Methodology

Resting-state MEG and structural MRI image data with eyes open and eyes closed were collected for testing subjects following the Data Acquisition Protocol of the Innovision IP Ltd as follows. Prior to acquisition, empty room data were acquired so that correct noise removal procedures were exploited. In MRI T1 and T2 structural scans were required. The data were then sent to the Innovision IP secure server using encrypted file transfer protocols. Ethical permission for research use was also in place with informed consent for data usage for research in the anonymised format and approved by the ethics committee in the Innovision IP Ltd. The controls matching the testing subject’s gender and age within ± 5 years were obtained from the Cambridge Centre for Ageing and Neuroscience (Cam-CAN) study (Shafto et al., 2014). None of controls had a history of head trauma, neurological or neuropsychological disorders. Following the pipeline for analysis in the Innovision IP Ltd, the MEG data were pre-processed to identify artefacts and attenuate various sources of interference. Then OK tests were conducted on these data.

An OK test is implemented in two steps: We begin with a source magnitude imaging in frequency domain followed by performing case–control contrast tests. The testing results are adjusted for effects of heterogeneity by similarity analysis based on hierarchical clustering.

### 2.1 MEG source magnitude imaging

Let N be the total number of epochs and s the total number of sensors considered in the study. For epoch n, 1≤n≤N , consider J time points. Let $B_{nij}$ denote the measurement of sensor i at the jth time point, and ${\text{B}}_{nj}={\left(B_{n1j},...,B_{nsj}\right)}^{T}$ be all measurements at the jth time point in epoch n, 1≤j≤J . Let ${\text{Q}}_{nj}={\left(Q_{n1j},...,Q_{npj}\right)}^{T}$ be the magnitude vector of the candidate sources at grids $\left\{r_{1},...,r_{p}\right\}$ in the brain and $\left\{Q_{nkj}:\;1\leq j\leq J\right\}$ the source magnitude time course at location $r_{k}$ and epoch n. Following Zhang and Su (2015), assume that the true sources are approximately located on the grids when they are sufficiently dense (i.e., p is sufficiently large). Let $\text{G}=\left({\text{G}}_{1},...,{\text{G}}_{p}\right)$ denote the s×p gain matrix derived from unit inputs. Sarvas (1987) showed that the contribution of an individual source to ${\text{B}}_{j}$ can be numerically calculated by the use of a Maxwell’s equation-based forward model and that the contributions of multiple sources can be summed up linearly. Accordingly, we have the source model ${\text{B}}_{nj}=\text{G}{\text{Q}}_{nj}+\varepsilon_{nj},\;1\leq j\leq J,$ where 1≤p<∞ , $\varepsilon_{nj}$ is the background noise vector of the s sensors at time j. As pointed out before, brain activity is evidenced by the amount of oscillatory activity in different frequency bands. Therefore, it is necessary to transform source signals into frequency bands (Huang et al., 2012). For this purpose, we perform discrete Fourier transformation on both sides of the above equation in frequency band m, obtaining

$$\begin{array}{cccc} {\text{F}}_{nm}=\text{G}{\text{H}}_{nm}+{\text{e}}_{nm}, & & & \end{array}$$ (1)

with p-vector

$$\begin{array}{cccc} {\text{F}}_{nm} & = & \sum_{j=1}^{J}{\text{B}}_{nj}\text{exp}\left(-i2\pi mj/J\right),\;{\text{H}}_{nm}=\sum_{j=1}^{J}{\text{Q}}_{nj}\text{exp}\left(-i2\pi mj/J\right), & \\ {\text{e}}_{nm} & = & \sum_{j=1}^{J}\varepsilon_{nj}\text{exp}\left(-i2\pi mj/J\right), & \end{array}$$

where $i=\sqrt{-1}$ is a unit complex number. When p is much larger than the number of sensors, the model estimation becomes challenging as there are a diverging number of candidate models which can fit to the data. To circumvent the problem, Huang et al. (2012) developed the Fast-VESTAL MEG source imaging procedure by imposing $L_{1}$ restraints on the magnitude vector in Eq. (1). For epoch n, each area, and each band, calculate the average magnitude over the grids in the region and over the spectra in the band using the Welch’s method in the Scipy Python package (Virtanen et al., 2020).

Let $\text{Y}=\left(y_{ij}\right)={\left({\text{y}}_{1},...,{\text{y}}_{A}\right)}^{T}\;\in {\mathbb{R}}^{A\times N},{\text{X}}_{k}\;={\left({\text{x}}_{ak}\right)}_{1\leq a\leq A}\;\in {\mathbb{R}}^{A\times N},$ k=1,...,K are log-transformed band power data for a single case and K controls, respectively. Let ${\text{X}}_{a}={\left({\text{x}}_{ak}\right)}_{1\leq k\leq K}.$ Suppose that for region a, ${\left(y_{aj}\right)}_{1\leq j\leq N}$ is a sample drawn from the case density $f(y|\psi_{a0})$ and ${\left(x_{ajk}\right)}_{1\leq j\leq N}$ a sample drawn from the control density $f_{a}(x|\psi_{ak})$ , k=1,...,K, where $\psi_{0}$ and $\psi_{ak}$ are unknown parameters. Then, for region a, our research problem can be formulated as testing the hypotheses

$$\begin{array}{l} \begin{array}{cccc} H_{a0}:f(\cdot |\psi_{a0})\in \left\{f\left(\cdot \text{|}\psi_{ak}\right),\;1\leq k\leq K\right\} & & & \end{array}\\ \text{v.s. }H_{a1}:f(\cdot |\psi_{a0})\notin \{f(\cdot |\psi_{ak}),\;1\leq k\leq K\}. \end{array}$$ (2)

We consider the following four OK contrast tests.

### 2.2 Likelihood ratio test in frequency domain

As the likelihood ratio test is the most powerful test of a simple null hypothesis against a simple alternative hypothesis, the first OK test we proposed is the likelihood ratio test. In our problem setting, both the null density $f(\cdot |\psi_{a0})$ and the alternative $f(\cdot |\psi_{ak})$ are unknown. We are unable to use the likelihood ratio test directly. Note that histograms in Figure 1 have already demonstrated that $f_{a}(y|\psi_{a0})$ and $f_{a}(x|\psi_{ak})$ , k=1,...,K, can be well approximated by finite mixtures of normals. So, we can use the data to estimate these unknown likelihoods. Here, for each subject, using the R-package Mclust (Scrucca et al., 2023), we fit a finite mixture of normals to the data with order being estimated via Bayesian Informatic Criterion (BIC), estimating the maximum log-likelihood under the null and alternative hypotheses, respectively. Let $l_{a}({\hat{\psi }}_{a0}|{\text{y}}_{a})$ and $l_{a}({\hat{\psi }}_{ak}|{\text{x}}_{ak})$ , k=1,...,K be the estimated maximum log-likelihoods corresponding to the case and controls, respectively. To incorporate the null hypothesis in the test statistic, we consider the following frequency-band log-likelihood ratio test statistic

$$\begin{array}{cccc} \begin{array}{l} l_{a0k}=\underset{\psi_{a0}=\psi_{ak}}{\text{max}}l(\psi_{a0},\psi_{ak}|{\text{y}}_{a},{\text{x}}_{ak})\\ \;\;\;\;\;\;\;\;\;\;-\underset{\psi_{a0}}{\text{max}}l(\psi_{a0}|{\text{y}}_{a})-\underset{\psi_{ak}}{\text{max}}l(\psi_{ak}|{\text{x}}_{ak}), \end{array} & & & \end{array}$$

where $l(\psi_{a0},\psi_{ak}\;|\;{\text{y}}_{a},{\text{x}}_{ak})=l(\psi_{a0}\;|\;{\text{y}}_{a})+l(\psi_{ak}\;|\;{\text{x}}_{ak})$ , $l(\psi_{a0}\;|\;{\text{y}}_{a})$ and $l(\psi_{ak}|{\text{x}}_{ak})$ are, respectively, the joint and the individual log-likelihood functions based on the samples ${\text{y}}_{a}$ and ${\text{x}}_{ak}$ . The larger the ${\text{l}}_{a0k}$ , the higher the chance that $\psi_{a0}$ is equal to $\psi_{ak}$ . The p-value can then be estimated by

$$\begin{array}{cccc} {\text{p}}_{l}\left({\text{y}}_{a},{\text{x}}_{a}\right)=\frac{1}{K}\sum_{k=1}^{K}I\left(l_{a0k}\geq \text{log}\left(1-c_{0}\right)\right), & & & \end{array}$$

where the critical value $c_{0}$ is determined by an approximate null distribution of ${\text{l}}_{a0k}$ if the asymptotic null distribution is available. Note that the asymptotic null distribution of the above normal mixture-based test is unknown and may depend on the underlying null models. This makes it hard to set the critical value $c_{0}$. To tackle the issue, we cross-validate the above average p-values by use of the following one-out-of-K scheme. For each 1≤k≤K , we perform the above likelihood ratio test on ${\text{x}}_{ak}$ against the remaining samples, obtaining p-values ${\text{p}}_{flr}\left({\text{x}}_{ak},{\text{x}}_{am},\;1\leq m\neq k\leq K\right).$ The cross-validated pairwise likelihood ratio p-value (${\text{cp}}_{flr}({\text{y}}_{a},{\text{x}}_{a})$ ) is then calculated through counting the proportion of ${\text{p}}_{flr}\left({\text{x}}_{ak},{\text{x}}_{am},\;1\leq m\neq k\leq K\right)$ being larger than or equal to ${\text{p}}_{flr}\left({\text{y}}_{a},{\text{x}}_{a}\right).$ We choose $c_{0}$ by minimising ${\text{p}}_{l}\left({\text{y}}_{a},{\text{x}}_{a}\right)+{\text{cp}}_{flr}\left({\text{y}}_{a},{\text{x}}_{a}\right)$ with respect to $c_{0}$. However, it is hard to develop an asymptotic theory as given $\left({\text{y}}_{a},{\text{x}}_{a}\right),{\text{p}}_{flr}\left({\text{x}}_{ak},{\text{x}}_{am},\;1\leq m\neq k\leq K\right)$ are not conditionally independent. To fix this, we use the bootstrap resampling to cross-validate ${\text{p}}_{l}\left({\text{y}}_{a},{\text{x}}_{a}\right)$, obtaining the cross-validated p-value ${\text{cp}}_{l}\left({\text{y}}_{a},{\text{x}}_{a}\right).$ The details are as follows.

For each area a and each control subject 0≤k≤K , we generate a bootstrap sample ${\text{x}}_{a}^{\left(kb\right)}$ from estimated density $f_{a}(\cdot |{\hat{\theta }}_{k})$ . We calculating the p-value ${\text{p}}_{l}\left({\text{x}}_{a}^{\left(kb\right)},{\text{x}}_{a}\right)$ by performing the above likelihood ratio test on the bootstrap sample ${\text{x}}_{a}^{\left(kb\right)}$ against the controls. This provides a bootstrap estimate of the background scale for the observed p-value. We count the proportion of the cross-validated p-values which are at least significant as the observed p-value of the case ${\text{p}}_{l}\left({\text{y}}_{a},{\text{x}}_{a}\right)$, leading to the following cross-validated p-value

$$\begin{array}{cccc} {\text{cp}}_{l}\left({\text{y}}_{a},{\text{x}}_{a}\right)=\frac{1}{K}\sum_{k=1}^{K}I\left({\text{p}}_{l}\left({\text{x}}_{a}^{\left(kb\right)},{\text{x}}_{a}\right)\leq {\text{p}}_{l}\left({\text{y}}_{a},{\text{x}}_{a}\right)\right). & & & \end{array}$$

We choose the critical value $c_{0}$ by minimising $\left({\text{p}}_{l}\left({\text{y}}_{a},{\text{x}}_{a}\right)+{\text{cp}}_{l}\left({\text{y}}_{a},{\text{x}}_{a}\right)\right)$ with respect to $c_{min}\leq c_{0}\leq c_{max}.$ Here, we pre-choose $c_{min}$ and $c_{max}$ so that the size of the test at a pre-specified level, say, 0.01. We apply the Benjamini–Hochberg procedure to control false discovery rate for multiple testing.

### 2.3 Modified Anderson–Darling tests in frequency domain

We are testing multiple hypotheses in Eq. (2). Unlike before, we will not pre-specify the distributions $f_{ak},\;0\leq k\leq K,\;1\leq a\leq A.$ We consider the following nonparametric tests.

*Pairwise AD (PAD) test for a distributional shift.* For region a, 1≤a≤A , based on the R-package “two-samples” (Dowd, 2023), we perform the AD two-sample test of the case versus each control, obtaining K p-values. Denote the average of these p-values as ${\text{p}}_{adp}\left({\text{y}}_{a},{\text{x}}_{ak},\;1\leq k\leq K\right).$ We reject the null hypothesis $H_{a0}$ if the resulting p-value is less than or equal to a pre-specified level. We cross-validate the above average p-values by use of the following one-out-of-K scheme. For each 1≤k≤K , we perform the Anderson–Darling test on ${\text{x}}_{ak}$ against the remaining samples, obtaining p-values ${\text{p}}_{pad}\left({\text{x}}_{ak},{\text{x}}_{am},\;1\leq m\neq k\leq K\right).$ The cross-validated pairwise Anderson–Darling p-value ${\text{cp}}_{pad}$ is then calculated through counting the proportion of ${\text{p}}_{pad}\left({\text{x}}_{ak},{\text{x}}_{am},\;1\leq m\neq k\leq K\right)$ being larger than or equal to ${\text{p}}_{pad}\left({\text{y}}_{a},{\text{x}}_{a}\right).$

*AD permutation (PMAD) test for a distributional shift under the assumption of population homogeneity.* For region a, 1≤a≤A , we randomly draw N subsets, each of size J, from the pooled control samples $\left\{x_{akj}:\;1\leq j\leq J,\;1\leq k\leq K\right\}.$ We perform the two-sample AD test for each subset versus the case sample, obtaining N p-values. Denote the average of these p-values by ${\text{p}}_{ad}\left({\text{y}}_{a},{\text{x}}_{ak},\;1\leq k\leq K\right).$ As usual, we apply the Benjamini–Hochberg procedure to control false discovery rate for multiple testing. In this test, we implicitly use the homogeneity assumption for the control sample. We reject the null hypothesis $H_{a0}$ if the resulting p-value is less than or equal to a pre-specified level. Similarly, we can calibrate these p-values by using the one-out-of-K scheme.

*AD test for a mean shift.* The AD, applied to sample means, can be viewed as a non-parametric t-test. For region a, 1≤a≤A , we first calculate the averages ${\bar{y}}_{a}$ and ${\bar{x}}_{ak},\;1\leq k\leq K$ of the case and control samples over epochs. We perform the two-sample AD test for ${\bar{y}}_{a}$ versus ${\bar{x}}_{ak},\;1\leq k\leq K.$ We reject the null hypothesis $H_{a0}$ if the resulting p-value is less than or equal to a pre-specified level.

For all the above tests, as usual, we apply the Benjamini–Hochberg procedure to control false discovery rate for multiple testing.

### 2.4 Correction and visualisation for heterogeneity effects

**Fig. 3.**
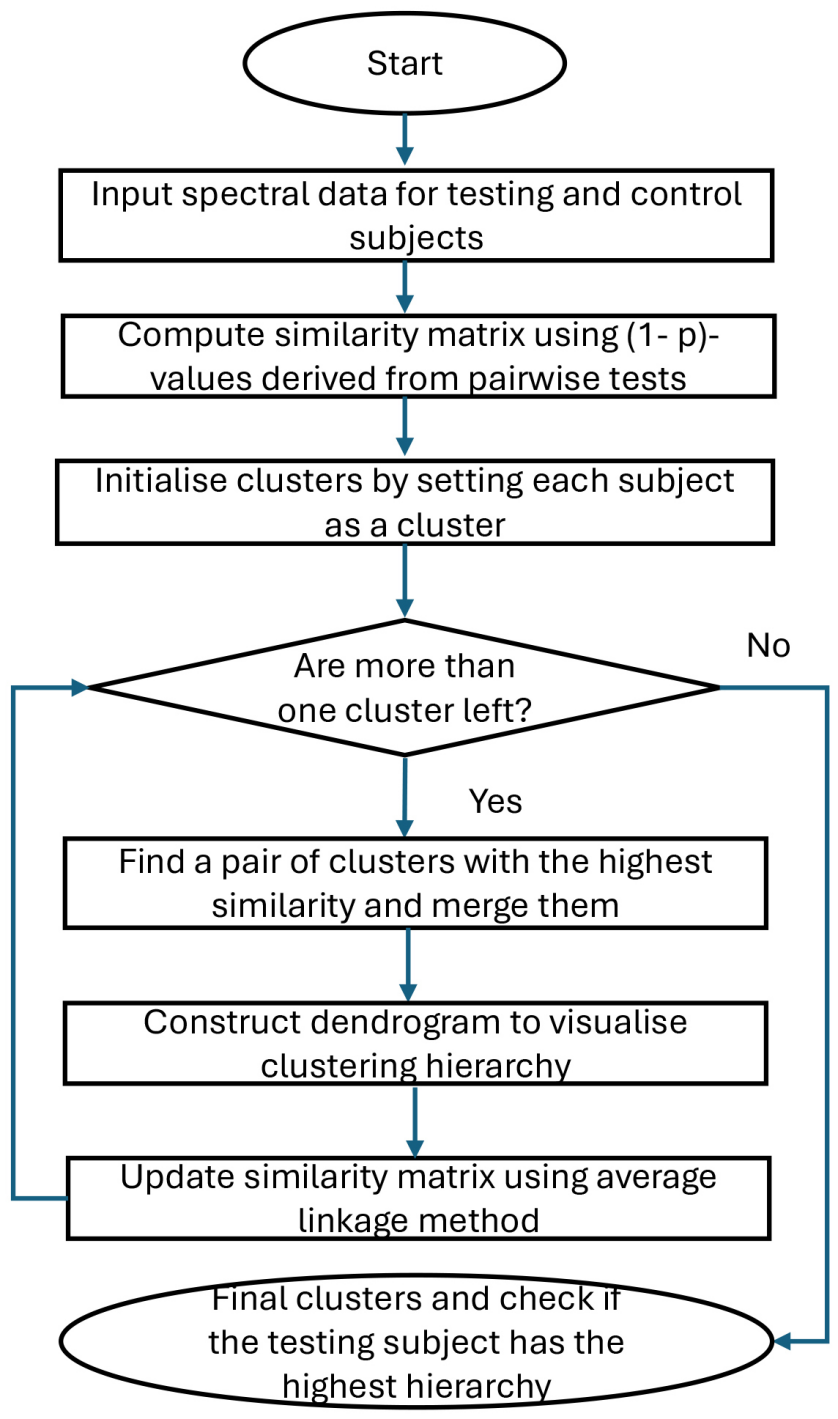
The HC flow-chart for the correction and visualisation of heterogeneity.

In the proposed FLR and PAD procedures, the p-value of an OK test, defined by averaging the p-values of the corresponding pairwise tests of the case against individual controls, can be affected by heterogeneity of controls. Failing to adjust for such effects may lead to biased diagnosis and wrong conclusions. To remove these effects, we group controls for each area, that is, clustering KN -dimensional vectors, which is ill-posed if K<N. The conventional methods such as k-means clustering and model-based clustering (Scrucca et al., 2023) may miss double mixture structures in the data. Here, we consider a hierarchical clustering (HC) strategy below: We first, for each area in the brain, calculate p-values for both case–control pairs and control–control pairs. This results in a (1-p)-value based (K+1)×(K+1) similarity matrix for K+1 subjects. We use a bottom-up approach to create an upside-down clustering tree called dendrogram: At the bottom, each subject starts in its own cluster. We repeat the following two steps until reaching the top hierarchy: (i) Calculate average similarity score for each pair of clusters. (ii) Find a pair of clusters with the maximum average similarity score, merge them as one, and move up the hierarchy. The case is expected to have the highest hierarchy in the dendrogram when the case is significantly different from the control group in terms of p-values. We claim the areas that the case has the highest hierarchy as HC-approved areas. See Figure 3 for a flow-chart for the proposed HC. Combining HC with FLR and PAD, respectively, we have heterogeneity-adjusted tests, FLR-HC and PAD-HC. We can visualise multiple testing with the dendrograms.

## 3 Numerical Results

In this section, using simulations and real data analysis, we evaluate the performance of the proposed likelihood ratio procedure FLR and compare it with the non-parametric competitors PAD, PMAD, and ADM in testing multiple hypotheses: For region a, 1≤a≤A , we test $H_{a0}:f_{a0}\in \left\{f_{ak},\;1\leq k\leq K\right\}$ v.s. the alternative $H_{a1}:f_{a0}\notin \left\{f_{ak},\;1\leq k\leq K\right\}$.

### 3.1 Synthetic data

Let the control group have the size K=54 and the epoch/sample size N=100 and 150 . For each case–control setting, we generated 50 independent datasets. Each dataset contains a case sample of size N drawn from $f(x|\psi_{0})$ , and K control samples of size N drawn from $f\left(x\text{|}\psi_{k}\right),1\leq k\leq K,$ respectively. Denote by $\phi (x|\mu ,\sigma^{2})$ the normal density with mean μ and variance $\sigma^{2}.$ Taking into account the patterns of skew, two modes, and heterogeneity in real MEG scan data displayed in Figure 1, we consider three settings. In Setting 1, we assess the proposed procedure in a favourable situation, where the underlying distributions belong to a normal-mixture distribution family. In Settings 2 and 3, we evaluate the performance of proposed procedures when the underlying distributions are miss specified, that is, they are outside the family of normal-mixture distributions. We employ performance metrics, precision, recall, and F scores, to compare the FLR with the other tests. Precision is the fraction of true $H_{1}$ instances among the claimed instances (i.e., among instances of p-value less than 0.05 ), where 1-precision is equal to false discovery rate. Recall (also known as sensitivity) is the fraction of claimed $H_{1}$ instances among all $H_{1}$ instances. F scores are measures that combine precision and recall. For example, the traditional $F_{1}$ is the harmonic mean of precision and recall. In general, we define $F_{w}=\left(1+w^{2}\right){\left(w^{2}/\text{recall}+1/\text{precision}\right)}^{-1}$ , where the weight 0≤w≤1 is chosen such that recall is considered w times as important as precision. Two commonly used values for w are 2, which weighs recall higher than precision, and 0.5 , which weighs recall lower than precision. See Saito and Rehmsmeier (2015).

*Setting 1 (Heterogeneous normal mixtures)*: *Controls*: $f\left(x\text{|}\psi_{k}\right)=0.2\phi \left(x\text{|}0,1\right)+0.8\phi \left(x\text{|}1,1\right),1\leq k\leq 10,$ and $f\left(x\text{|}\psi_{k}\right)=0.4\phi \left(x\text{|}0,1\right)+0.6\phi \left(x\text{|}1,1\right),11\leq k\leq K=54.$ In this setting, there are around 18% controls drawn from a two-component normal mixture and 82% controls drawn from a slightly different two-component normal mixture. The scenario imitates an empirical fact observed in Figure 1 that there may be two skew sub-populations in the controls, one with a relatively smaller size. Consider the following three case settings, respectively. *Case 1.1*: $f(x|\psi_{0})=0.2\phi (x|0,1)+0.8\phi (x|2,1).$ *Case 1.2*: $f(x|\psi_{0})=0.4\phi (x|0,1)+0.6\phi (x|1,1).$ *Case 1.3*: $f(x|\psi_{0})=0.1\phi (x|0,1.5)+0.9\phi (x|1,1).$ *Case 1.4*: $f(x|\psi_{0})=0.4\phi (x|0,2)+0.6\phi (x|1,2).$ *Case 1.5*: $f(x|\psi_{0})$ =0.2ϕ(x|−1,1)+0.8ϕ(x|3,1).

*Cases 1.1* and *1.2* are used to calculate the type I error rate of the test, where the null hypothesis $H_{0}$ is true, whereas *Cases 1.3*, *1.4*, and *1.5* are used to show the power of the test for a range of shifts. We consider the shifts in one of component variances and one of mixture weights in *Case 1.3*, in both component variances in *Case 1.4*, and in both component means in *Case 1.5*.

*Setting 2 (Homogeneous lognormal)*: *Controls*: $f\left(x\text{|}\psi_{k}\right)=\phi \left(x\text{|}0,1\right),1\leq k\leq K.$ We consider three scenarios, respectively. *Case 2.1*: $f(x|\psi_{0})=\phi (\text{ln}\left(x\right)|0,1)/x,x>0.$ *Case 2.2*: $f(x|\psi_{0})=\phi (\text{ln}\left(x\right)|0.5,1)/x,x>0.$ *Case 2.3*: $f(x|\psi_{0})=\phi (\text{ln}\left(x\right)|1,1)/x,x>0.$ Similar to Setting 1, *Case 2.1* is used to calculate the size of the test, where the null hypothesis $H_{0}$ is true, whereas *Cases 2.2* and *2.3* are used to show the power of testing for the location shifts from 0 to 0.5 and from 0 to 1, respectively.

**Fig. 4.**
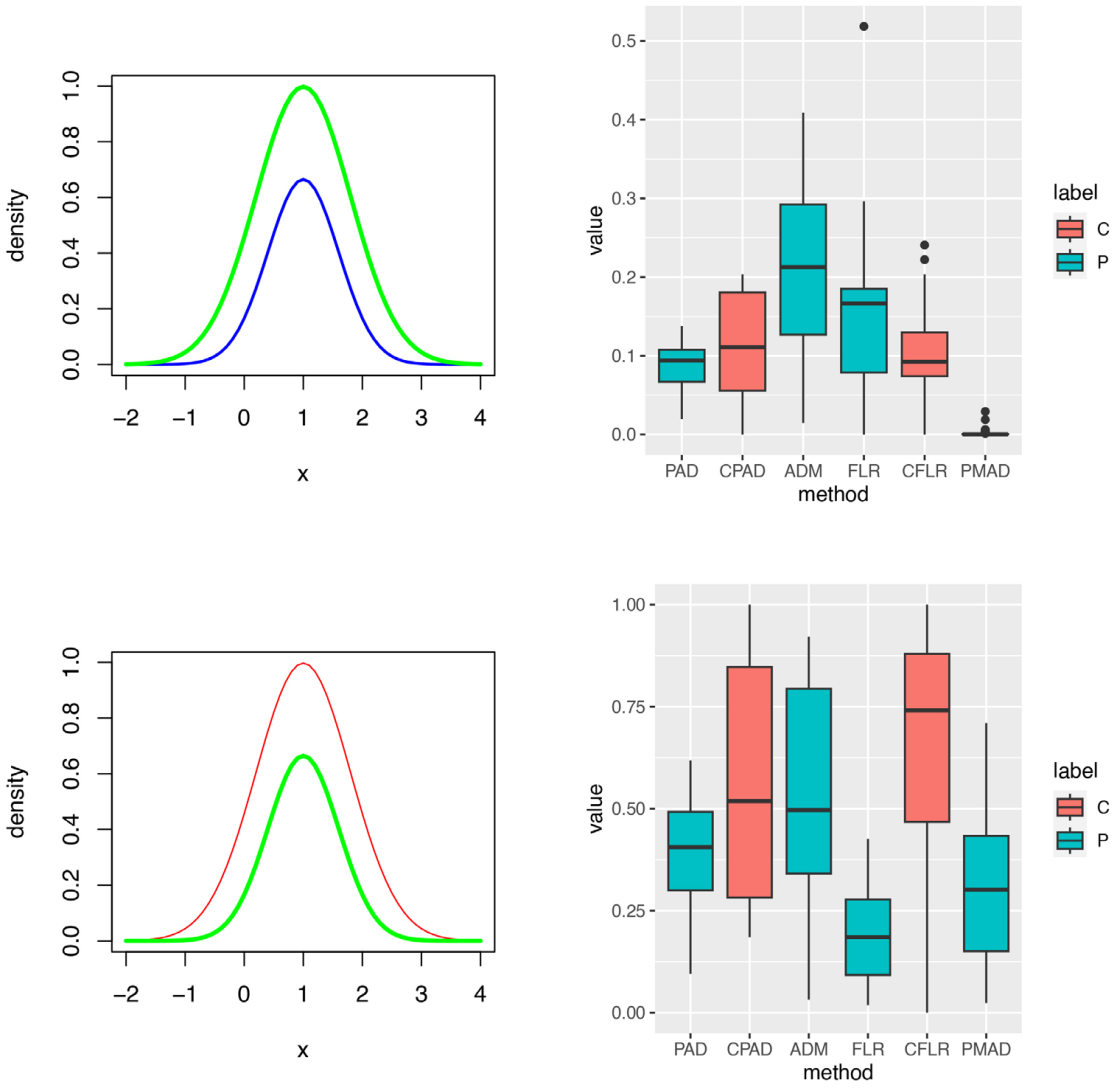
Heterogeneous normal mixtures (Setting 1: Cases 1.1 and 1.2). Left and right columns contain component density plots and p-value plots, respectively. Rows 1 to 2 are corresponding to testing each of Cases 1.1–1.2 against the Controls, respectively. Labels C and P in the right column stand for types of tests, cross-validation and principal, respectively. If the significance level of these tests was set to 0.05 , then estimated sizes of test: 0.16 for the PAD, 0.06 for the ADM, 0.12 for the FLR, and 1 for the PMAD.

**Fig. 5.**
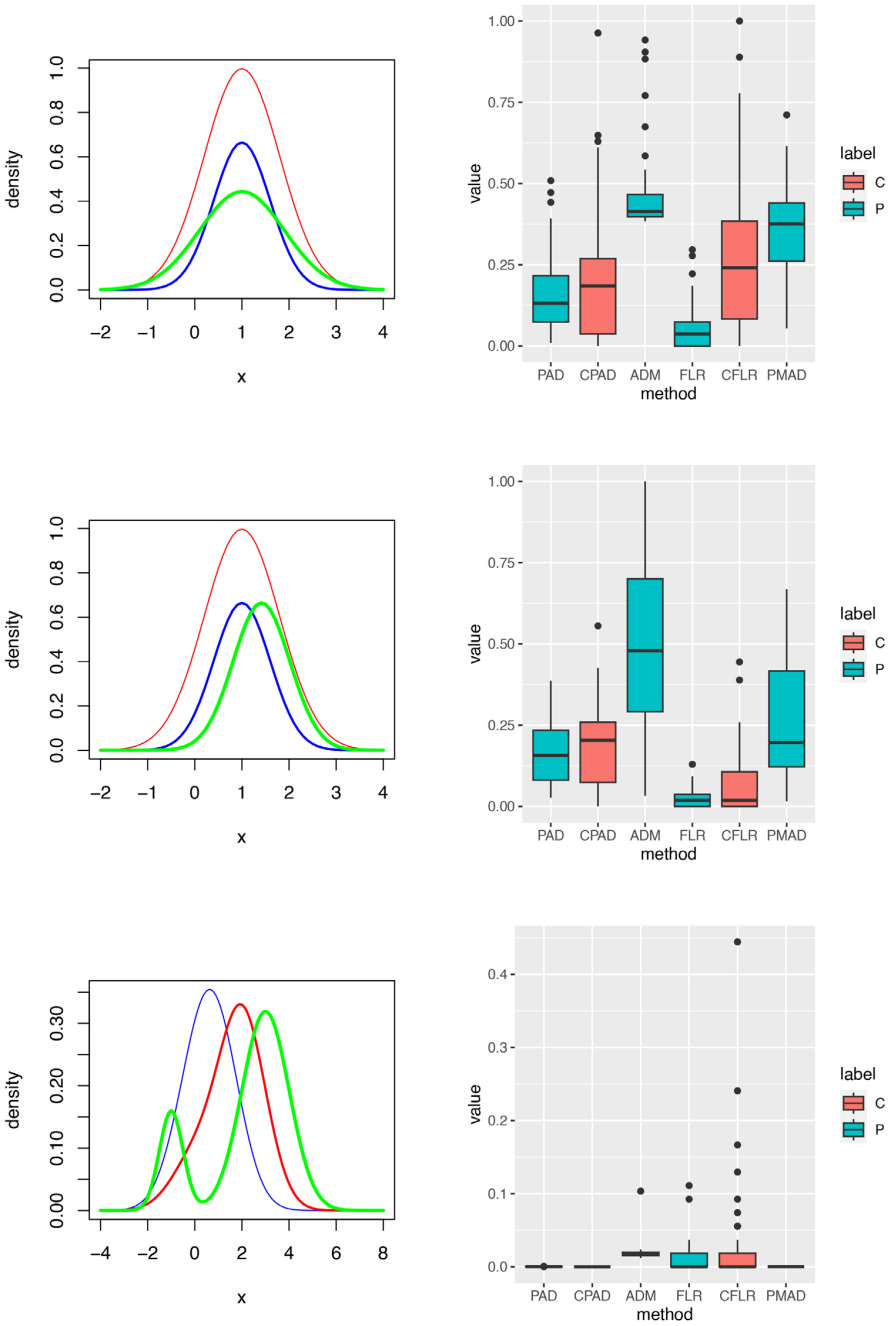
Heterogeneous normal mixtures (Setting 1: Cases 1.3, 1.4, and 1.5). Left and right columns contain component density plots and p-value plots, respectively. Rows 1 to 3 are corresponding to testing each of Cases 1.3–1.5 against the Controls, respectively. Labels C and P in the right column stand for types of tests, cross-validation and principal, respectively. P-values in the right column can be used to show the powers of these tests if the significance level was set to 0.05 .

**Fig. 6.**
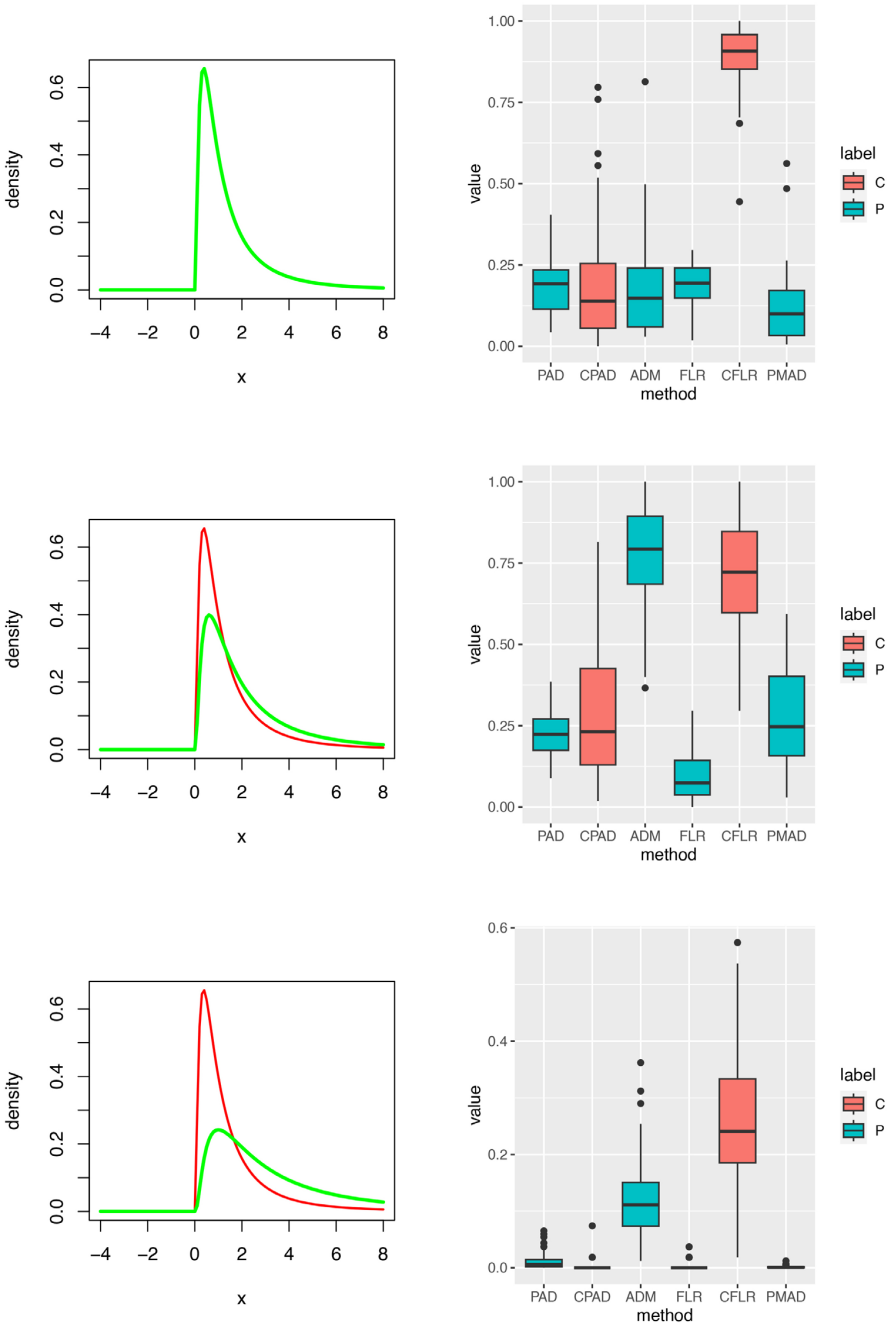
Homogeneous log-normals (Setting 2). Left and right columns contain component density plots and p-value plots, respectively. Rows 1 to 3 for Setting 2 are corresponding to testing each of 3 testing subjects against the Controls, respectively. The top right box plots can be used to calculate the sizes of these tests while p-values in the right column of Rows 2 and 3 can be used to show the powers of these tests. Labels C and P in the right column stand for types of tests, cross-validation and principal, respectively.

**Fig. 7.**
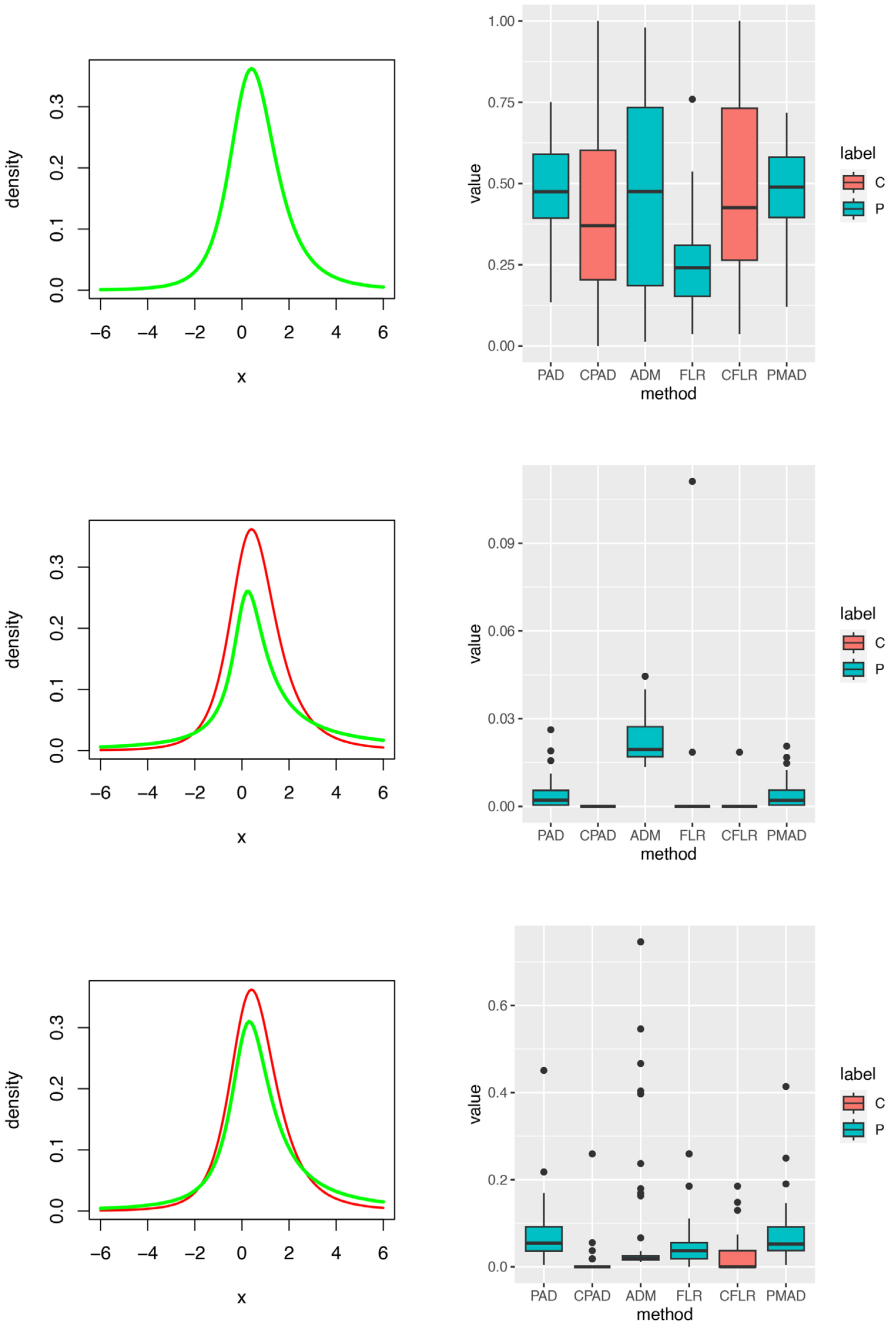
Homogeneous noncentral t-distributions (Setting 3). Left and right columns contain component density plots and p-value plots, respectively. Rows 4 to 6 for Setting 3 are corresponding to testing each of 3 testing subjects against the controls, respectively. The top right box plots can be used to calculate the sizes of these tests while p-values in the right column of Rows 2 and 3 can be used to show the powers of these tests. Labels C and P in the right column stand for types of tests, cross-validation and principal, respectively.

The resultant 50 p-values are plotted in Figures 4 to 7. The estimated percentages of p-values being less than or equal to 0.05 are calculated in Table 1 and Supplementary Table S1, where outliers have been screened out by the boxplots. These estimated precisions and recalls are quite robust. These numerical results show that the FLR achieves the best overall performance ($F_{1}$ score) among six tests and the PAD ranks the second place. Compared with the PAD, the FLR performs much better in terms of recall but slightly worse in terms of precision. In Setting 1, Figures 4 and 5 show that the FLR performed substantially better than the PAD, PMAD, and ADM: In terms of $F_{1}$ score, the FLR improved the PAD by 33% for N=100 and 14% for N=150 , the PMAD by 100% for N=100 and 78% for N=150 , and the ADM by 68% for N=100 and 64% for N=150 . In terms of $F_{0.5}$ score, the FLR improved the PAD by 2.3% for N=100 and 7% for N=150 , the PMAD by 91% for N=100 and 75% for N=150 , and the ADM by 10% for N=100 and 31% for N=150 . In terms of $F_{2}$ score, the FLR improved the PAD by 60% for N=100 and 19% for N=150 , the PMAD by 110% for N=100 and 80% for N=150 , and the ADM by 100% for N=100 and 119% for N=150 . In Setting 2, the FLR also outperformed the PAD, PMAD, and ADM: In terms of $F_{1}$ score, the FLR improved the PAD by 32%, the PMAD by 16% , and the ADM by 427% . In terms of $F_{0.5}$ score, the FLR improved the PAD by 25% , the PMAD by 20%, and the ADM by 256% . In terms of $F_{2}$ score, the FLR improved the PAD by 39% , the PMAD by 13%, and the ADM by 545% . In Setting 3, the PAD performed similar to the PAD and PMAD and better than the ADM, while the CPAD attains the best F-scores. However, the FLR performs better than the PAD, PMAD, and ADM in terms of $F_{1}$ scores, while the FLR performs slightly worse than the PAD, PMAD, and ADM in terms of $F_{0.5}$ scores. Similar results hold for N=150.

**Table 1.** Percentages of instances with p-value less than 0.05 and metrics: N=100 .

	METHOD					
Setting/Metrics	FLR	CFLR	PAD	CPAD	PMAD	ADM
1.1	0.12	0.2	0.16	0.22	1	0.06
1.2	0.12	0.02	0	0	0.06	0.08
1.3	0.6	0.2	0.2	0.28	0	0
1.4	0.78	0.58	0.12	0.2	0.08	0.08
1.5	0.96	0.82	1	1	1	0.98
Precision	**0.91**	0.88	0.89	0.87	0.50	0.88
Recall	**0.78**	0.53	0.44	0.49	0.36	0.35
$F_{1}$	**0.84**	0.66	0.63	0.63	0.42	0.50
$F_{0.5}$	**0.88**	0.78	0.86	0.75	0.46	0.80
$F_{2}$	**0.80**	0.58	0.50	0.54	0.38	0.40
2.1	0.04	0.2	0.02	0.2	0.32	0.2
2.2	0.32	0	0	0.08	0.2.3	
2.3	1	0.02	0.94	0.98	1	0.18
Precision	**0.97**	0.09	0.82	0.84	0.79	0.47
Recall	**0.66**	0.01	0.47	0.53	0.60	0.09
$F_{1}$	**0.79**	0.02	0.60	0.65	0.68	0.15
$F_{0.5}$	**0.89**	0.03	0.71	0.75	0.74	0.25
$F_{2}$	**0.71**	0.01	0.51	0.57	0.63	0.11
3.1	0.04	0.04	0	0.06	0	0.06
3.2	0.98	1	1	1	1	1
3.3	0.66	0.88	0.4	0.96	0.44	0.8
Precision	**0.98**	0.98	1	0.97	1	0.97
Recall	0.82	0.94	0.70	**0.98**	0.72	0.90
$F_{1}$	0.89	0.96	0.82	**0.97**	0.84	0.93
$F_{0.5}$	0.84	**0.97**	0.92	**0.97**	0.93	0.96
$F_{2}$	0.85	0.95	0.74	**0.98**	0.76	0.91

The bold values indicate they attained the maximum in the row which they belong to.

To demonstrate the superior performance of the FLR-HC over the PAD-HC, we generate a dataset for each of Cases 1.1 , 1.2, and 1.3 in Setting 1. We apply the FLR-based HC, FLR-HC, and the PAD-based HC, PAD-HC to these datasets, drawing the corresponding dendrograms. The testing subject is expected to be among the controls in Cases 1.1 and 1.2 in Setting 1, whereas the testing subject is expected to be outside the control group in Case 1.3 in Setting 1. In all these settings, the FLR has clearly identified three subgroups in the controls if we horizontally cut the dendrograms at the height of 0.99 , whereas the PAD can produce too many subgroups. Therefore, the results displayed in Supplementary Figures S2 and S3 in Section S3 indicate that the FLR-HC is more powerful than the PAD-HC in correctly capturing hidden heterogeneity and in predicting the group identity of a testing subject.

### 3.2 Real MEG scan data

As suggested by the simulation studies, the FLR performs better, in terms of F scores, than the PAD, PMAD, and ADM in testing a single case versus multiple controls. In this subsection, we applied the FLR, PAD, ADM, and PMAD to real single-subject studies.

In the literature, mild traumatic brain injury is often classified into three types: combat-related injury, sports-related injury, and general injury that has mixed etiologies including road traffic accident (RTA) and those who present to hospitals. Exposure to mTBI, both single and repeated, in battle fields, sports, and road traffics raised widespread concern over possible long-term consequences. We selected three single cases from a hundred cases which were originally investigated by the Innovision IP Ltd: a RTA-related mTBI (Case 1), a combat-related mTBI who had more recent exposure to blasts (Case 2), and a sport-related mTBI, to reflect different mTBI types recorded in our community. We aimed to identify clinical characteristics of these mTBIs.

We performed single-subject test for each case by applying the proposed procedures to its MEG scan data. In each case, the delta and gamma brain activities of the testing subject were compared with data from an age- and gender-matched control group of size K from a control population who had no known history of having had a brain injury (Shafto et al., 2014). Matching the age within ±5 years, we had K=54, 108, and 109 for the 3 cases, respectively. We applied the aforementioned tests to each case for the 68 areas (indexed by 1 to 34 in the left hemisphere and by 35 to 68 in the right hemisphere, see the Supplementary Materials) simultaneously, obtaining a list of p-values, one for each area. For the FLR, to estimate the cross-validated p-values, we also re-drew a bootstrap sample of size N=100 from the estimated mixture distribution of each control. To control the false discovery rate of multiple testing, we adjusted these p-values by using the Benjamini–Hochberg procedure (Benjamini & Hochberg, 1995). We also adjusted heterogeneity by performing the FLR-HC and PAD-HC on the spectral data. The significance level of these adjusted p-values was set to 0.01 . After adjustments for multiple testing and for heterogeneity, we revealed that the FLR identified more abnormal areas than PAD and ADM while PMAD was heavily impacted by heterogeneity in the controls. The mTBI severity was measured across the three cases in terms of the number of abnormal areas (13+24 for Case 1, 7+6 for Case 3, and 7+1 for Case 2) revealed by the FLR-HC. See Tables 2 and 3 and Supplementary Tables S2 to S5 for details. Note that many more abnormal areas would be claimed by the FLR-HC and PAD-HC if the significance level was set to 0.05 . Although the revealed abnormal areas were varying across the three cases, they did fall in vulnerable regions to mTBI reported in the literature, namely, the frontal lobe, temporal lobe, parietal lobe, occipital lobe, basal ganglia, diencephalon, corpus callosum, and hippocampus (Allen et al., 2021; Huang et al., 2023; Kaltiainen et al., 2019).

**Table 2.** The delta band data analysis for mTBI case 1.

METHODS	Hemisphere	Areas	Adj.p-values
FLR	lh	3, 4, 7, 8, 12, 14, 17, 19, 20, 23, 27, 32	<0.01
	rh	42–44, 46, 49, 50, 54, 58–60, 62, 63, 67	<0.01
FLR-HC	lh	3, 4, 8, 14, 27, 32	<0.01
	rh	42, 43, 49, 59, 60, 63, 67	<0.01
CFLR	lh	27, 32	<0.01
	rh	60, 61, 63	<0.01
PAD	lh	17, 25, 27, 28	<0.01
	rh	59, 63	<0.01
PAD-HC	lh	17, 27, 28	<0.01
	rh	59, 63	<0.01
CPAD	lh	17, 25, 27, 28	<0.01
	rh	59, 61	<0.01
PMAD	lh	1–12, 14–34	<0.01
	rh	36–44, 46, 47, 49, 50, 52, 53, 55–68	<0.01
ADM	lh	None	<0.01
	rh	None	<0.01

**Table 3.** The gamma band data analysis for mTBI case 1.

METHODS	Hemisphere	Areas	Adj.p-values
FLR	lh	1–3, 5–7, 9, 11, 12, 14, 15, 18, 19, 24, 27–31, 33, 34	<0.01
	rh	35–37, 39, 40, 43, 45, 48–51, 57, 59, 61, 63, 65, 66	<0.01
FLR-HC	lh	2, 5–7, 9, 11, 12, 14, 18, 24, 27, 28, 31, 34	<0.01
	rh	36, 40, 43, 45, 48, 51, 57, 59, 61, 66	<0.01
CFLR	lh	5, 6, 9, 11, 12, 14, 24, 27, 28	<0.01
	rh	36, 43, 48, 59, 61, 66	<0.01
PAD	lh	1, 2, 4, 9, 12, 14, 16, 17, 19, 23–25, 27, 28, 30–32	<0.01
	rh	36–38, 40, 48, 49, 53, 56, 57, 59–62, 64, 66, 67	
PAD-HC	lh	2, 9, 12, 14, 17, 24, 25, 27, 28, 30	<0.01
	rh	36, 38, 40, 48, 56, 59, 60, 61	<0.01
CPAD	lh	9, 17, 18, 24, 27, 31	<0.01
	rh	48, 56	<0.01
PMAD	lh	1–9, 11, 12, 14–19, 21–33	<0.01
	rh	36–68	<0.01
ADM	lh	None	<0.01
	rh	None	<0.01

#### 3.2.1 mTBI case 1

**Fig. 8.**
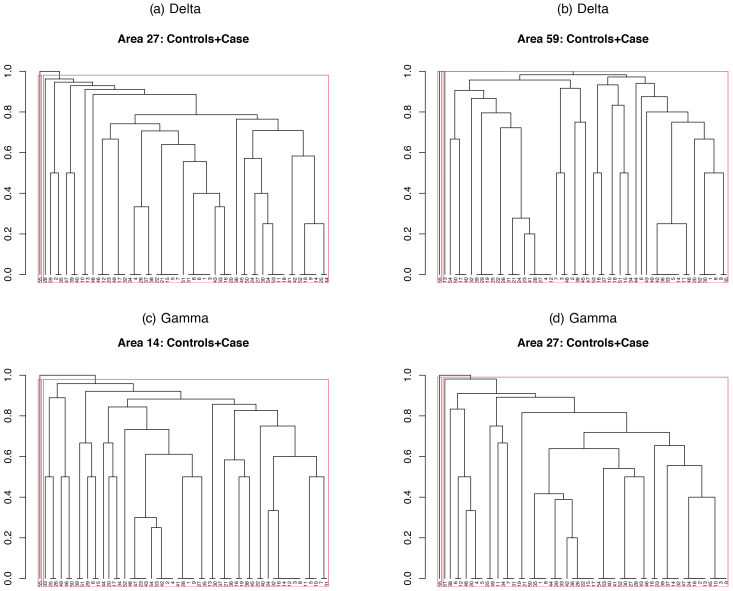
Examples of the FLR-HC dendrograms for the delta- and gamma-band data. Red boxes indicate the cluster borders if we partition 55 subjects into 2 clusters. (a) and (b) are for the delta band while (c) and (d) are for the gamma band.

*Delta band data analysis.* As pointed out before, we first applied the FLR procedure to the delta band data, followed by the Benjamini–Hochberg adjustment. Thresholding these p-values by 0.01 gave a list of significantly abnormal areas. We then corrected this list for heterogeneity effects by the FLR-HC. See, for example, Figure 8 and Supplementary Figures S8 and S9 for details. More details are omitted. According to the FLR-HC, in areas 7, 12, 17, 19, 20, 23, 44, 46, 50, 54, 58, and 62, similar scores between the case and some controls were bigger than the average similarity score within the control group due to subject-heterogeneity. Filtering out these areas, the FLR-HC, in Table 2, declared 13 abnormal areas: Areas 3, 49, and 63 in the frontal lobe, areas 43 and 67 in the temporal lobe, areas 8, 32, and 42 in the parietal lobe, areas 4 and 60 in the cuneus, area 59 in the paracentral, area 27 in the cingulate, and area 14 in the lingual area. In some of these areas, their dendrograms did show subgrouping of the controls. For example, in the delta band and area 59 , there were two subgroups in the controls if we cut the dendrogram at the height of 0.99 .

After the Benjamini–Hochberg procedure-based adjustment, the CFLR gave abnormal areas 27 and 61 in the cingulate, area 32 in the parietal lobe, area 60 in the cuneus, and area 63 in the frontal lobe (see Table 2).

The PAD was applied to the delta band data, followed by the Benjamini–Hochberg adjustment. Thresholding these adjusted p-values at the level 0.01 gave a list of significant abnormal areas. We then adjusted this list for heterogeneity effects by PAD-HC (see Supplementary Figures S4 to S6). The results, summarised in Table 2, showed 5 abnormal areas: Areas 17 and 59 in the paracentral areas, area 27 in the cingulate area, and areas 28 and 63 in the frontal lobe, where areas 27 , 59, and 63 were also identified by the FLR-HC. The CPAD, after the Benjamini–Hochberg procedure-based correction, claimed the following abnormal areas: the paracentral area 17 , the frontal lobe area 28 , and the cingulate areas 27 and 61 . In the delta band and areas 17 and 27 , there were at least three potential subgroups in the controls. The results showed that PAD-HC and CPAD identified a less number of abnormal areas than the FLR-HC and the CFLR.

In the delta band, the ADM had not found any abnormal areas at the level 0.01 after the Benjamini–Hochberg adjustment for multiple testing. However, after the Benjamini–Hochberg adjustment, the PMAD found 53 abnormal areas: 1−12, 14−34,  36−44,  46,  47,  49, 50, 52, 53, and 55−68, many more than found by the FLR-HC and the PAD-HC. This implies that the PMAD was too sensitive to subject-hetrogeneity than the FLR-HC and the PAD-HC.

*Gamma band data analysis.* The FLR was applied to the gamma band data, followed by the Benjamini–Hochberg adjustment. Thresholding these p-values by 0.01 , the FLR gave a list of significantly abnormal areas. We then corrected this list for heterogeneity effects by the FLR-HC as before. See, for example, Supplementary Figures S4, S5, S7, S10 and S11 for details. After FLR-HC filtering, in Table 3, 24 areas were left: Areas 6, 28, and 40 in the *frontal lobe*, areas 9, 43, 31, and 34 in the *temporal lobe*, areas 51, 57, and 59 the *central* areas, area 12 in the *occipital*, areas 14 and 48 in the *lingual*, area 7 in the *fusiform*, areas 2, 11, 24, 27, 36, 45, and 61 in the *cingulate* areas, area 18 in the parahippocampal, area 5 in the entorhinal, and area 66 in the supramarginal area. The CFLR claimed abnormalities in 15 areas: 6 and 28 in the frontal lobe areas, 2, 11, 24, 27, 36, and 61 in the cingulate, 9 and 43 in the temporal lobe, 12 in the occipital, 14 and 48 in the lingual, 59 in the central area, and 66 in the supramarginal area. Again, in some of these areas, their dendrograms did show potential subgrouping of the controls. For example, in the delta band and areas 14 and 27 , there were at least two potential subgroups in the controls.

The PAD gave 33 areas which were significant at the level of 0.01 after the Benjamini–Hochberg adjustment for multiple testing. Among them areas 1, 4 , 16 , 19 , 23 , 31 , 32 , 37 , 49 , 53 , 57 , 62 , 64 , 66, and 67 had been filtered out by the PAD-HC due to subject-heterogneity. Taking area 49 as an example, PAD-HC dendrogram Supplementary Figure S11 demonstrated that compared with control subjects 38 , 20, 17, and 42 , subject 55 was closer to the remaining 50 controls although as a case it significantly differed from the controls overall. After the PAD-HC filtering, 18 areas were left: Areas 28 , 30, and 40 in the *frontal lobe*, area 9 in the *temporal lobe*, area 59 in the *central* area, area 12 in the *occipital*, areas 14 and 48 in the *lingual*, areas 2, 24, 27, 36, and 61 in the *cingulate*, areas 38 and 60 in the precuneus and cuneus, areas 17 and 25 in the central areas, and area 56 in the pericalcarine. The details are omitted here. The CPAD claimed 8 abnormal areas: 9, 17, 18, 24, 27, 31, 48, and 56. The PMAD claimed 63 abnormal areas, many of which might be false positive due to being too sensitive to subject-heterogeneity in the controls. Similar to the FLR, in the gamma band and areas 14 and 27 , the PAD also showed some potential subgroups in the controls.

**Fig. 9.**
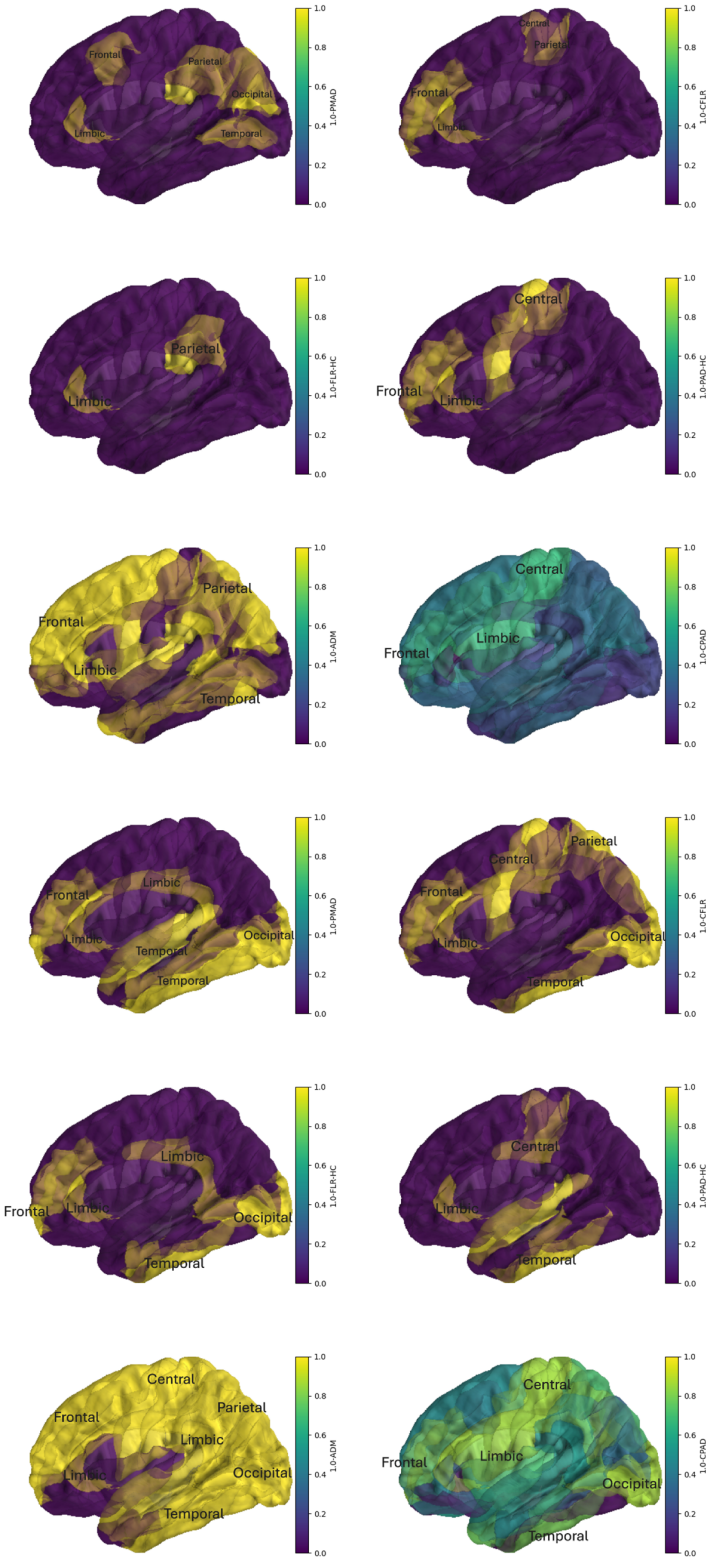
Plots of the adjusted (1-p)-values on left vertical areas for FLR-HC, PAD-HC, CFLR, CPAD, PMAD, and ADM, respectively. They can be divided into two blocks. Block 1 (rows 1 to 3) for the delta band while block 2 (rows 4 to 6) for the gamma band. In each block, from the left to the right and the top to the bottom, the plots are made in the order FLR-HC, PAD-HC, CFLR, CPAD, PMAD, and ADM. The detected abnormal areas are highlighted in gold.

**Fig. 10.**
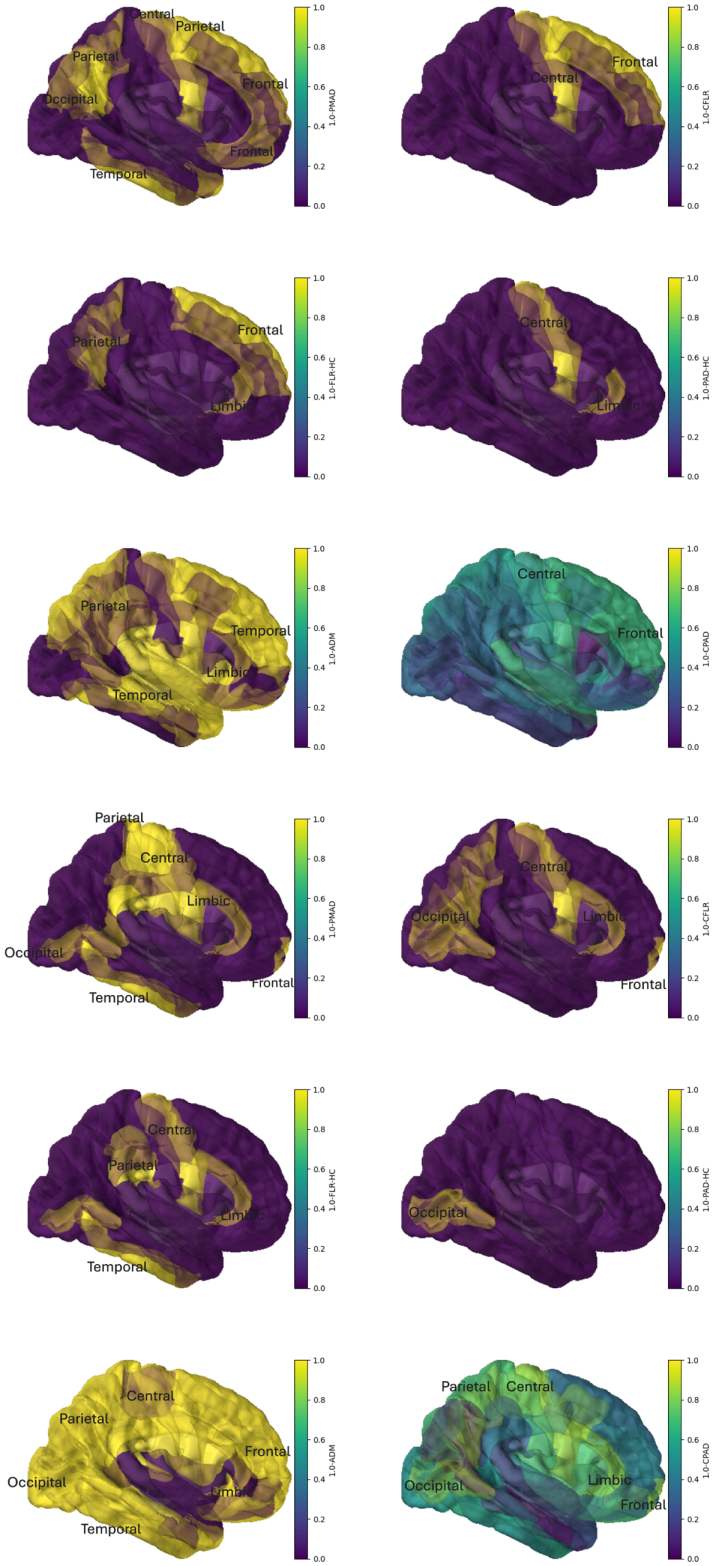
Plots of the adjusted (1-p)-values on right vertical areas for FLR-HC, PAD-HC, CFLR, CPAD, PMAD, and ADM, respectively. They can be divided into two blocks. Block 1 (rows 1 to 3) for the delta band while block 2 (rows 4 to 6) for the gamma band. In each block, from the left to the right and the top to the bottom, the plots are made in the order FLR-HC, PAD-HC, CFLR, CPAD, PMAD, and ADM. The detected abnormal areas are highlighted in gold.

The above differences among the FLR, PAD, PMAD, and ADM are clearly shown in Figures 9 and 10, the (1−p)-value plots on the brain vertex. Based on the delta and/or gamma band data, the above FLR-HC and PAD-HC analysis implies that when mTBI incurred, brain damages may be found in the frontal, occipital, parietal, and temporal lobes, and in cingulate gyrus, paracentral, precuneus, cuneus, lingual, fusiform, parahippocampal gyrus, and entorhinal cortex. In particular, there were more damaged areas claimed in the gamma band than in the delta band. Many abnormal activities were detected in precuneus and cuneus in the delta band than in the gamma band. The frontal lobe, sitting at the front and top of the brain, is responsible for the highest levels of thinking and behaviour, such as planning, judgement, decision making, impulse control, and attention. The frontal lobe contains the pars opercularis while paracentral contains parts of both the frontal and parietal lobes. The parietal lobe lying behind the frontal lobe takes in sensory information and helps an individual understand their position in their environment. The temporal lobe in the lower front of the brain has strong links with visual memory, language, and emotion. The temporal lobe contains temporal hole; superior, middle, and inferior temporal gyrus; parahippocampal/entorhinal gyri; and fusiform gyrus. The occipital lobe at the back of the brain processes visual input from the eyes, which includes precuneus, cuneus, lingual gyrus, and inferior occipital gyrus. The paracentral lobule has motor and sensory functions related to the lower limb. These facts suggest some expected changes in patient’s behaviour when there were damages in these lobes.

The above findings partially re-discovered what were found in Huang et al. (2014; 2023). Huang et al. (2014) showed that prefrontal, posterior parietal, inferior temporal, hippocampus, and cerebella areas were particularly vulnerable to brain trauma, and that MEG slow-wave generation in prefrontal areas positively correlated with personality change, trouble concentrating, affective lability, and depression symptoms. Huang et al. (2023) found that in both delta and gamma bands, the spatial differences in MEG activity in frontal and temporal lobes between a paediatric mTBI group and an orthopaedic injury control group were detected.

#### 3.2.2 mTBI case 2

We followed the same pipeline of data analysis as in Case 1 to test Case 2. The key findings, summarised in Supplementary Tables S2 to S3, are highlighted as follows.

*Delta band data analysis.* At the significance level of less than 0.01, the FLR-HC identified abnormal areas 14, 26, 30, 35, 44, 54, and 64. These are, respectively, the lingual, precuneus, and superiorparietal in the left hemisphere, and the bankssts, insula, parsorbitalis, and superiorparietal in the right hemisphere. At the significance level of less than 0.01, the PAD-HC identified abnormal areas 27, 30, 44, 60, and 64. Areas 27 and 60 are rostralanteriorcingulate in the left hemisphere and precuneus in the right hemisphere. The HC-dendrograms of these areas are provided in Supplementary Figures 12 to 18. The above findings re-discovered area insula found by Namkung et al. (2017).

*Gamma band data analysis.* At the significance level of less than 0.01, the FLR-HC identified abnormal area 28, rostralmiddlefrontal in the left hemisphere. The PAD-HC suggested abnormal areas 2 (caudalanteriorcingulate in the left hemisphere) and 36 (caudalanteriorcingulate in the right hemisphere).

#### 3.2.3 mTBI case 3

We again followed the same pipeline of data analysis as in Case 1 to test Case 3. The key findings, summarised in Supplementary Tables S4 to S5, were highlighted as follows. These findings were also aligned with the published reports on delta waves in injured individual brains (Huang et al., 2014, 2020).

*Delta band data analysis.* At the significance level of less than 0.01, the FLR-HC identified abnormal areas 1, 11, 21, 27, 37, 46, and 61. These are, respectively, the bankssts, isthmuscingulate, parstriangularis, and rostralanteriorcingulate in the left hemisphere, and the caudalmiddlefrontal, lateraloccipital, and rostralanteriorcingulate in the right hemisphere. At the significance level of less than 0.01, the PAD-HC indicated none of the areas were significantly abnormal. However, at the significance level of 0.05 , the PAD-HC did identify abnormal areas 11, 14, and 36. These areas are, respectively, isthmuscingulate and lingual in the left hemisphere and caudalanteriorcingulate in the right hemisphere. The HC-dendrograms of these areas were shown in Supplementary Figures S19 to S25.

*Gamma band data analysis.* At the significance level of less than 0.01, the FLR-HC identified areas 16, 27, 42, 45, 50, and 52. These areas are, respectively, middletemporal and rostalanteriorcingulate in the left hemisphere, and inferiorparietal, isthmuscingulate, middletemporal, and parahippocampal in the right hemisphere. At the significance level of 0.01, the PAD-HC suggested none of the areas were significantly abnormal. But at the significance level of 0.05 , the PAD-HC did suggest that area 36, caudalanteriorcingulate in the right hemisphere was significantly abnormal. These marked areas are also related to cognitive functioning of the brain.

In the literature, persisting impairment was evident in the sports-related mTBIs despite their better recovery compared with general mTBIs, due to younger age, less severe injuries, and many injuries going unrecognised. Here, the elevated delta- and gamma-wave areas detected by the FLR-HC had provided an additional evidence against taking an overoptimistic view of outcomes after exposures to sports-related mTBI (Ntikas et al., 2024; Sahler & Greenwald, 2012).

## 4 Theory

As the null distribution of the FLR test is difficult to calculate, the nominal significance level cannot be achieved precisely. In this section, coupled with simulation studies, we carry out a theoretical study on its asymptotic null distribution. Under certain regularity conditions, we show that for each nominal significance level α, a critical value $\text{log}(1-c_{0})$ can be identified to achieve the level asymptotically.

### 4.1 Regular distribution families

Log-normal distribution family and non-central t-distribution family are regular in the sense that the Fisher information matrix is positive definite when the shape parameter is away from zero. Skew distribution families such as skew t-distribution family are also regular. See Azzalini and Capitanio (2014). Log-normal, non-central t-distribution and skew distribution families only allow for skewness but not for multi-modality in the data. Nevertheless, we can state an asymptotic null-distribution for the likelihood ratio tests when both the case and controls are drawn from regular-distribution families as follows.

### 4.2 Two-sample test

Consider the following hypothesis

$$\begin{array}{cccc} H_{0}:f\left(\cdot \text{|}\psi_{0}\right)=f\left(\cdot \text{|}\psi_{1}\right)\;\text{v.s. }H_{1}:f(\cdot |\psi_{0})\neq f(\cdot |\psi_{1}), & & & \end{array}$$ (3)

where $f(\cdot |\psi_{k})$ depends on d-dimensional parameter $\psi_{k},k=0,\;1.$ Suppose that we have an i.i.d. sample ${\text{X}}_{k}$ drawn from $f(\cdot |\psi_{k})$ . Let $l_{x_{ki}}(\psi_{k})=\text{log}(f(x_{ki}|\psi_{k}))$ be the likelihood function. Suppose that the likelihood function has a second order derivative which is continuous and that the Fisher information matrix is strictly positive definite. Define the maximum likelihood ratio test statistic

$$\begin{array}{l} W_{n}\left({\text{X}}_{0},{\text{X}}_{1}\right)=\underset{\psi_{0}=\psi_{1}}{\max}\sum_{k=0}^{1}\sum_{i}l_{x_{ki}}\left(\psi_{k}\right)\\ \;\;\;\;\;\;\;\;\;\;\;\;\;\;\;\;\;\;\;\;\;-\underset{\psi_{0}}{\max}\sum_{i}l_{x_{0i}}\left(\psi_{0}\right)-\underset{\psi_{1}}{\max}\sum_{i}l_{x_{1i}}\left(\psi_{1}\right). \end{array}$$

Then, the following proposition follows from a similar arguments used to prove Wilks’ theorem.

**Proposition 1.** *Under the above regularity conditions,* $-2W_{n}\left({\text{X}}_{0},{\text{X}}_{1}\right)$ *converges to* $\chi_{d}^{2}$ *in distribution as the sample size tends to infinite, where* $\chi_{d}^{2}$ *is a chi-squared distribution with* d*-degrees of freedom.*

### 4.3 OK test

The above result can be extended to the one-vs-K-sample likelihood ratio test under some regularity conditions. For this purpose, consider the following hypothesis:

$$\begin{array}{cccc} \begin{array}{l} H_{0}:f\left(\cdot \text{|}\psi_{0}\right)\in \left\{f\left(\cdot \text{|}\psi_{k}\right):\;1\leq k\leq K\right\}\;\\ \;\;\;\text{v.s. }H_{1}:f(\cdot |\psi_{0})\notin \{f(\cdot |\psi_{k}):\;1\leq k\leq K\}, \end{array} & & & \end{array}$$ (4)

where $f(\cdot |\psi_{k})$ depends on d-dimensional parameter $\psi_{k},\;0\leq k\leq K$ , and $\psi_{k}$, 1≤k≤K is an i.i.d. sample drawn from a hyperparameter distribution. Suppose that we have an i.i.d. sample ${\text{X}}_{k}$ drawn from $f(\cdot |\psi_{k})$ , 0≤k≤K. Then, the following proposition follows from a similar argument to prove Wilk’s theorem. The proposition provides a way to determine an asymptotic critical value at the level α.

**Proposition 2.** *Assume that the above regularity conditions holds. For a nominal level* 0<α<1, *under* $H_{0}$*, for a large* K*, we have*

$$\begin{array}{ccc} W_{n}\left({\text{X}}_{0},{\text{X}}_{k}\right) & \to & \chi_{dk}^{2},\;1\leq k\leq K,\;in\;distribution.\\ \hat{p}\left(c_{0}\right) & = & \sum_{k=1}^{K}I\left(W_{n}\left({\text{X}}_{0},{\text{X}}_{k}\right)\geq \text{log}\left(1-c_{0}\right)\right)/K\to \sum_{k=1}^{K}I(-0.5\chi_{dk}^{2}\geq \text{log}\left(1-c_{0}\right))/K\\ & \approx & P\left(-0.5\chi_{d}^{2}\geq \text{log}\left(1-c_{0}\right)\right)=1-\alpha . \end{array}$$

### 4.4 Normal-mixture distribution family

Unlike skew distribution families, normal mixtures can allow for both multi-modality and skewness in the data. However, Wilks’ theorem no longer holds for normal mixtures, as they can be irregular in the sense that the Fisher information matrix is degenerate. In the following, using the technique of Dacunha-Castelle and Gassiat (1999), we develop the asymptotic null distribution for the proposed FLR test statistic and an empirical way to determine the critical value in the proposed test. We find that the asymptotic null distributions depend on the underlying order of mixture models. To ease the presentation, we begin with single-sample tests as follows.

#### 4.4.1 Single-sample test

Let $G_{p}$ denote the set of p-mixtures of normals, g(x|ψ) in the form $g\left(x\text{|}\psi \right)=\sum_{i=1}^{p}\pi_{i}\phi \left(x\text{|}\eta_{i}\right),1\leq p\leq p_{max},$ where parameters $\theta_{i}=\left(\mu_{i},\sigma_{i}^{2}\right)\in \mathbb{R}\times {\mathbb{R}}^{+},1\leq i\leq p$ , $\sum_{i=1}^{p}\pi_{i}=1$ and $\psi =(\pi_{1},\ldots ,\pi_{p},\eta_{1},\ldots ,\eta_{p})$ is the vector of all the parameters in the mixture. Let $g_{0}\left(x\right)=g(x|\psi^{0})=\sum_{i=1}^{p}\pi_{i}^{0}\phi (x|\eta_{i}^{0})$ denote the underlying mixture. Let $E_{0}$ denote the expectation operator under density $g_{0}$. Assume that

$$\begin{array}{l} \left(\text{C}0\right):g\left(x\text{|}\psi^{0}\right)\;\text{is identifiable up to }\\ \;\;\;\text{a permutation of components}. \end{array}$$

Suppose that we have a sample X of size n, drawn from the unknown density g(x|ψ). We want to test $H_{0}:g(x|\psi )=g_{0}\left(x\right)$ v.s. $H_{1}:g(x|\psi )\neq g_{0}\left(x\right).$ To tackle the model identifiability issue in G, following Dacunha-Castelle and Gassiat (1999), we locally reparametrise g(x|ψ) around $\psi_{0}$ by a perturbation of $g_{0}$ in the form

$$\begin{array}{cccc} \begin{array}{l} g(x|\theta ,\beta )=\sum_{i=1}^{p-p_{0}}\frac{\lambda_{i}\theta }{n\left(\beta \right)}\phi (x|\eta_{i})\\ \;\;\;\;\;\;\;\;\;\;\;\;\;\;\;\;\;+\sum_{l=1}^{p_{0}}\left(\pi_{i}^{0}+\frac{\rho_{l}\theta }{n\left(\beta \right)}\right)\phi \left(x|\eta_{l}^{0}+\frac{\theta \delta_{l}}{n\left(\beta \right)}\right), \end{array} & & & \end{array}$$

where $\theta \in [0,\theta_{g}]\subset {\text{R}}^{+}$ is an identifiable parameter, while $\beta =(\lambda_{1},\ldots ,\lambda_{p-p_{0}},\delta_{1},\ldots ,\delta_{p_{0}},\rho_{1},\ldots ,\rho_{p_{0}})$ contains non-identifiable parameters. It can be shown that g(x|θ,β) is a proper density function if β∈ℬ with

$$\begin{array}{cccc} ℬ & = & \{\beta :\lambda_{i}\geq 0,\eta_{i}\in \text{R}\times {\text{R}}^{+},\;1\leq i\leq p-p_{0};\delta_{l}\in \text{R}\times {\text{R}}^{+}, & \\ & & \begin{array}{l} \rho_{l}\in \text{R},\;1\leq l\leq p_{0};\\ \sum_{i=1}^{p-p_{0}}\lambda_{i}+\sum_{l=1}^{p_{0}}\rho_{l}=0;\sum_{i=1}^{p-p_{0}}\lambda_{i}^{2}+\sum_{l=1}^{p_{0}}\rho_{l}^{2}+\sum_{l=1}^{p_{0}}{\left\|\delta_{l}\right\|}^{2}=1\}, \end{array} & \end{array}$$

where n(β) is a normalisation factor such that $E_{0}\left[{\left(\frac{\partial g(X|0,\beta )}{\partial \theta }\right)}^{2}\right]=1$ . Letting l(x|θ,β)=log(g(x|θ,β), we have $\frac{\partial l(x|0,\beta )}{\partial \theta }=\frac{\partial g(x|0,\beta )}{\partial \theta }/\;g_{0}\left(x\right)$. Note that $E_{0}\left[\frac{\partial l(X|0,\beta )}{\partial \theta }\right]=0$ . Letting ▽ denote the gradient operator, we have the directional Fisher information $I{\left(0,\beta \right)}^{2}=E_{0}\left[{\left(\frac{\partial l(x|0,\beta )}{\partial \theta }\right)}^{2}\right]=-E_{0}\left[\frac{\partial^{2}l(x|0,\beta )}{\partial \theta^{2}}\right]=1$ when the squared normalising factor $n{\left(\beta \right)}^{2}$ satisfies

$$n{\left(\beta \right)}^{2}=E_{0}\left[{\left(\sum_{i=1}^{p-p_{0}}\lambda_{i}\phi (X|\eta_{i})+\sum_{l=1}^{p_{0}}\rho_{l}\phi \left(X\text{|}\eta_{l}^{0}\right)+\sum_{l=1}^{p_{0}}\pi_{l}^{0}\delta_{l}^{T}▽\phi (X|\eta_{l}^{0})\right)}^{2}g_{0}{\left(X\right)}^{-2}\right].$$

Assume that

(C1): For 1≤i≤p , $\eta_{l}\in \Gamma$ , a compact set of $\text{R}\times {\text{R}}^{+}$, and $\sigma_{l}^{2}$ is uniformly bounded below from 0.

Following Dacunha-Castelle and Gassiat (1999) and Keribin (2000), under Conditions (C0) and (C1), for $g\in G_{p}$, we restrict its conic parameters β to a compact set. Then we find a small interval $\left[0,\theta_{g}\right]$ for θ and define a conic neighbourhood of $g_{0}$, $\left\{g(x|\theta ,\beta ):\theta \in [0,\theta_{g}]\right\}$ . Define ${\hat{p}}_{x}={\text{argmax}}_{1\leq p\leq p_{max}}(W_{np}\left(X\right)-0.5\text{log}\left(n\right)\left(3p-1\right)$, a BIC estimator of order $p_{0}$. Keribin (2.30) proved that as the sample size n tends to infinity, ${\hat{p}}_{x}\to p_{0}.$ Define $D_{p}$ as the set of functions of form

$$d(x|\beta )=\frac{1}{n\left(\beta \right)g_{0}\left(x\right)}\left(\sum_{i=1}^{p-p_{0}}\lambda_{i}\phi (x|\eta_{i})+\sum_{l=1}^{p_{0}}\rho_{l}\phi (x|\eta_{l}^{0})+\sum_{l=1}^{p_{0}}\pi_{l}^{0}\delta_{l}^{T}▽\phi (x|\eta_{l}^{0})\right),\beta \in ℬ.$$

Define the log-likelihood ratio $W_{np}\left(X\right)={\text{sup}}_{g\in G_{p}}$ $\sum_{i=1}^{n}\text{log}\left(g\left(X_{i}\right)/g_{0}\left(X_{i}\right)\right).$ Let $W_{1}\left(d\right)$ be a Gaussian process indexed by D with covariance defined by the usual $L_{2}$ product. Let I(⋅) be an indicator. Then, we have

$${\left(W_{np}\left(X\right)\right)}_{1\leq p\leq p_{max}}\to 0.5{\left(\underset{d\in D_{p}}{\text{sup}}W_{1}{\left(d\right)}^{2}I\left(W\left(d\right)\geq 0\right)\right)}_{1\leq p\leq p_{max}}$$

in distribution as n tends to infinity. Using Slutsky’s theorem, we have, as n tends to infinity,

$$W_{n{\hat{p}}_{x}}\left(X\right)\to 0.5\underset{d\in D_{p_{0}}}{\text{sup}}W_{1}{\left(d\right)}^{2}I\left(W_{1}\left(d\right)\geq 0\right)=0.5\underset{d\in D_{p_{0}}}{\text{sup}}W_{1}{\left(d\right)}^{2}$$

in distribution. Note that the last equality follows from the fact that $D_{p_{0}}$ is a symmetric set.

#### 4.4.2 Two-sample test

Suppose that we have two samples $\text{X}=\left(X_{1},\ldots ,X_{n}\right)$ and $\text{Y}=\left(Y_{1},\ldots ,Y_{n}\right)$ generated from $g(x|\psi_{x})$ and $g(x\text{|}\psi_{y}),$ respectively. We want to test the null hypothesis $H_{0}:g(x|\psi_{x})=g(x|\psi_{y})$ . Define the following two-sample log-likelihood test statistic

$$\begin{array}{c} W_{np}\left(\text{Y},\text{X}\right)=\underset{g\in G_{p}}{\text{sup}}\sum_{i=1}^{n}\text{log}\left(g\left(X_{i}\right)g\left(Y_{i}\right)\right)-W_{np}\left(\text{X}\right)-W_{np}\left(Y\right). \end{array}$$

Let

$$\begin{array}{cccc} {\hat{p}}_{y} & = & {\text{argmax}}_{1\leq p\leq p_{max}}W_{np}\left(\text{Y}\right)-0.5\text{log}\left(n\right)\left(3p-1\right), & \\ {\hat{p}}_{x,y} & = & {\text{argmax}}_{1\leq p\leq p_{max}}W_{np}\left(\text{Y},\text{X}\right)-0.5\text{log}\left(2n\right)\left(3p-1\right), & \\ W_{n\hat{p}}\left(\text{Y},\text{X}\right) & = & \underset{g\in G_{{\hat{p}}_{x,y}}}{\text{sup}}\sum_{i=1}^{n}\text{log}\left(g\left(X_{i}\right)g\left(Y_{i}\right)\right)-W_{n{\hat{p}}_{x}}\left(\text{X}\right)-W_{n{\hat{p}}_{y}}\left(\text{Y}\right). & \end{array}$$

Similar to before, we can show that ${\hat{p}}_{x},{\hat{p}}_{y},$ and ${\hat{p}}_{x,y}$ all converge to $p_{0}$ in probability. Furthermore, let $\left\{\left(W_{1}\left(d\right),W_{2}\left(d\right)\right):d\in G_{p}\right\}$ denote two independent Gaussian process with covariance matrix defined by $L_{2}$ product as before. Let $W_{1p_{0}}$ denote $0.5\;{\text{sup}}_{d\in D_{p_{0}}}{\left(W_{1}\left(d\right)+W_{2}\left(d\right)\right)}^{2}-\;{\text{sup}}_{d\in D_{p_{0}}}W_{1}{\left(d\right)}^{2}$ $-{\text{sup}}_{d\in D_{p_{0}}}W_{2}{\left(d\right)}^{2}.$ Then, we have:

**Proposition 3.** *Under the conditions (C0) and (C1), as* n *tends to infinity,*

$$\begin{array}{cccc} \left(W_{np}\left(\text{Y},\text{X}\right),W_{np}\left(\text{X}\right),W_{np}\left(\text{Y}\right)\right) & \to & 0.5\;\left(\underset{d\in D_{p_{0}}}{\text{sup}}{\left(W_{1}\left(d\right)+W_{2}\left(d\right)\right)}^{2}I\left(W_{1}\left(d\right)+W_{2}\left(d\right)\geq 0\right)\right), & \\ & & \underset{d\in D_{p_{0}}}{\text{sup}}W_{1}\left(d\right)I\left(W_{1}\left(d\right)\geq 0\right),\underset{d\in D_{p_{0}}}{\text{sup}}W_{2}\left(d\right)I(W_{1}\left(d\right)\geq 0)), & \end{array}$$

*and*

$$\begin{array}{cccc} W_{n\hat{p}}\left(\text{Y},\text{X}\right) & \to & 0.5\underset{d\in D_{p_{0}}}{\text{sup}}{\left(W_{1}\left(d\right)+W_{2}\left(d\right)\right)}^{2}I\left(W_{1}\left(d\right)+W_{2}\left(d\right)\geq 0\right) & \\ & & -\underset{d\in D_{p_{0}}}{\text{sup}}W_{1}{\left(d\right)}^{2}I\left(W_{1}\left(d\right)\geq 0\right)-\underset{d\in D_{p_{0}}}{\text{sup}}W_{2}{\left(d\right)}^{2}I\left(W_{2}\left(d\right)\geq 0\right))=W_{1p_{0}} & \end{array}$$

*which depends on* $p_{0}$*.*

**Proof:** It follows from Dacunha-Castelle and Gassiat (1999), Keribin (2000) and Slutsky’s theorem.

#### 4.4.3 OK test

In a single-case study, we aim to test a single subject again m controls. The case density and control densities are modelled by normal mixtures g(x|ψ) and $g(y|\psi_{k})$ , 1≤k≤K, respectively, where $g(y|\psi_{k})$ is assumed to have the order $p_{k}∼\pi (q),\;1\leq q\leq p_{max}$ . Suppose that we have samples of size n for the case and controls, say Y, ${\text{X}}_{1}$, …, ${\text{X}}_{K}$. The null hypothesis $H_{0}$ is that the case comes from the control group. For each pair $\left(\text{Y},{\text{X}}_{k}\right)$, we construct a likelihood ratio test statistic $W_{n{\hat{p}}_{k}}\left(\text{Y},{\text{X}}_{k}\right)$. For any $c_{0}$, count the number of times that $W_{n{\hat{p}}_{k}}\left(\text{Y},{\text{X}}_{k}\right)$ is larger than or equal to $\text{log}\left(1-c_{0}\right)$, and define a p-value by $\hat{p}\left(c_{0}\right)=\sum_{k=1}^{K}I\left(W_{n{\hat{p}}_{k}}\left(\text{Y},{\text{X}}_{k}\right)\geq \text{log}\left(1-c_{0}\right)\right)\;/K.$ We have:

**Proposition 4.** *Under the conditions (C0) and* (C1)*, for large* K*, as the sample size tends to infinity,*

$$\begin{array}{ccc} \hat{p}\left(c_{0}\right) & \to & \sum_{k=1}^{K}I\left(W_{p_{k}}\geq \text{log}\left(1-c_{0}\right)\right)/K\approx \int P\left(W_{q}\geq \text{log}\left(1-c_{0}\right)\right)d\pi \left(q\right) \end{array}$$

*in probability.*

**Proof:** As in the previous subsections, under Conditions (C0) and (C1), we show that under $H_{0},{\left(W_{n{\hat{p}}_{k}}\left(\text{Y},{\text{X}}_{k}\right)\right)}_{1\leq k\leq K}$ converges to ${\left(W_{p_{k}}\right)}_{1\leq k\leq K}$ in distribution. The result follows straightforward.

#### 4.4.4 Bootstrap cross-validation

Quantifying uncertainties in the estimated p-value $\hat{p}(c_{0})$ is important in determining the tuning constant $c_{0}$. A common approach to such an uncertainty quantification is using bootstrap samples to estimate how extreme the estimated p-value is compared with its bootstrapped null distribution.

To derive the bootstrapped null distribution, we need to modify Condition (C0) as follows:

(C0a): There is a small Kullback–Leibler neighbourhood of $\phi (x|\psi_{0})$ in which the normal mixture ϕ(x|ψ) is identifiable.

**Proposition 5.** *Under Conditions (C0a) and (C1), the bootstrap p-value* $\hat{\text{cp}}(c_{0})$ *will convergence to a* $c_{0}$*-dependent limit in probability.*

**Proof:** It follows from the uniform convergence theorem of empirical processes. See van der Vaart (1998).

Propositions 4 and 5 imply that $\underset{c0}{\arg\min}\left(\hat{p}\left(c_{0}\right)+\hat{cp}\left(c_{0}\right)\right)$ will converge to its theoretical value under certain regularity conditions.

## 5 Discussion and Conclusion

Modelling and testing complex resting-state MEG scan data for abnormality in an mTBI patient are challenging due to high subject variability and nonspecificity of posttraumatic symptoms, for example, when differentiating between mild cognitive impairment and normal ageing-induced cognitive decline. There has been a significant surge in using MEG source imaging to find abnormal regions in an mTBI patient (Allen et al., 2021; Huang et al., 2021; Itälinna et al., 2023, among others).

### 5.1 Nature of single-subject studies

Commonly used hypothesis tests for finding diagnostic biomarkers of mTBI are based on group means, regarding individual differences as errors or noises. These statistical tests, implicitly assuming homogeneity within the case–control groups, are fundamentally oriented to comparing the “average case” against “average control.” In particular, in a recent group study, Huang et al. (2023) reached a sensitivity of 95% and a specificity of 90% in pediatric mTBI when combining delta and gamma band specific features under a traditional case–control framework. However, their findings may not be generalisable to single-subject studies, where a single case is compared with a group of potentially heterogeneous controls as demonstrated in this paper. In this paper, we have developed mixture-model-based likelihood ratio tests in frequency domain for testing a single subject against a group of healthy but heterogeneity controls.

### 5.2 Sensor-level analysis

Brain oscillations at different frequency bands were revealed as promising biomarkers for differentiating mTBI patients from healthy controls (e.g., Huang et al., 2014 and Kaltiainen et al., 2018). Aaltonen et al. (2023) showed their overall effect on the classification of sub-acute mTBI subjects by using sensor-level MEG power spectra, combined with machine learning techniques. The contribution of different bands to the classification was measured by performing the above analysis by adding one band at a time in a random order. The conventional spectral data analysis often converted time series data from the whole time domain to the whole frequency domain (Aaltonen et al., 2023; Kaltiainen et al., 2018). Dividing sensor time series into a number of epochs, Kaltiainen et al. (2019) calculated an average cross-spectral density matrix over these epochs. Their sensor- and source-level analyses based on the above matrix might compromise the efficiency of these analyses due to not taking into account varying distribution features in each frequency band. In this paper, we have studied the resting-state MEG data which are stationary. Beyond resting-state MEG, task-based MEG has also proven useful in detecting mTBI, where the MEG data may be non-stationary (Da Costa et al., 2015). There were a few spectral studies on non-stationary sensor-level analysis. von Sachs (2020) provided a recent survey on this topic. In particular, Maharaj (2002) developed an evolutionary spectra-based permutation test for differences between two non-stationary time series by segmenting these series into approximately uncorrelated time windows. Dette & Paparoditis (2009) proposed a class of bootstrap frequency domain tests in multivariate time series. It is possible to extend our epoch-based likelihood ratio approach to these non-stationary and multivariate settings but it is beyond the scope of this paper.

### 5.3 Source-level analysis

As pointed out, single-subject diagnosis aims at identifying personalised abnormal areas in the brain to aid in clinical decision making for individual mTBI patients. Patients can be in post-injury periods varying from a few days to a few years. This requires a source-level analysis in a clinical environment, where it is difficult to separate activity originating from close-by sources (Kaltiainen et al., 2019). In this paper, focusing on source-level analysis, we have proposed a double-mixture-based likelihood ratio testing procedure by using the existing R-software such as Mclust (Scrucca et al., 2023) along with a modified pairwise Anderson–Darling type test. To alleviate the effect of heterogeneity on statistical significance of a test, we have introduced a cross-validation-based calibration of the resulting p-values and a hierarchical clustering-based visualisation and correction for subject-heterogeneity. Using the proposed hierarchical clustering, we can view the variability of the control population related to the testing subject. To understand the behaviour of the proposed procedure, we have also established an asymptotic theory for the test statistic. The proposed brain-area-wise tests can be easily implemented in a paralleled way to improve their scalability when a large control group was involved. This facilitates its integration into a diagnostic workflow in practice.

By real data applications and simulation studies, we have shown a strong performance of the proposed testing procedure. In particular, we have demonstrated that the proposed likelihood ratio test can substantially outperform the conventional nonparametric tests such as the Anderson–Darling tests in a wide range of scenarios. By hierarchical-clustering-based visualisation, we have demonstrated why the proposed FLR performs better than the PAD in terms of ability in separating a case from heterogeneous controls. Based on the MEG source localisation in the delta and gamma band, using the proposed likelihood ratio test and the modified Anderson–Darling test, we have shown that abnormal brain areas in mTBI patients can be detected when compared with healthy controls with an overall accuracy, $F_{1}$ score around 82% , even in the presence of data skewness, multimodality, and subject-heterogeneity in the case and controls. In the real data analysis, we have demonstrated that the proposed likelihood ratio test is more sensitive in finding abnormal areas in the brain than the other methods such as the pairwise Anderson–Darling test, the permuted Anderson–Darling test, and the Anderson–Darling test on mean shifts. The regions which were found significant at the level 0.01 are located in the frontal, occipital, paracentral, parietal, and temporal lobes and in cingulate gyrus and cuneus of the brain.

We have shown that it is likely that the control group includes subjects whose brain activities in some areas are barely distinguishable from those of an mTBI patient. This implies that visualising the inter-individual variability in cases and controls is very important for improving the accuracy of diagnosis for an mTBI subject. Note that increased neural oscillatory activities in the delta and gamma bands are most frequent finding in mTBI patients (see Allen et al., 2021 and reference therein). The results obtained from the real data analysis are thus in line with the literature. Note that interpretability of a prediction in a medical context is important due to safety concern: clinicians want to minimise possible errors in the prediction. So, revealing heterogeneity in controls highlights the need to focus on abnomalities at an individual level rather than a typical mTBI patient as in a traditional case–control paradigm.

### 5.4 Heterogeneity of mTBIs

We have selected three different types of mTBI to evaluate the performances of the FLR-HC and PAD-HC at the significance level 0.01 . The testing subject in Case 1 is a road traffic accident (RTA) mTBI. In road traffic accidents, the brain areas affected depend on the type and severity of impact, but due to the mechanics of trauma, the most frequently injured areas are as follows: (1) frontal lobes due to sudden forward motion such as head-on collisions, which may cause deficits in the patient’s decision making, judgement, problem solving, personality, and motor control. (2) Temporal lobes in the sides of the brain during side-impact collisions or rotational injuries, which may affect the patient’s memory, language comprehension, and auditory processing. (3) Occipital lobes in rear-end collisions (head jerks backwards then forwards), which may affect the patient’s visual processing and interpretation. (4) Parietal lobes due to severe or diffuse trauma, which may result in deficits in the patient’s sensory processing and spatial awareness. See Bigler (2001). Our findings in Case 1 have provided affected areas in these lobes by the RTA trauma.

In Case 2, a blast-related mTBI subject has been tested. For this type of mTBI, Taber et al. (2006) found that the affected brain regions might differ slightly from civilian TBIs, though there were overlaps. The most common affected regions after exposures to blasts were in the frontal and temporal areas. In our delta band analysis, the FLR-HC revealed the affected areas: precuneus, superiorparietal, and superiorparietal in the parietal lobe; bankssts and insula in the temporal lobe; parsorbitalis in the frontal lobe; and lingual in the occipital lobe. In the gamma-band analysis, the FLR-HC revealed area rostralmiddlefrontal which generated elevated gamma waves in the frontal lobe. These findings were in agreement with Huang et al. (2021) in their group study of combat-mTBIs. McInnes et al. (2017) pointed out that combat-mTBIs with persistent post-concussive symptoms typically had problems in attention, memory, and other executive functioning. Our study has suggested that they can be due to these particular impaired areas in the four brain lobes.

In Case 3, a sports-related mTBI has been tested. In the sports-related mTBI, the injuries often resulted from repetitive head impacts, rotational forces, and occasional direct blows. In a postmortem study, McKee et al. (2013) showed that the sports-related mTBI would have damages in frontal and temporal lobes, corpus callosum, and hippocampus. In our single-subject studies, we have shown that the frontal, parietal, occipital, limbic, and temporal lobes are reported by the FLR-HC to have abnormal oscillations in either the delta or gamma band.

In Table 4, we have compared the findings of the FLR-HC and PAD-HC in real data analysis. In general, the FLR-HC can reveal more abnormal areas than did the PAD-HC. This is consistent with the simulation studies in the previous section. Interestingly, we have shown that Case 1 shares the abnormal area 27 in the limbic lobe with Case 3, and the abnormal area 14 in the occipital lobe with Case 2. As a whole, these mTBI subjects did share common abnormal lobes but not areas inside the lobes. This implies that diagnosis of mTBI needs to be personalised as done in single-subject studies.

**Table 4.** Highlights of single-mTBI analysis.

		FLR-HC		PAD-HC	
Lobe	Case	Delta	Gamma	Delta	Gamma
Frontal	1	3, 49, 63	6, 28, 40	28, 63	48
	2	54	28	None	None
	3	21, 37	None	None	None
Central S.	1	59	51, 57, 59	17, 59	17, 25, 59
	2	None	None	None	None
	3	None	None	None	None
Parietal	1	8, 32, 42	66	None	28, 30
	2	26, 30, 64	None	30, 60, 64	None
	3	None	42	None	None
Occipital	1	4, 14, 60	12, 14, 48	None	12, 14, 38 48, 56
	2	14	None	None	None
	3	46	None	None	None
Limbic	1	27	2, 11, 24, 27, 36, 45, 61	27	2, 24, 27, 36, 60, 61
	2	None	None	None	2, 36
	3	11, 27, 61	27, 45	11, 36	36
Temporal	1	5, 7, 43, 67	9, 31, 34, 43	27	9
	2	None	None	None	None
	3	1, 11, 27, 61	16, 50, 52	None	None

### 5.5 Generalisability to other data or other imaging modalities

The double-mixture-based likelihood ratio testing procedure with the HC-correction, FLR-HC, and the modified pairwise Anderson–Darling test with the HC-correction, PAD-HC, are potentially extended to other data or other imaging modalities such as electroencephalogram (EEG) and diffusion-weighted magnetic resonance imaging (DWI). For example, the proposed FLR-HC and PAD-HC can be extended to cluster mTBI patients. Aaltonen et al. (2023) pointed out that EEG could provide a cost-effective method for screening purpose in patient groups with a risk for long-term complication, although MEG has superior sensitivity over EEG in source localisation. In clinical neurology, various brain pathologies can be detected by looking at measures of anisotropy and diffusivity. DWI investigates the way that water diffuses within the brain in an applied external magnetic field and can provide information about the integrity of white matter tracts that connect different parts of the brain. For a DWI dataset, the distribution of the fractional anisotropy (FA) was found non-normal in white matter (Muncy et al., 2022). In an ongoing work, we are using the proposed statistical methodology to analyse the DWI data.

### 5.6 Limitations

While our simulation studies have assumed that a sample for each subject has been generated from an inverse MEG imaging, in practical situations these samples are obtained by a preprocessing step: Estimate the source spectrum data from the MEG scan. An important point of future research in this field will account for uncertainty in this preprocessing step when applying the OK test for making a clinical decision. We have focused on tests for differences in marginal distributions between a testing subject and a group of controls. Under this framework, it is difficult to study potential changes in functional connectivity among brain areas. There is a need to extend the proposed methods to the setting of cross-sectional spectral data (Dette & Paparoditis, 2009). The computation of the proposed bootstrapped FLR is time consuming and a parallel computation is required to cope with a very large control group. As pointed out before that a stationarity assumption on sensor time series was required in the current study, it would be very interesting to generalise the current work to the setting of non-stationary sensor data. There is also a need to combine the MEG data with other structure MRI data to further improve the accuracy of diagnosis.

## Supplementary Material

Supplementary Material

## Data Availability

Codes to reproduce simulation results are in https://github.com/zhangjsib/OKtests. A software for source magnitude imaging has been developed by the Innovision IP Ltd for its business and is not publicly available. While the controls in the real data come from the Cambridge Centre for Ageing and Neuroscience (Cam-CAN) dataset (Shafto et al., 2014), the data for the case subject are private.

## Supplementary Materials

The Supplementary Materials are available in https://github.com/zhangjsib/OKtests. In the Supplementary Materials, a pdf file contains extra figures derived from the numerical analysis and real data analysis and *the Desikan-Killiany Atlas* referred in the paper. The Supplementary Materials are organised as follows. A list of brain areas of interest in the Desikan–Killiany Atlas we have referred to is in Section 1. Some extra simulation results for sample size N=150 which we have referred to is in Table 1 and Figure 1, Section 2. Some simulation comparisons of the PAD-HC and the FLR-HC are displayed in Figures 2–3, Section 3. Some extra details in single-subject studies are given in Figures 4–11 for Case 1, Figures 12–18 and Tables 2–3 for Case 2, and Figures 19–25 and Tables 4–5 for Case 3. Supplementary material for this article is available here: https://doi.org/10.1162/IMAG.a.137.

## References

[IMAG.a.137-b1] Aaltonen, J., Heikkinen, V., Kaltiainen, H., Salmelin, R., & Renvall, H. (2023). Sensor-level MEG combined with machine learning yields robust classification of mild traumatic brain injury patients. Clinical Neurophysiology, 153, 79–87. 10.1016/j.clinph.2023.06.010 37459668

[IMAG.a.137-b2] Allen, C. M., Halsey, L., Topcu, G., Rier, L., Gascoyne, L. E., Scadding, J. W., Furlong, P. L., Dunkley, B. T., das Nair, R., Brookes, M. J., & Evangelou, N. (2021). Magnetoencephalography abnormalities in adult mild traumatic brain injury: A systematic review. NeuroImage: Clinical, 31, 102697. 10.1016/j.nicl.2021.102697 34010785 PMC8141472

[IMAG.a.137-b3] Azzalini, A., & Capitanio, A. (2014). The skew-normal and related families. Cambridge University Press. 10.1017/cbo9781139248891

[IMAG.a.137-b4] Benjamini, Y., & Hochberg, Y. (1995). Controlling the false discovery rate: A practical and powerful approach to multiple hypothesis testing. Journal of the Royal Statistical Society Series B, 57(1), 289–300. 10.1111/j.2517-6161.1995.tb02031.x

[IMAG.a.137-b5] Bigler, E. D. (2001). The lesion(s) in traumatic brain injury: Implications for clinical neuropsychology. Archives of Clinical Neuropsychology, 16(2), 95–131. 10.1093/arclin/16.2.95 14590180

[IMAG.a.137-b6] Chen, J., & Li, P. (2009). Hypothesis test for normal mixture models: The EM approach. Annals of Statistics, 37(5A), 2523–2542. 10.1214/08-AOS651

[IMAG.a.137-b7] Chen, J., Li, P., & Fu, Y. (2012). Inference on the order of a normal mixture. Journal of the American Statistical Association, 107(499), 1096–1105. 10.1080/01621459.2012.695668

[IMAG.a.137-b8] Crawford, J. R., & Garthwaite, P. H. (2007). Comparison of a single case to a control or normative sample in neuropsychology: Development of a Bayesian approach. Cognitive Neuropsychology, 24(4), 343–372. 10.1080/02643290701290146 18416496

[IMAG.a.137-b9] Da Costa, L., Robertson, A., Bethune, A., MacDonald, M. J., Shek, P. N., Taylor, M. J., & Pang, E. W. (2015). Delayed and disorganised brain activation detected with magnetoencephalography after mild traumatic brain injury. Journal of Neurology, Neurosurgery and Psychiatry, 86, 1008–1015. 10.1136/jnnp-2014-308571 25324505 PMC4552930

[IMAG.a.137-b10] Dacunha-Castelle, D., & Gassiat, E. (1999). Testing the order of a model using locally conic parametrization: Population mixtures and stationary ARMA processes. Annals of Statistics, 27(4), 1178–1209. 10.1214/aos/1017938921

[IMAG.a.137-b11] Davenport, E. M., Urban, J. E., Vaughan, C., DeSimone, J. C., Wagner, B., Espeland, M. A., Powers, A. K., Whitlow, C. T., Stitzel, J. D., & Maldjian, J. A. (2022). MEG measured delta waves increase in adolescents after concussion. Brain and Behavior, 12(9), e2720. 10.1002/brb3.2720 36053126 PMC9480906

[IMAG.a.137-b12] Desikan, R. S., Ségonne, F., Fischl, B., Quinn, B. T., Dickerson, B. C., Blacker, D., Buckner, R. L., Dale, A. M., Maguire, R. P., Hyman, B. T., Albert, M. S., & Killiany, R. J. (2006). An automated labeling system for subdividing the human cerebral cortex on MRI scans into gyral based regions of interest. NeuroImage, 31(3), 968–980. 10.1016/j.neuroimage.2006.01.021 16530430

[IMAG.a.137-b13] Dette, H., & Paparoditis, E. (2009). Bootstrapping frequency domain tests in multivariate time series with an application to comparing spectral densities. Journal of the Royal Statistical Society: Series B, 71(4), 831–857. 10.1111/j.1467-9868.2009.00709.x

[IMAG.a.137-b14] Dowd, C. (2023). Twosamples: Fast permutation based two sample tests. https://twosampletest.com

[IMAG.a.137-b15] Frost, R., Farrer, T., Primosch, M., & Hedges, D. (2013). Prevalence of traumatic brain injury in the general adult population: A meta-analysis. Neuroepidemiology, 40(3), 154–159. 10.1159/000343275 23257914

[IMAG.a.137-b16] Huang, M.-X., Angeles-Quinto, A., Robb-Swan, A., De-la-Garza, B. G., Huang, C. W., Cheng, C., Hesselink, J. R., Bigler, E. D., Wilde, E. A., Vaida, F., Troyer, E. A., & Max, J. E. (2023). Assessing pediatric mild traumatic brain injury and its recovery using resting-state magnetoencephalography source magnitude imaging and machine learning. Journal of Neurotrauma, 40(11–12), 1112–1129. 10.1089/neu.2022.0220 36884305 PMC10259613

[IMAG.a.137-b17] Huang, M.-X., Huang, C. W., Harrington, D. L., Nichols, S., Robb-Swan, A., Angeles-Quinto, A., Le, L., Rimmele, C., Drake, A., Song, T., Huang, J. W., Clifford, R., Ji, Z., Cheng, C. K., Lerman, I., Yurgil, K. A., Lee, R. R., & Baker, D. G. (2020). Marked increases in resting-state MEG gamma-band activity in combat-related mild traumatic brain injury. Cerebral Cortex, 30(1), 283–295. 10.1093/cercor/bhz087 31041986

[IMAG.a.137-b18] Huang, M.-X., Huang, C. W., Harrington, D. L., Robb-Swan, A., Angeles-Quinto, A., Nichols, S., Huang, J. W., Le, L., Rimmele, C., Matthews, S., Drake, A., Song, T., Ji, Z., Cheng, C. K., Shen, Q., Foote, E., Lerman, I., Yurgil, K. A., Hansen, H. B., … Lee, R. R. (2021). Resting-state magnetoencephalography source magnitude imaging with deep-learning neural network for classification of symptomatic combat-related mild traumatic brain injury. Human Brain Mapping, 42(7), 1987–2004. 10.1089/neu.2016.4581 33449442 PMC8046098

[IMAG.a.137-b19] Huang, M.-X., Nichols, S., Baker, D. G., Robb, A., Angeles, A., Yurgil, K. A., Drake, A., Levy, M., Song, T., McLay, R., Theilmann, R. J., Diwakar, M., Risbrough, V. B., Ji, Z., Huang, C. W., Chang, D. G., Harrington, D. L., Muzzatti, L., Canive, J. M., … Lee, R. R. (2014). Single-subject-based whole-brain MEG slow-wave imaging approach for detecting abnormality in patients with mild traumatic brain injury. NeuroImage: Clinical, 5, 109–119. 10.1016/j.nicl.2014.06.004 25009772 PMC4087185

[IMAG.a.137-b20] Huang, M.-X., Nichols, S., Robb, A., Angeles, A., Drake, A., Holland, M., Asmussen, S., D’Andrea, J., Chun, W., Levy, M., Cui, L., Song, T., Baker, D. G., Hammer, P., McLay, R., Theilmann, R. J., Coimbra, R., Diwakar, M., Boyd, C.,… Lee, R. R. (2012). An automatic MEG low-frequency source imaging approach for detecting injuries in mild and moderate TBI patients with blast and non-blast causes. NeuroImage, 61(4), 1067–1082. 10.1016/j.neuroimage.2012.04.029 22542638

[IMAG.a.137-b21] Huang, M. X., Risling, M., & Baker, D. G. (2016). The role of biomarkers and MEG-based imaging markers in the diagnosis of post-traumatic stress disorder and blast-induced mild traumatic brain injury. Psychoneuroendocrinology, 63, 398–409. 10.1016/j.psyneuen.2015.02.008 25769625

[IMAG.a.137-b22] Itälinna, V., Kaltiainen, H., Forss, N., Liljeström, & M., Parkkonen, L. (2023). Using normative modeling and machine learning for detecting mild traumatic brain injury from magnetoencephalography data. PLoS Computational Biology, 19, e1011613. 10.1371/journal.pcbi.1011613 37943963 PMC10662745

[IMAG.a.137-b23] James, S. L., Theadom, A., Ellenbogen, R. G., Bannick, M. S., Montjoy-Venning, W., Lucchesi, L. R., Abbasi, N., Abdulkader, R., Abraha, H. N., Adsuar, J. C., Afarideh, M., Agrawal, S., Ahmadi, A., Ahmed, M. B., Aichour, A. N., Aichour, I., Aichour, M. T. A., Akinyemi, R. O., Akseer, N., … Murray, C. J. L. (2019). Global, regional, and national burden of traumatic brain injury and spinal cord injury, 1990–2016: A systematic analysis for the Global Burden of Disease Study 2016. Lancet Neurology, 18(1), 56–87. 10.1016/S1474-4422(18)30415-0 30497965 PMC6291456

[IMAG.a.137-b24] Kaltiainen, H., Helle, L., Liljeström, M., Renvall, H., & Forss, N. (2018). Theta-band oscillations as an indicator of mild traumatic brain injury. Brain Topography, 31, 1037–1046. 10.1007/s10548-018-0667-2 30097835 PMC6182433

[IMAG.a.137-b25] Kaltiainen, H., Liljeström, M., Helle, L., Salo, A., Hietanen, M., Renvall, H., & Forss, N. (2019). Mild traumatic brain injury affects cognitive processing and modifies oscillatory brain activity during attentional tasks. Journal of Neurotrauma, 36(14), 2222–2232. 10.1089/neu.2018.6306 30896274 PMC6653790

[IMAG.a.137-b26] Keribin, C. (2000). Consistent estimation of the order of mixture models. Sankhyā: The Indian Journal of Statistics, Series A, 62(2), 49–66. 10.1007/978-981-99-6141-2_5

[IMAG.a.137-b27] Knyazev, G. G. (2012). EEG delta oscillations as a correlate of basic homeostatic and motivational processes. Neuroscience & Biobehavioral Reviews, 36(1), 677–695. 10.1016/j.neubiorev.2011.10.002 22020231

[IMAG.a.137-b28] Maharaj, E. A. (2002). Comparison of non-stationary time series in the frequency domain. Computational Statistics & Data Analysis, 40(1), 131–141. 10.1016/S0167-9473(01)00100-1

[IMAG.a.137-b29] McInnes, K., Friesen, C. L., MacKenzie, D. E., Westwood, D. A., & Boe, S. G. (2017). Mild Traumatic Brain Injury (mTBI) and chronic cognitive impairment: A scoping review. PLoS One, 12(6), e0174847. 10.1371/journal.pone.0218423 28399158 PMC5388340

[IMAG.a.137-b30] McKee, A. C., Stein, T. D., Nowinski, C. J., Stern, R. A., Daneshvar, D. H., Alvarez, V. E., Lee, H., Hall, G., Wojtowicz, S. M., Baugh, C. M., Riley, D. O., Caroline A. Kubilus, C. A., Cormier, K. A., Jacobs, M. A., Martin, B. R., Abraham, C. R., Ikezu, T., Reichard, R. R., … Cantu, R. C. (2013). The spectrum of disease in chronic traumatic encephalopathy. Brain, 136(1), 43–64. 10.1093/brain/aws307 23208308 PMC3624697

[IMAG.a.137-b31] Muncy, N., Kimbler, A., Hedges-Muncy, A., McMakin, D., & Mattfeld, A. (2022). General additive models address statistical issues in diffusion MRI: An example with clinically anxious adolescents. NeuroImage: Clinical, 33, 102937. 10.1016/j.nicl.2022.102937 35033812 PMC8762458

[IMAG.a.137-b32] Namkung, H., Kim, S. H., & Sawa, A. (2017). The insula: An underestimated brain area in clinical neuroscience, psychiatry, and neurology. Trends in Neurosciences, 40(4), 200–207. 10.1016/j.tins.2017.02.002 28314446 PMC5538352

[IMAG.a.137-b33] Ntikas, M., Stewart, W., Ietswaart, M., Hunter, A. M., Maas, A. I. R., Menon, D. K., & Wilson, L. (2024). Contrasting characteristics and outcomes of sports-related and non–sports-related traumatic brain injury. JAMA Network Open, 7(1), e2353318. 10.1001/jamanetworkopen.2023.53318 38265796 PMC10809021

[IMAG.a.137-b34] Rizzo, M. L., & Székely, G. J. (2010). Disco analysis: A nonparametric extension of analysis of variance. Annals of Applied Statistics, 4(2), 1034–1055. http://www.jstor.org/stable/29765541

[IMAG.a.137-b35] Saito, T., & Rehmsmeier, M. (2015). The precision-recall plot is more informative than the ROC plot when evaluating binary classifiers on imbalanced datasets. PLoS One, 10(3), e0118432. 10.1371/journal.pone.0118432 25738806 PMC4349800

[IMAG.a.137-b36] Sahler, C. S., & Greenwald, B. D. (2012). Traumatic brain injury in sports: A review. Rehabilitation Research and Practice, 2012, 659652. 10.1155/2012/659652 22848836 PMC3400421

[IMAG.a.137-b37] Sarvas J. (1987). Basic mathematical and electromagnetic concepts of the biomagnetic inverse problem. Physics in Medicine and Biology, 32(1), 11–22. 10.1088/0031-9155/32/1/004 3823129

[IMAG.a.137-b38] Scholz, F. W., & Stephens, M. A. (1987). K-sample Anderson-Darling tests. Journal of the American Statistical Association, 82(399), 918–924. 10.2307/2288805

[IMAG.a.137-b39] Schwartz, E. S., Edgar, J. C., Gaetz, W. C., & Roberts, T. P. (2010). Magnetoencephalography. Pediatric Radiology, 40, 50–58. 10.1007/s00247-009-1451-y 19937237

[IMAG.a.137-b40] Scrucca, L., Fraley, C., Murphy, T. B., & Raftery, A. E. (2023). Model-based clustering, classification, and density estimation using mclust in R. Chapman and Hall/CRC. 10.1201/9781003277965 PMC5096736 27818791

[IMAG.a.137-b41] Shafto, M. A., Tyler, L. K., Dixon, M., Taylor, J. R., Rowe, J. B., Cusack, R., Calder, A. J., Marslen-Wilson, W. D., Duncan, J., Dalgleish, T., Henson, R. N., Brayne, C., & Matthews, F. E. (2014). The Cambridge Centre for Ageing and Neuroscience (Cam-CAN) study protocol: A cross-sectional, lifespan, multidisciplinary examination of healthy cognitive ageing. BMC Neurology, 14, 204. 10.1186/s12883-014-0204-1 25412575 PMC4219118

[IMAG.a.137-b42] Taber, K. H., Warden, D. L., & Hurley, R. A. (2006). Blast-related traumatic brain injury: What is known? Journal of Neuropsychiatry and Clinical Neurosciences, 18(2), 141–145. 10.1176/appi.neuropsych.18.2.141 16720789

[IMAG.a.137-b43] van der Vaart, A. (1998). Asymptotic statistics. Cambridge University Press. 10.1017/cbo9780511802256

[IMAG.a.137-b44] Verdi, S., Marquand, A. F., Schott, J. M., & Cole, J. H. (2021). Beyond the average patient: How neuroimaging models can address heterogeneity in dementia. Brain, 144(10), 2946–2953. 10.1093/brain/awab165 33892488 PMC8634113

[IMAG.a.137-b45] Virtanen, P., Gommers, R., Oliphant, T. E., Haberland, M., Reddy, T., Cournapeau, D., Burovski, E., Peterson, P., Weckesser, W., Bright, J., van der Walt, S. J., Brett, M., Wilson, J., Millman, K. J., Mayorov, N., Nelson, A. R. J., Jones, E., Kern, R., Larson, E., … SciPy 1.0 Contributors. (2020). SciPy 1.0: Fundamental algorithms for scientific computing in Python. Nature Methods, 17, 261–272. 10.1038/s41592-019-0686-2 32015543 PMC7056644

[IMAG.a.137-b46] von Sachs, R. (2020). Nonparametric spectral analysis of multivariate time series. Annual Review of Statistics and Its Application, 7, 361–386. 10.1146/annurev-statistics-031219-041138

[IMAG.a.137-b47] Wichitchan, S., Yao, W., & Yang, G. (2019). Hypothesis testing for finite mixture models. Computational Statistics and Data Analysis, 132, 180–189. 10.1016/j.csda.2018.05.005

[IMAG.a.137-b48] Winkler, A. M., Webster, M. A., Brooks, J. C., Tracey, I., Smith, S. M., & Nichols, T. E. (2016). Non-parametric combination and related permutation tests for neuroimaging. Human Brain Mapping, 37(4), 1486–1511. 10.1002/hbm.23115 26848101 PMC4783210

[IMAG.a.137-b49] Zhang, J., Liu, C., & Green, G. (2014). Source localization with MEG data: A beamforming approach based on covariance thresholding. Biometrics, 70(1), 121–131. 10.1111/biom.12123 24350784

[IMAG.a.137-b50] Zhang, J., & Su, L. (2015). Temporal autocorrelation-based beamforming with MEG neuroimaging data. Journal of the American Statistical Association, 110(512), 1375–1388. 10.1080/01621459.2015.1054488

