# Supplementary material for: Detecting mild traumatic brain injury with MEG scan data: One-vs-K-sample tests
Source: Imaging Neurosci (Camb). 2025 Sep 8;3:IMAG.a.137. doi: 10.1162/IMAG.a.137 (PMC12418114; doi:10.1162/IMAG.a.137)
Supplement: Supplementary Material [file IMAG.a.137_supp.pdf]

Detecting Mild Traumatic Brain Injury with MEG Scan Data: One-vs-K-Sample Tests —Online Supplementary Materials

Jian Zhang
School of Mathematics, Statistics and Actuarial Science
University of Kent, Canterbury, CT2 7NF, UK
j.zhang-79@kent.ac.uk
Gary Green
Innovision IP Ltd
50 Seymour Street, London W1H 7JG, UK
and
Department of Psychology
University of York, York,YO10 5DD, UK
gary.green@york.ac.uk

The Supplementary Materials are organised as follows. In Section 1, we provide a list of brain areas of interest in terms of the Desikan-Killiany Atlas. In Section 2, we presented extra simulation results for sample size $N = 150$. In Section 3, we present the results of the real data analysis by applying the FLR-HC and PAD-HC to the abnormal areas claimed by the FLR and PAD respectively. In Section 4, we presented simulation results on the abilities of the PAD-HC and the FLR-HC in capturing heterogneity in controls and in seperating the case from controls.

1 Brain areas of interest: the Desikan-Killiany Atlas

Index	Region Name
1	ctx-lh-bankssts
2	ctx-lh-caudalanteriorcingulate
3	ctx-lh-caudalmiddlefrontal
4	ctx-lh-cuneus
5	ctx-lh-entorhinal
6	ctx-lh-frontalpole
7	ctx-lh-fusiform
8	ctx-lh-inferiorparietal
9	ctx-lh-inferiortemporal
10	ctx-lh-insula
11	ctx-lh-isthmuscingulate
12	ctx-lh-lateraloccipital
13	ctx-lh-lateralorbitofrontal
14	ctx-lh-lingual
15	ctx-lh-medialorbitofrontal
16	ctx-lh-middletemporal
17	ctx-lh-paracentral
18	ctx-lh-parahippocampal
19	ctx-lh-parsopercularis
20	ctx-lh-parsorbitalis
21	ctx-lh-parstriangularis
22	ctx-lh-pericalcarine
23	ctx-lh-postcentral
24	ctx-lh-posteriorcingulate
25	ctx-lh-precentral
26	ctx-lh-precuneus
27	ctx-lh-rostralanteriorcingulate
28	ctx-lh-rostralmiddlefrontal
29	ctx-lh-superiorfrontal
30	ctx-lh-superiorparietal

31 ctx-lh-superiortemporal

32 ctx-lh-supramarginal

33 ctx-lh-temporalpole

34 ctx-lh-transversetemporal

35 ctx-rh-bankssts

36 ctx-rh-caudalanteriorcingulate

37 ctx-rh-caudalmiddlefrontal

38 ctx-rh-cuneus

39 ctx-rh-entorhinal

40 ctx-rh-frontalpole

41 ctx-rh-fusiform

42 ctx-rh-inferiorparietal

43 ctx-rh-inferiortemporal

44 ctx-rh-insula

45 ctx-rh-isthmuscingulate

46 ctx-rh-lateraloccipital

47 ctx-rh-lateralorbitofrontal

48 ctx-rh-lingual

49 ctx-rh-medialorbitofrontal

50 ctx-rh-middletemporal

51 ctx-rh-paracentral

52 ctx-rh-parahippocampal

53 ctx-rh-parsopercularis

54 ctx-rh-parsorbitalis

55 ctx-rh-parstriangularis

56 ctx-rh-pericalcarine

57 ctx-rh-postcentral

58 ctx-rh-posteriorcingulate

59 ctx-rh-precentral

60 ctx-rh-precuneus

61 ctx-rh-rostralanteriorcingulate

62 ctx-rh-rostralmiddlefrontal

63 `ctx-rh-superiorfrontal`

64 `ctx-rh-superiorparietal`

65 `ctx-rh-superiortemporal`

66 `ctx-rh-supramarginal`

67 `ctx-rh-temporalpole`

68 `ctx-rh-transversetemporal`

2 Simulation results

Table 1 and Figure 1 show simulated results for the sample size $N = 150$.

[Put Table 1 and Figure 1 here.]

3 Comparison of PAD-HC and FLR-HC

In Figures 2 and 3, we demonstrate the abilities of the PAD-HC and the FLR-HC in separating the case from heterogeneous controls by using simulated Settings 1.1, 1.2 and 1.3, where there are two subgroups in controls. It is clearly shown that the FLR is more capable than the PAD to capture heterogeneity in controls and thus to distinguish the case from the controls.

[Put Figures 2 and 3 here.]

4 Results on real single-subject studies

In the following subsections, we presented some additional numerical results derived from the selected three single-subject studies.

4.1 Case 1

In the following, in addition to Figures 8 to 10 in the main text, we present the dendrograms for the abnormal areas claimed by the FLR and PAD in the delta- and gamma-band respectively. We

Table 1: Percentages of instances with p-value less than 0.05 and metrics: $N = 150$.

Setting/Metrics	Method					
	FLR	CFLR	PAD	CPAD	PMAD	ADM
1.1	0.06	0.2	0.06	0.16	1	0.02
1.2	0	0	0.04	0	0	0.08
1.3	0.42	0.32	0.46	0.56	0.04	0
1.4	0.92	0.86	0.44	0.56	0.26	0.1
1.5	1	1	1	1	1	1
Precision	**0.98**	0.92	0.97	0.93	0.57	0.92
Recall	**0.78**	0.73	0.63	0.71	0.43	0.37
F_1	**0.87**	0.81	0.76	0.81	0.49	0.53
$F_{0.5}$	**0.93**	0.72	0.87	0.87	0.53	0.71
F_2	0.81	**0.91**	0.68	0.75	0.45	0.37
2.1	0.12	0	0.06	0.3	0.44	0.28
2.2	0.36	0	0	0.14	0.04	0
2.3	0.98	0.1	0.94	0.96	0.96	0.28
Precision	**0.92**	1	0.94	0.79	0.69	0.50
Recall	**0.67**	0.05	0.47	0.55	0.50	0.14
F_1	**0.78**	0.1	0.63	0.65	0.58	0.22
$F_{0.5}$	**0.86**	0.21	0.78	0.73	0.71	0.33
F_2	**0.71**	0.06	0.52	0.59	0.53	0.16
3.1	0.12	0.06	0	0.04	0	0.02
3.2	1	1	1	1	1	1
3.3	0.8	0.96	0.4	0.96	0.68	0.96
Precision	0.94	0.97	**1**	0.98	1	0.99
Recall	0.82	0.94	0.70	**0.98**	0.72	0.90
F_1	0.88	0.95	0.82	**0.98**	0.91	0.98
$F_{0.5}$	0.91	0.96	0.92	0.98	0.96	**0.99**
F_2	0.84	0.95	0.74	**0.98**	0.74	0.98

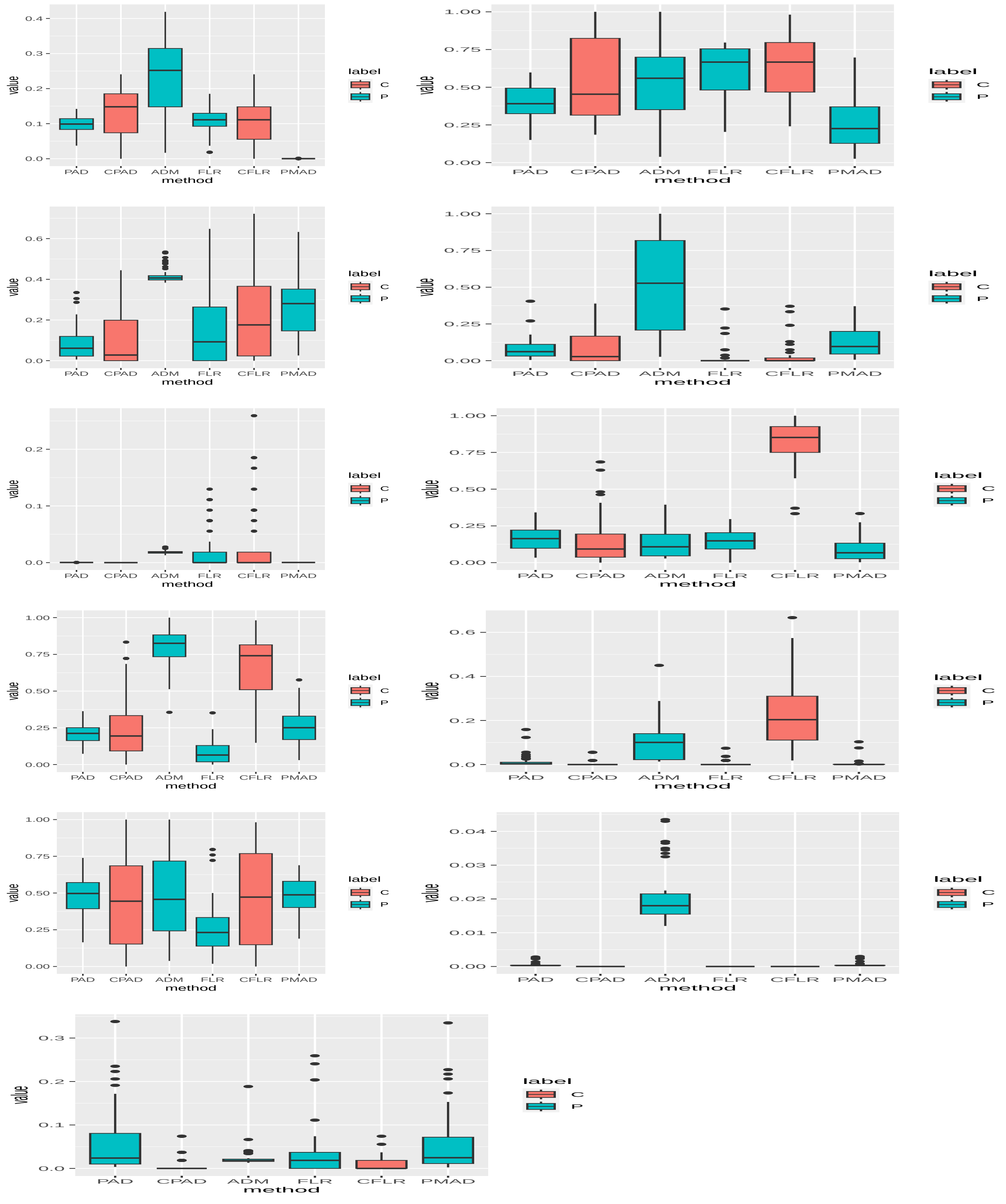

Figure 1: Settings 1, 2.3 for $N = 150$. P-value plots from left to right and from top row to down row respectively. Rows 1 to 5 are corresponding to testing each of 5 cases against the Controls respectively.

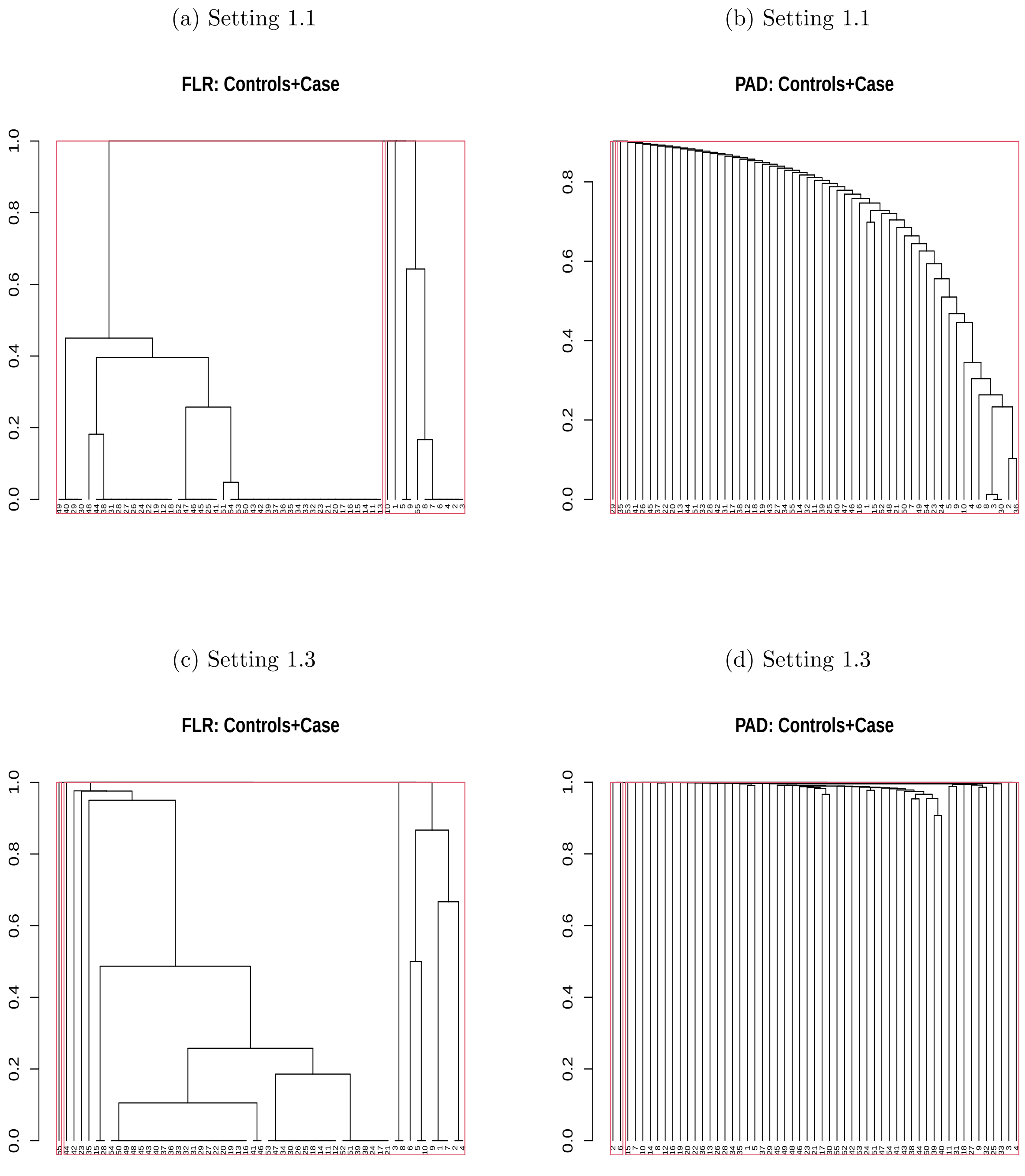

Figure 2: Examples of the FLR-HC dendrograms for the simulated Settings 1.1 and 1.3. Red boxes indicate the cluster borders if we partition 55 subjects into two clusters.

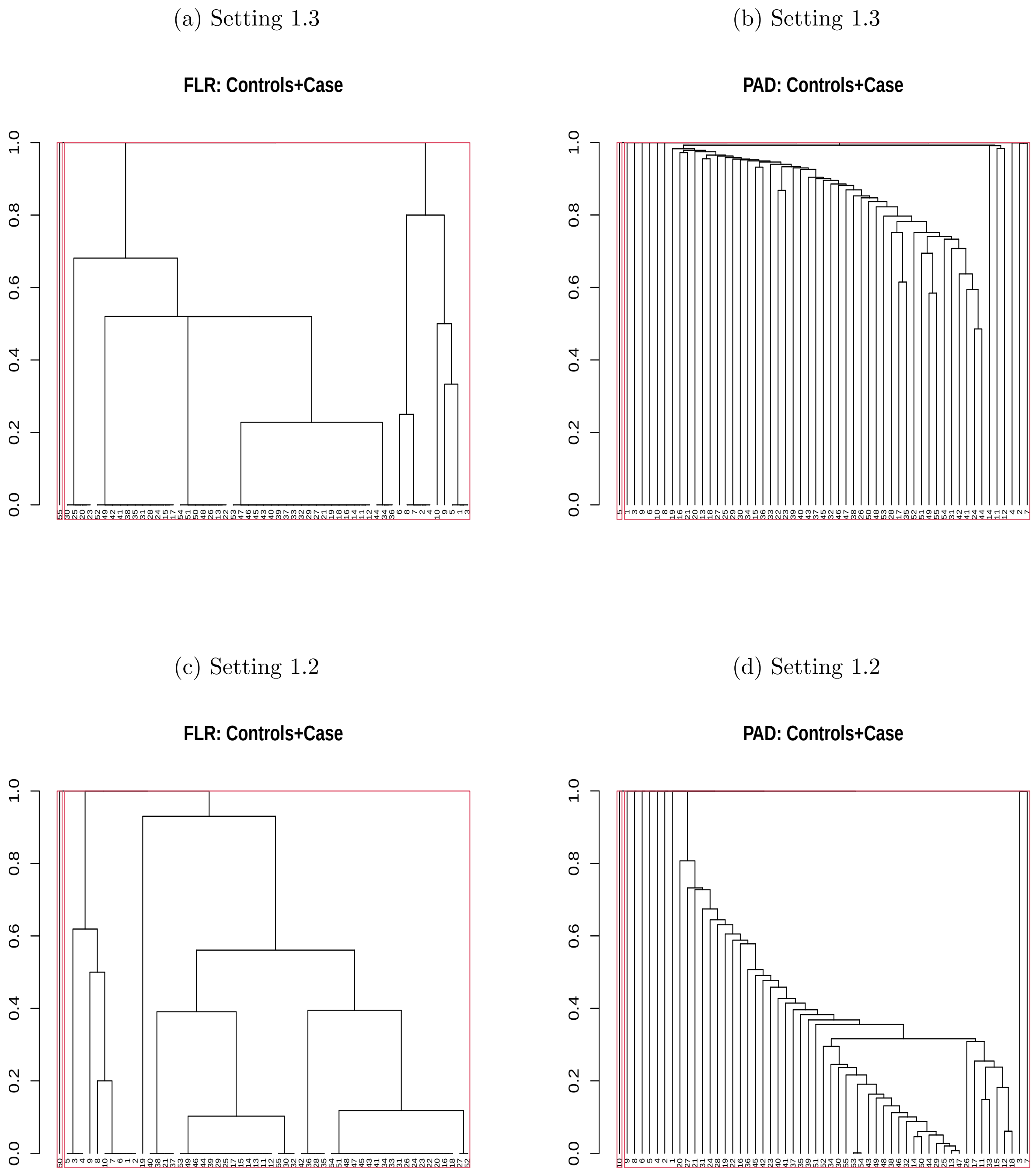

Figure 3: Examples of the FLR-HC dendrograms for the simulated Settings 1.2 and 1.3. Red boxes indicate the cluster borders if we partition 55 subjects into two clusters.

select these areas, where the case-subject 55 is the last one to be merged into the dendrogram, as the FLR-HC and PAD-HC adjusted areas. See Figures 4∼11 .

[Put Figures 4∼11 here.]

(a) Delta

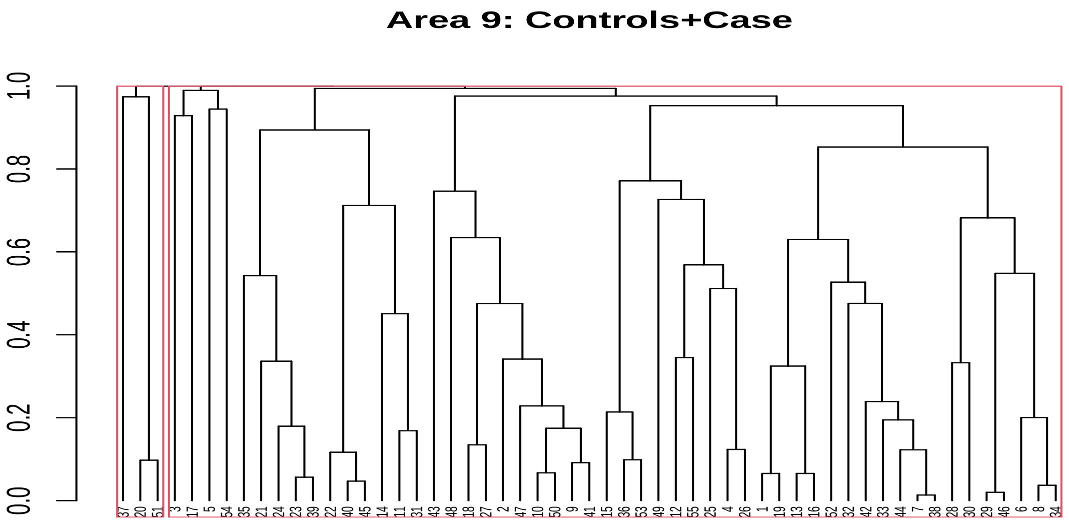

2

(b) Gamma

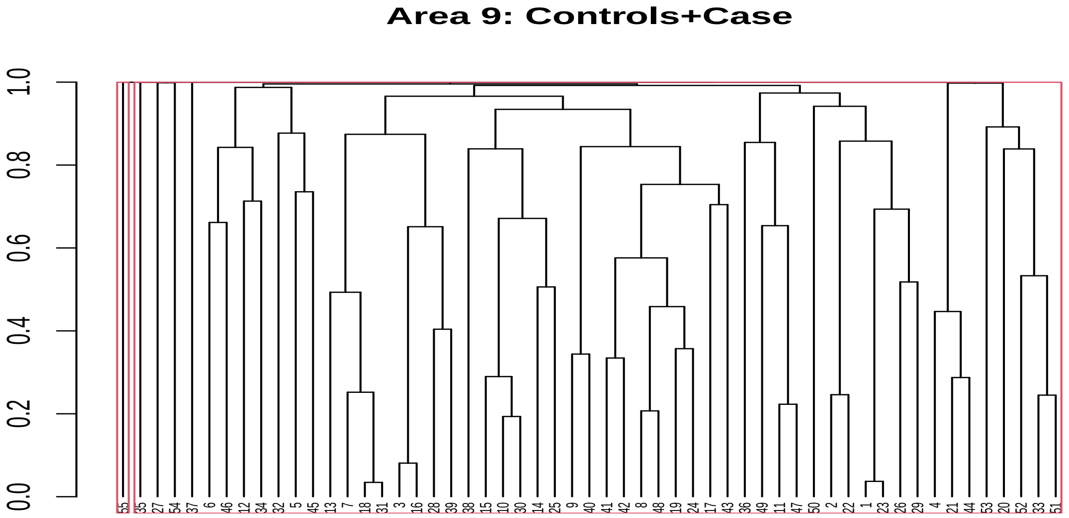

Figure 4: The PAD-HC dendrograms for the area 9 in the delta and gamma bands of Case 1 respectively. The controls are indexed by 1 to 54 while the case is indexed by 55. Red boxes indicate the cluster borders if we partition 55 subjects into two clusters.

(a) Delta

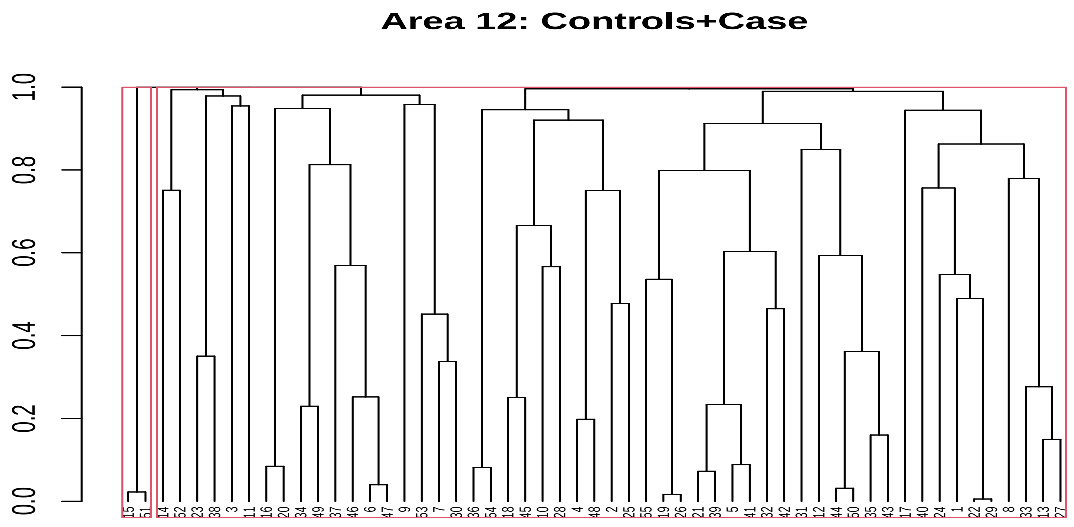

(b) Gamma

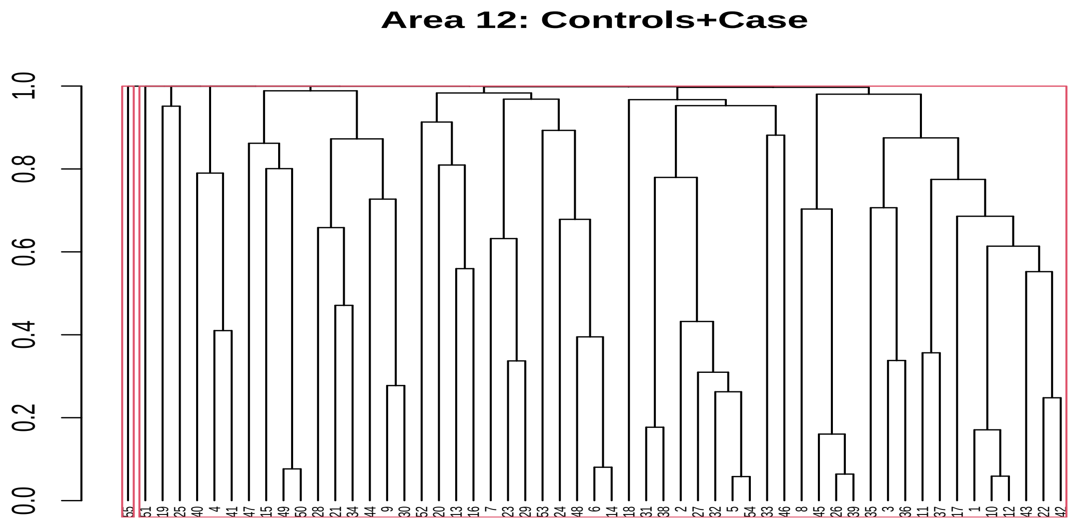

Figure 5: The PAD-HC dendrograms for the area 12 in the delta and gamma bands of Case 1 respectively. The controls are indexed by 1 to 54 while the case is indexed by 55. Red boxes indicate the cluster borders if we partition 55 subjects into two clusters.

(a) Delta

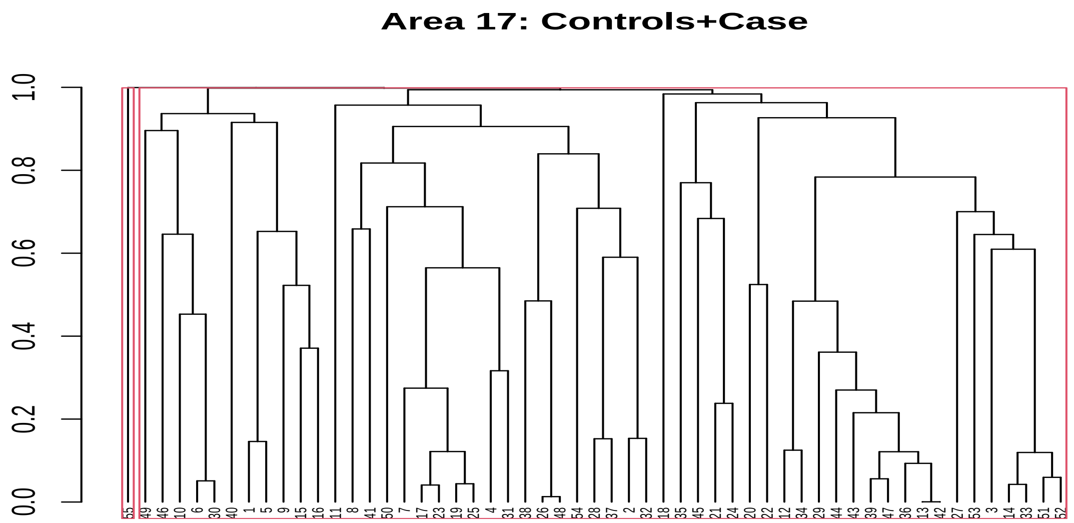

(b) Delta

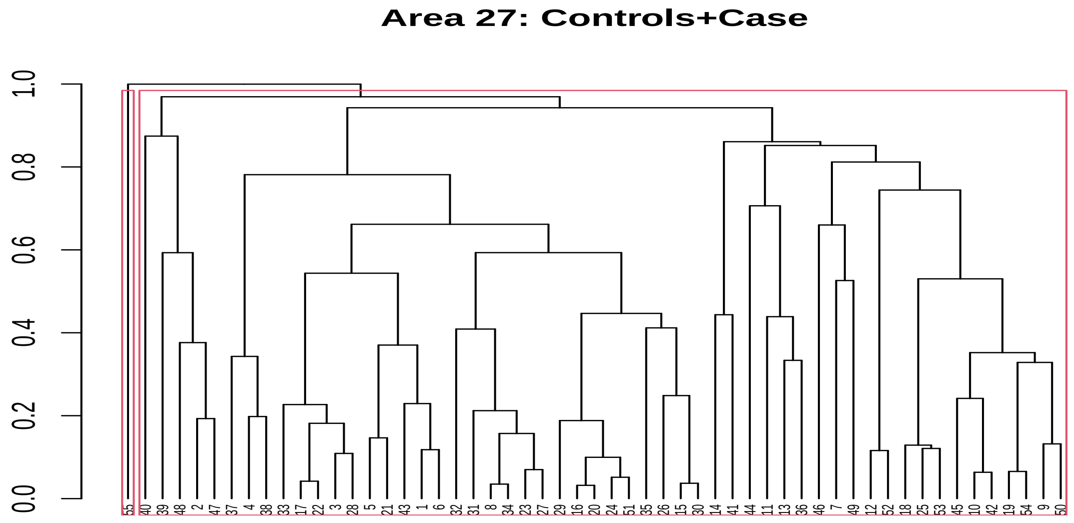

Figure 6: Examples of the PAD-HC dendrograms for the delta- and gamma- band data of Case 1 respectively. Red boxes indicate the cluster borders if we partition 55 subjects into two clusters.

(a) Gamma

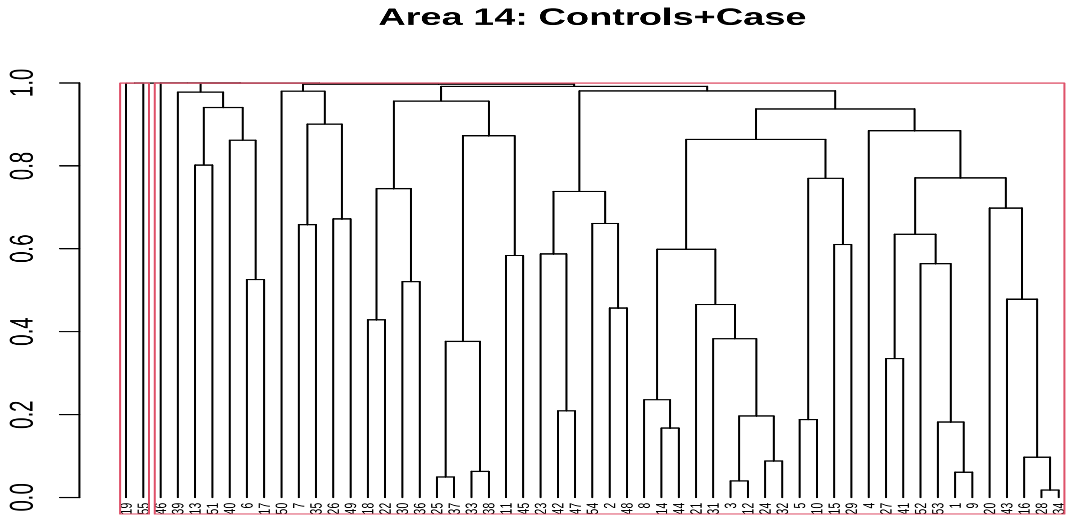

(b) Gamma

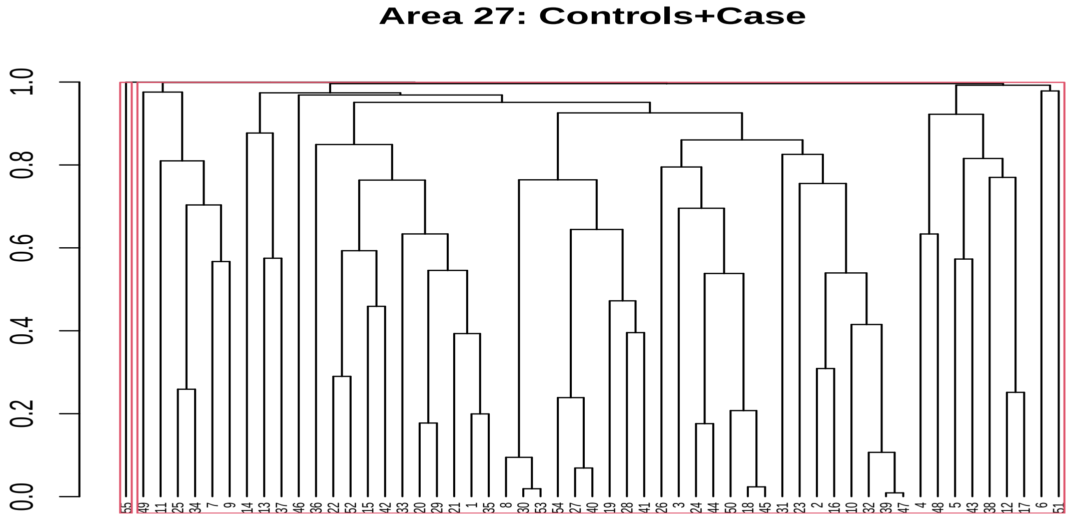

Figure 7: Examples of the PAD-HC dendrograms for the delta- and gamma- band data of Case 1 respectively. Red boxes indicate the cluster borders if we partition 55 subjects into two clusters.

(a) Delta

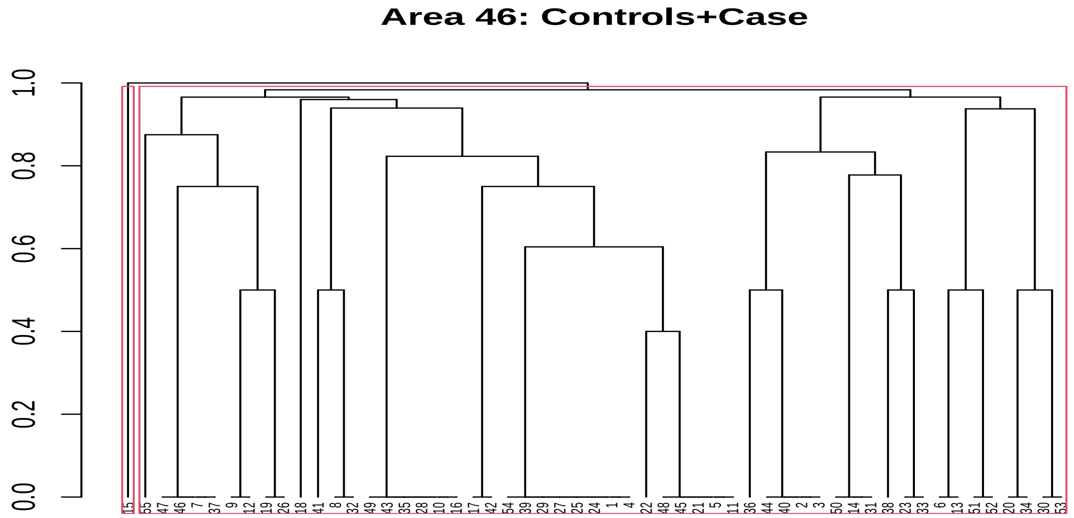

(b) Delta

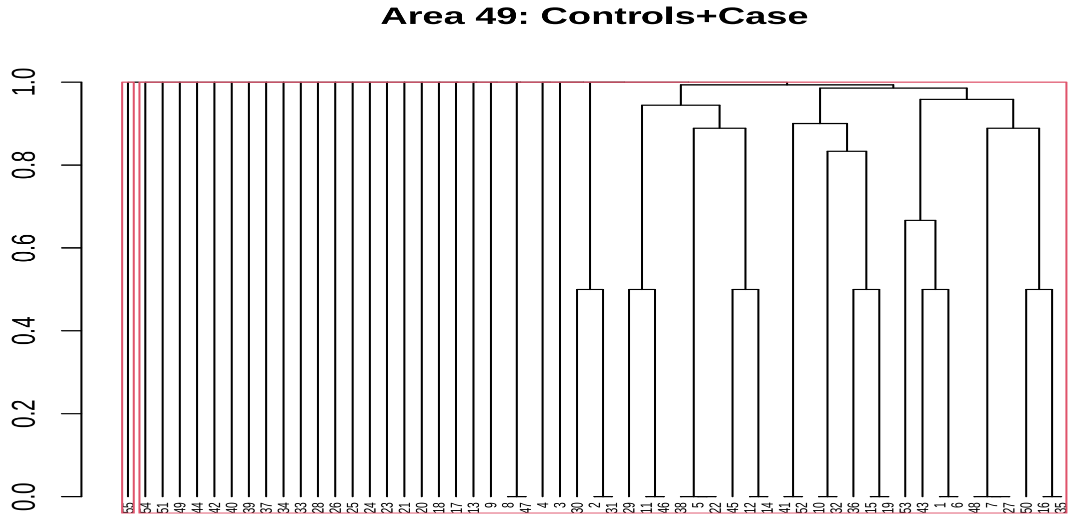

Figure 8: The FLR-HC dendrograms for the areas 46 and 49 in the delta band of Case 1 respectively. Red boxes indicate the cluster borders if we partition 55 subjects into two clusters.

(a) Delta

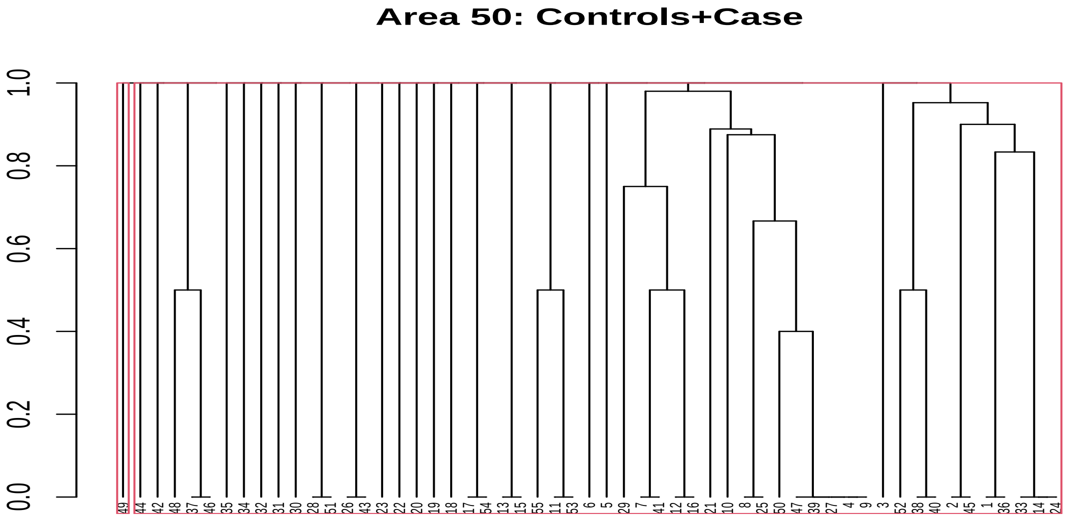

(b) Delta

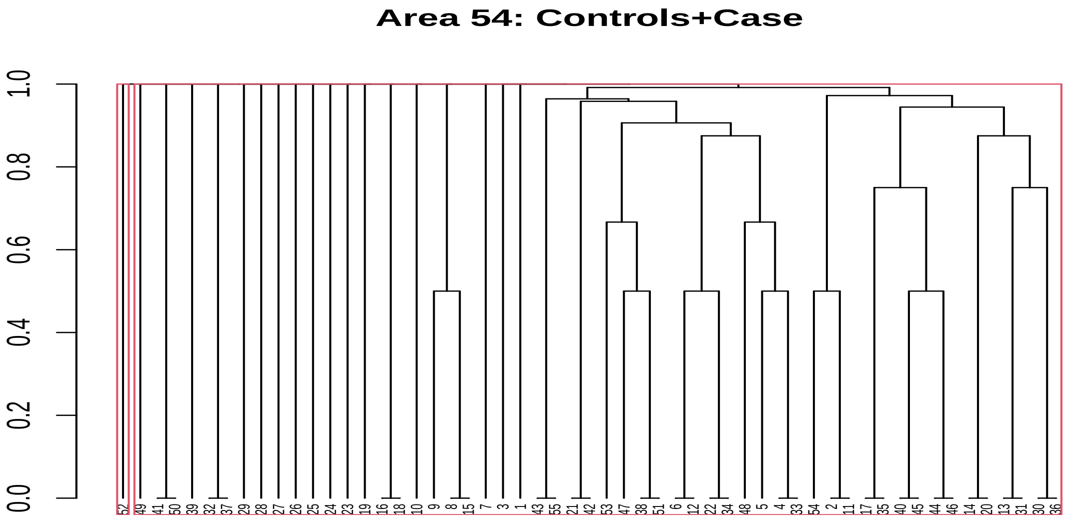

Figure 9: The FLR-HC dendrograms for the areas 50 and 54 in the delta band of Case 1 respectively. Red boxes indicate the cluster borders if we partition 55 subjects into two clusters.

(a) Gamma

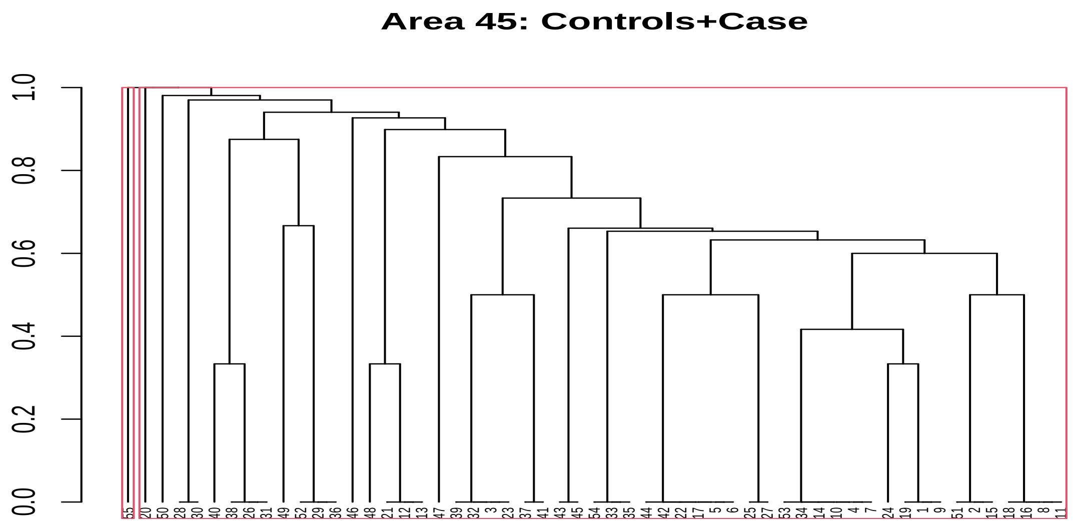

(b) Gamma

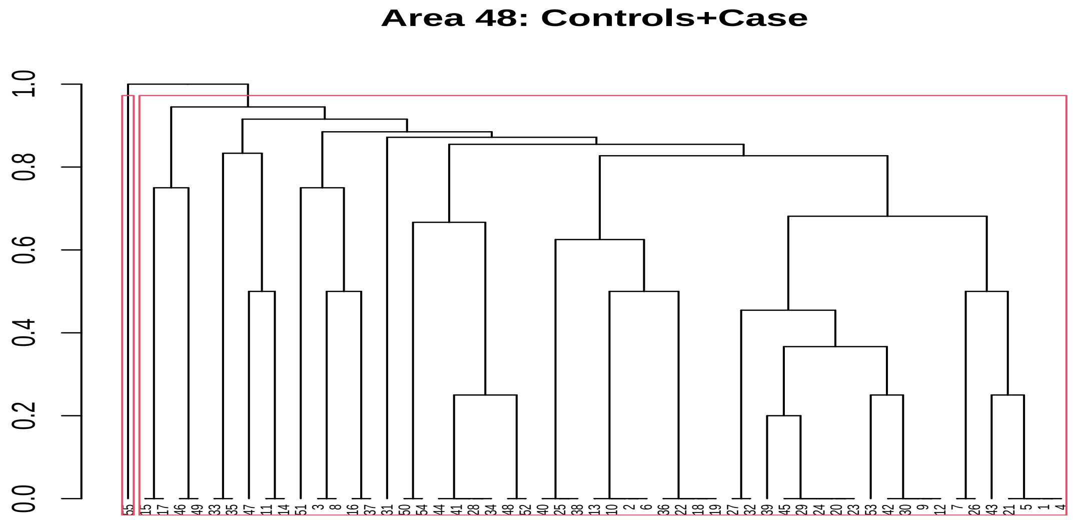

Figure 10: The FLR-HC dendrograms for the areas 45 and 48 in the gamma band of Case 1 respectively. Red boxes indicate the cluster borders if we partition 55 subjects into two clusters.

(a) Gamma

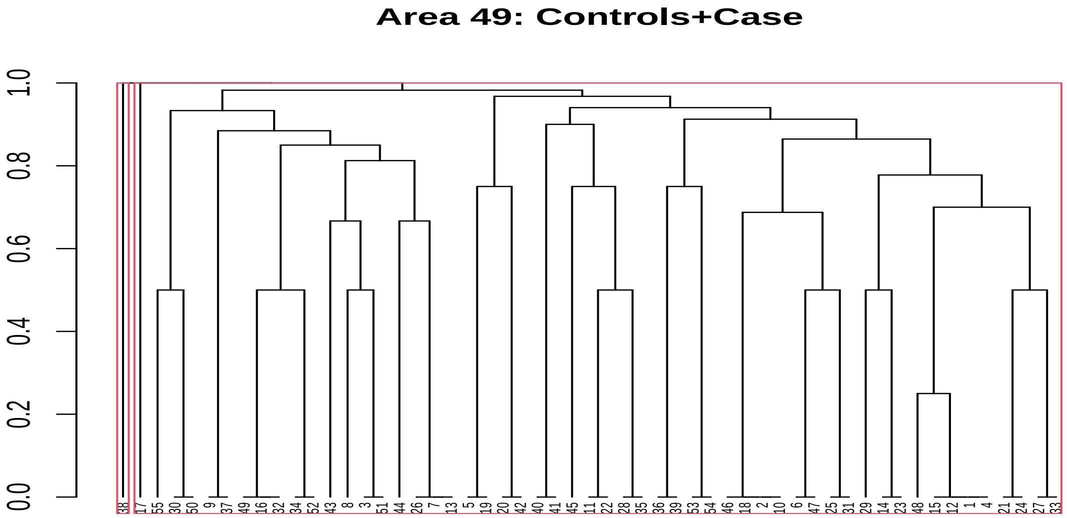

(b) Gamma

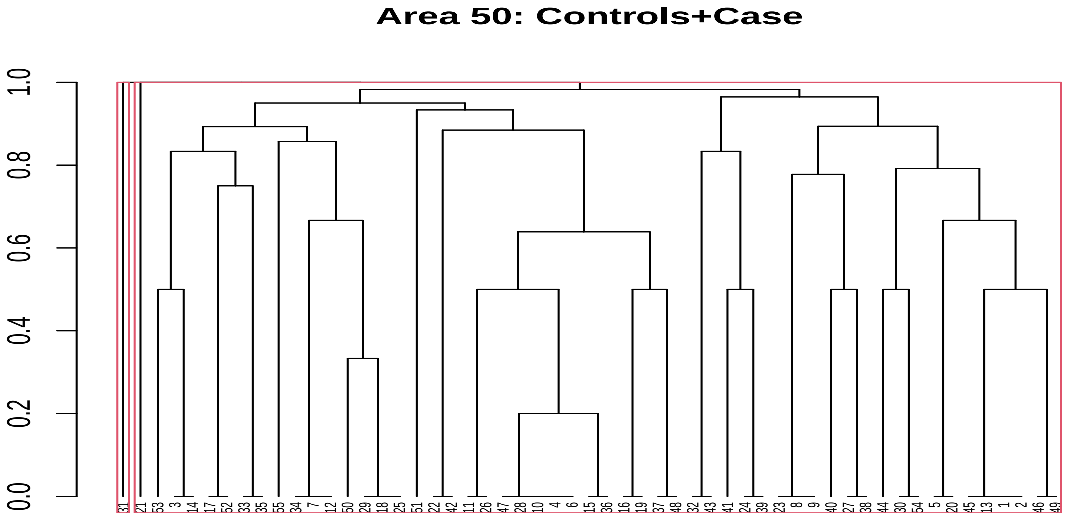

Figure 11: The FLR-HC dendrograms for the areas 49 and 50 in the gamma band of Case 1 respectively. Red boxes indicate the cluster borders if we partition 55 subjects into two clusters.

4.2 Case 2

[Put Tables 2 and 3 here.]

Table 2: The delta band data analysis for mTBI case 2.

Methods	Hemisphere	Areas	Adj.p-values
FLR	lh	12, 14, 20, 25-27, 30, 32, 34	< 0.01
	rh	35, 40, 42, 44, 46, 49, 50, 54, 55, 57, 58, 63-65, 67	< 0.01
FLR-HC	lh	14, 26, 30	< 0.01
	rh	35, 44, 54, 64	< 0.01
CFLR	lh	None	< 0.01
	rh	60	< 0.01
PAD	lh	8, 12, 30	< 0.01
	rh	60, 64	< 0.01
PAD-HC	lh	30	< 0.01
	rh	60, 64	< 0.01
CPAD	lh	27	< 0.01
	rh	60, 64	< 0.01
PMAD	lh	2-5, 8, 9, 11, 12, 16, 18-21, 23, 25-34	< 0.01
	rh	35, 36, 39, 41, 42, 45, 46, 49, 50, 52 , 56, 57, 59-66, 68	< 0.01
ADM	lh	None	< 0.01
	rh	None	< 0.01

[Put Figures 12∼18 here.]

(a) Delta

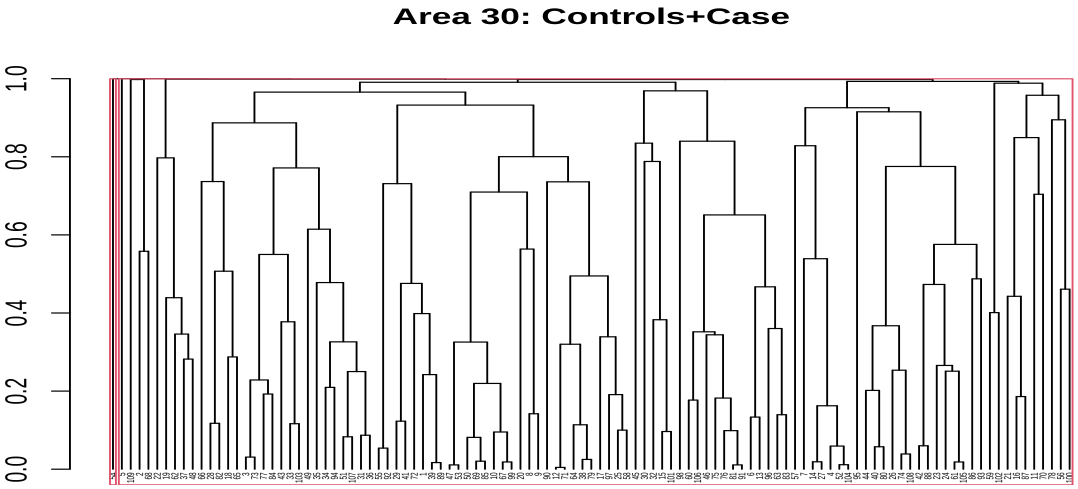

2

(b) Delta

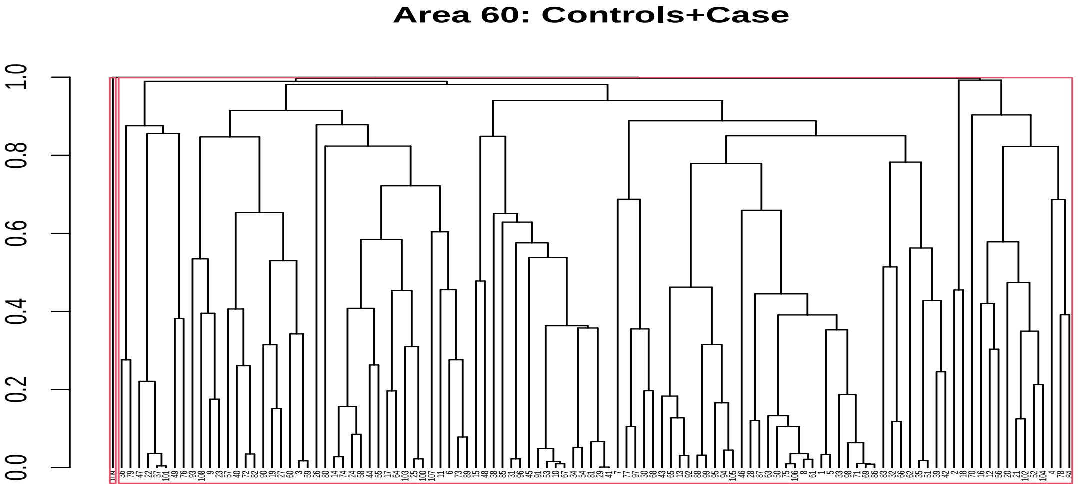

Figure 12: The PAD-HC dendrograms for the areas 30 and 60 in the delta band of Case 2 respectively. The controls are indexed by 1 to 108 while the case is indexed by 109. Red boxes indicate the cluster borders if we partition 109 subjects into two clusters.

Table 3: The gamma band data analysis for mTBI case 2.

Methods	Hemisphere	Areas	Adj.p-values
FLR	lh	2, 7, 12-14, 19, 28, 29, 31, 33	< 0.01
	rh	40, 46-48, 50, 51, 53, 61, 63, 68	< 0.01
FLR-HC	lh	28	< 0.01
	rh	None	< 0.01
CFLR	lh	None	< 0.01
	rh	36, 53, 54, 55	< 0.01
PAD	lh	2, 29	< 0.01
	rh	36	< 0.01
PAD-HC	lh	2	< 0.01
	rh	36	< 0.01
CPAD	lh	None	< 0.01
	rh	36	< 0.01
PMAD	lh	1-16, 18,19, 21-23, 25, 28-34	< 0.01
	rh	35-39, 41-48, 51-57, 59-60, 62-68	< 0.01
ADM	lh	None	< 0.01
	rh	None	< 0.01

(a) Delta

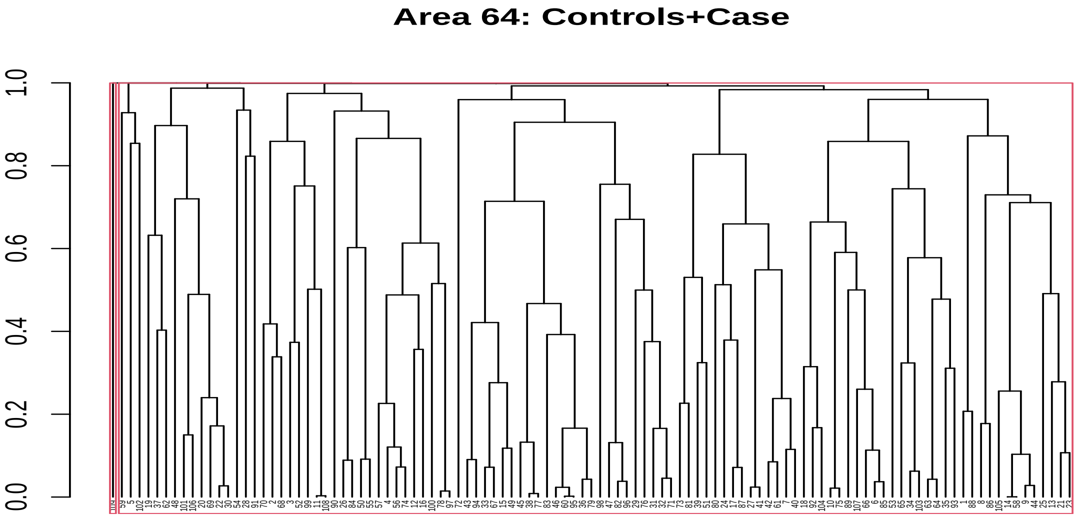

Figure 13: The PAD-HC dendrogram for area 64 in the delta band of Case 2. The controls are indexed by 1 to 108 while the case is indexed by 109. Red boxes indicate the cluster borders if we partition 109 subjects into two clusters.

(a) Gamma

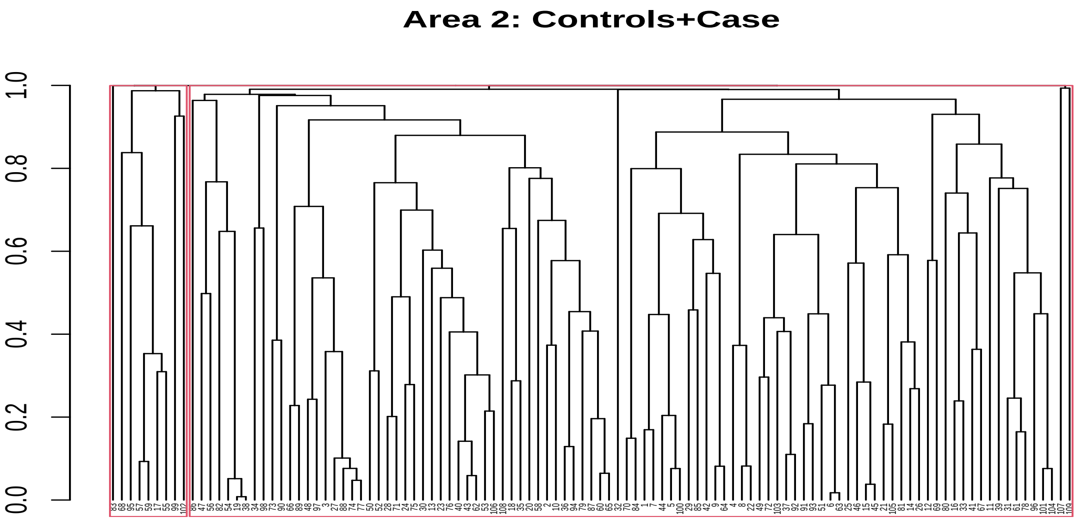

(b) Gamma

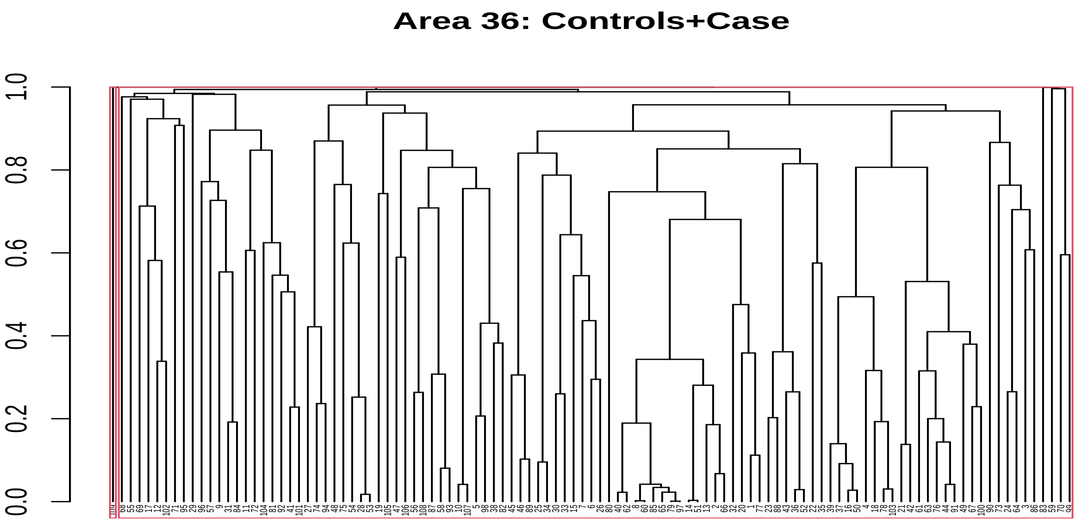

Figure 14: The PAD-HC dendrograms for the areas 2 and 36 in the gamma band of Case 2 respectively. Red boxes indicate the cluster borders if we partition 109 subjects into two clusters.

(a) Delta

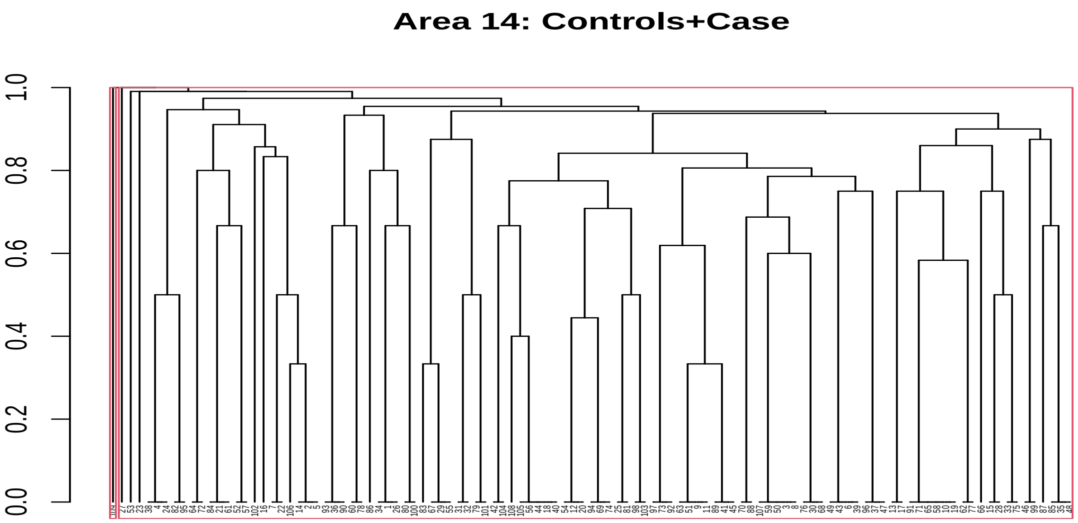

(b) Delta

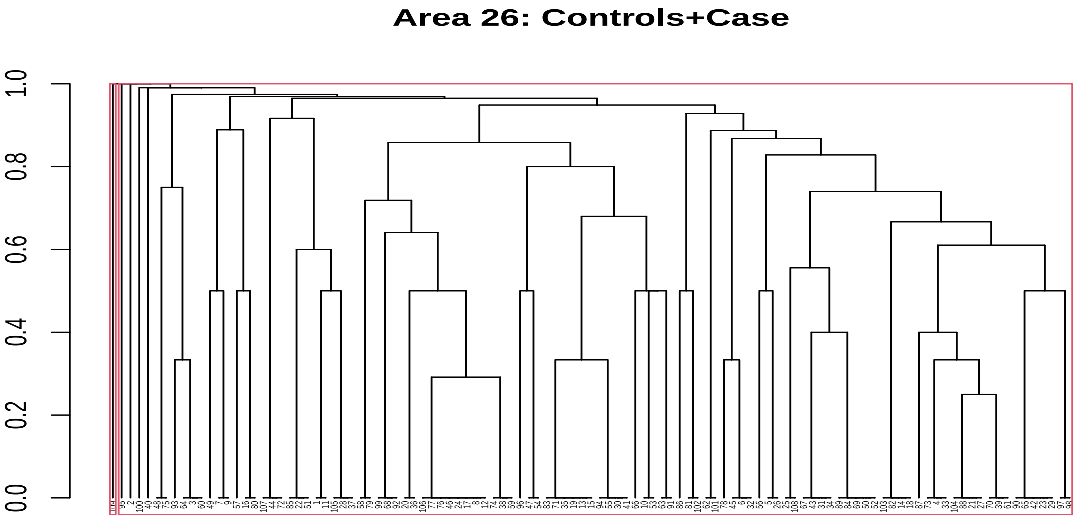

Figure 15: The FLR-HC dendrograms for the areas 14 and 26 in the delta band of Case 2 respectively. Red boxes indicate the cluster borders if we partition 109 subjects into two clusters.

(a) Delta

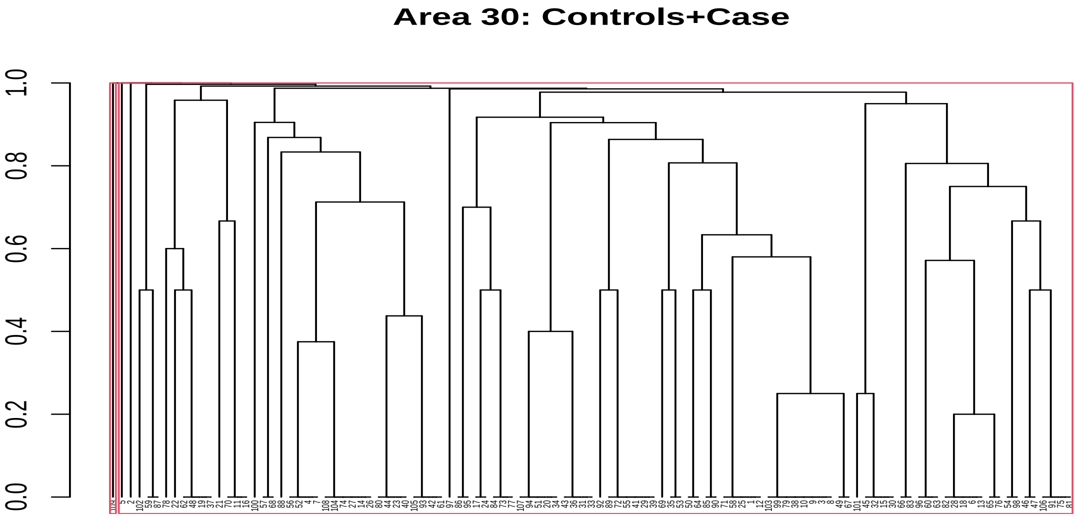

(b) Delta

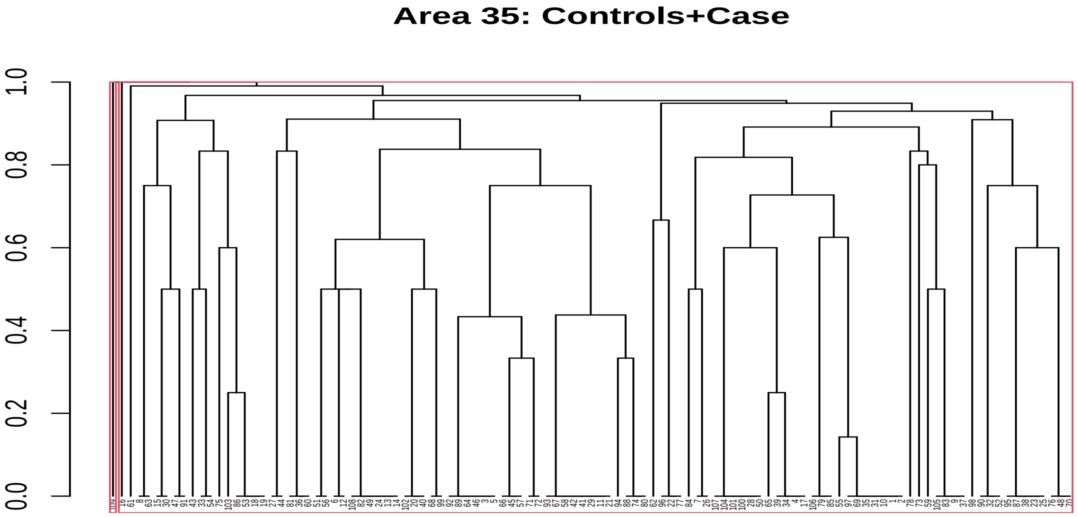

Figure 16: The FLR-HC dendrograms for the areas 30 and 35 in the delta band of Case 2 respectively. Red boxes indicate the cluster borders if we partition 109 subjects into two clusters.

(a) Delta

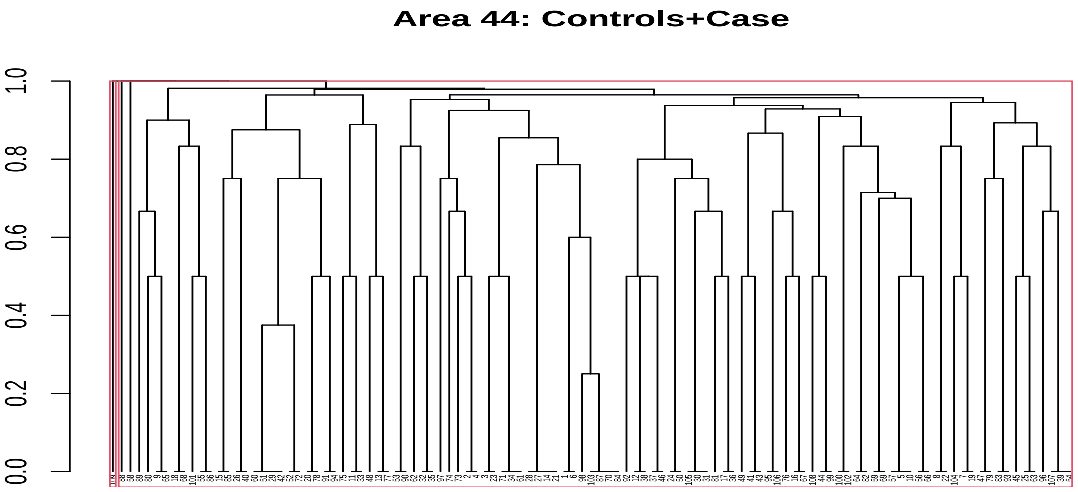

(b) Delta

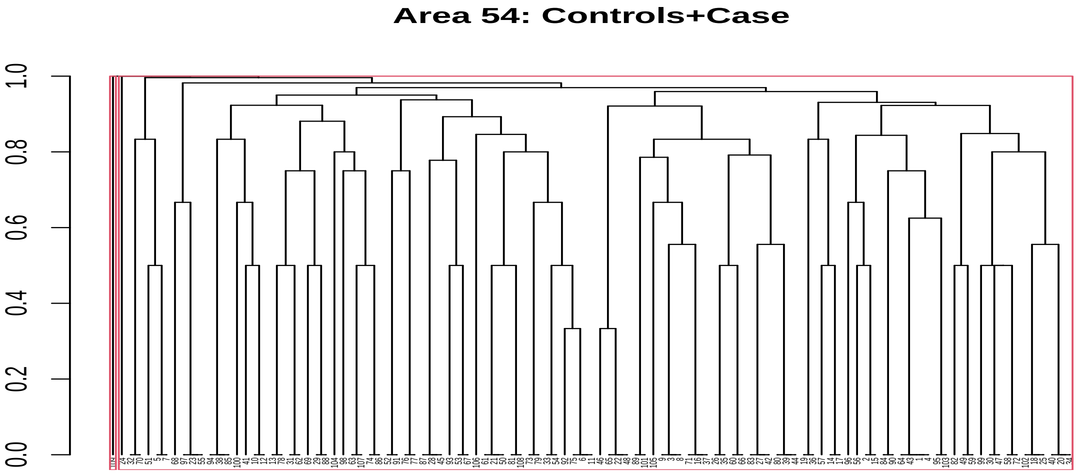

Figure 17: The FLR-HC dendrograms for the areas 44 and 54 in the delta band of Case 2 respectively. Red boxes indicate the cluster borders if we partition 109 subjects into two clusters.

(a) Delta

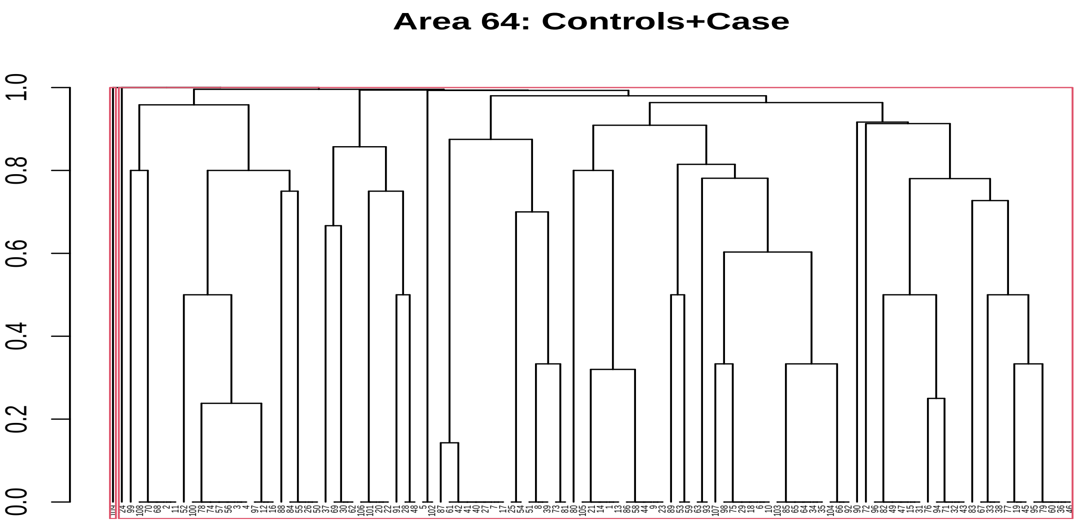

(b) Gamma

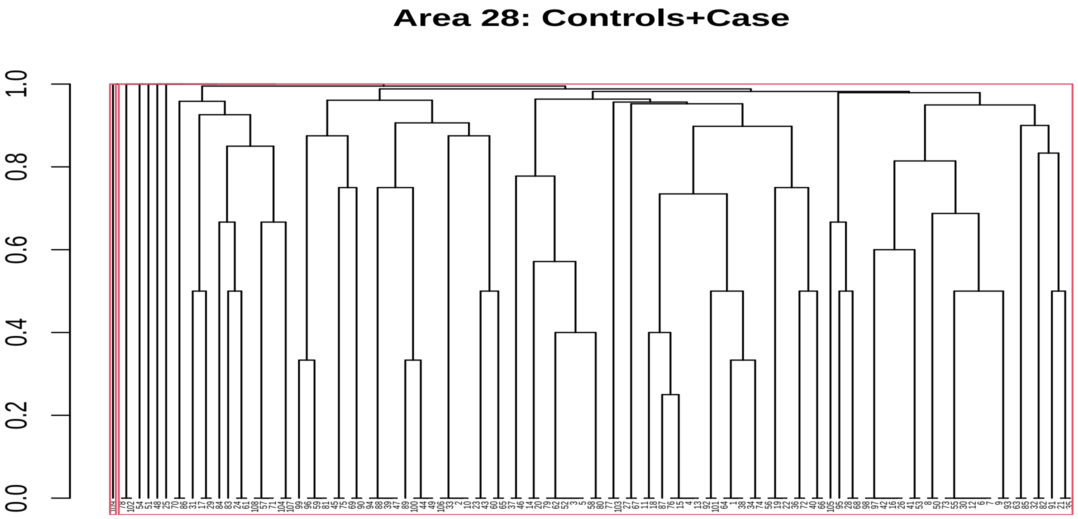

Figure 18: The FLR-HC dendrograms for the areas 64 and 28 in the delta and gamma bands of Case 2 respectively. Red boxes indicate the cluster borders if we partition 109 subjects into two clusters.

4.3 Case 3

[Put Tables 4 and 5 here.]

Table 4: The delta band data analysis for mTBI case 3.

Methods	Hemisphere	Areas	Adj.p-values
FLR	lh	1, 2, 6, 11, 12, 17, 18, 21-23, 26, 27, 29, 34	< 0.01
	rh	37, 40, 46, 56, 61, 64, 65, 68	< 0.01
FLR-HC	lh	1, 11, 21, 27	< 0.01
	rh	37, 46, 61	< 0.01
CFLR	lh	1, 17, 27	< 0.01
	rh	None	< 0.01
PAD	lh	None	< 0.01
	rh	None	< 0.01
PAD-HC	lh	None	< 0.01
	rh	None	< 0.01
CPAD	lh	None	< 0.01
	rh	None	< 0.01
PMAD	lh	1-14, 16-26, 28-32, 34, 36-38	< 0.01
	rh	36-38, 42-47, 50-53, 55-62, 63-66, 68	< 0.01
ADM	lh	None	< 0.01
	rh	None	< 0.01

[Put Figures 19∼25 here.]

(a) Delta

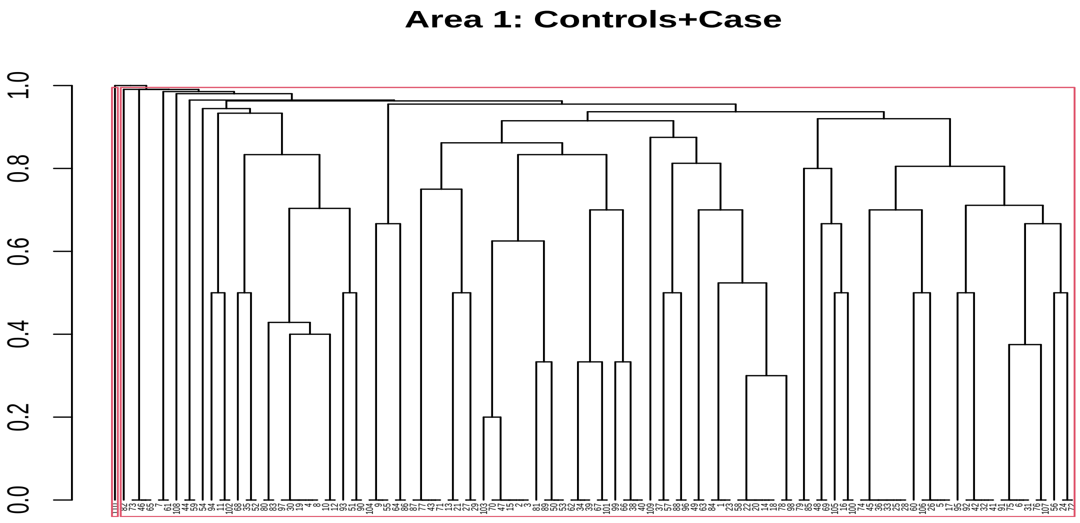

(b) Delta

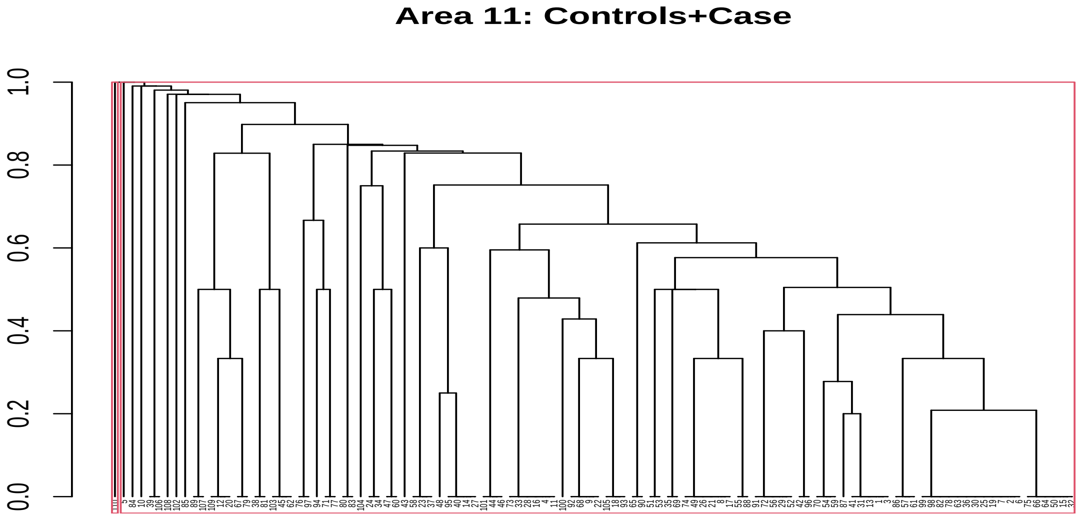

Figure 19: The FLR-HC dendrograms for the areas 1 and 11 in the delta-band of Case 3 respectively. Red boxes indicate the cluster borders if we partition 110 subjects into two clusters.

Table 5: The gamma band data analysis for mTBI case 3.

Methods	Hemisphere	Areas	Adj.p-values
FLR	lh	6, 10, 13, 16, 25, 27, 28, 31, 32	< 0.01
	rh	37-39, 42, 45, 48, 50, 52, 55, 61, 64, 65, 68	< 0.01
FLR-HC	lh	16, 27	< 0.01
	rh	42, 45, 50, 52	< 0.01
CFLR	lh	28	< 0.01
	rh	42, 50	< 0.01
PAD	lh	None	< 0.01
	rh	None	< 0.01
PAD-HC	lh	None	< 0.01
	rh	None	< 0.01
CPAD	lh	None	< 0.01
	rh	None	< 0.01
PMAD	lh	1-26, 28-34	< 0.01
	rh	36-39, 41-48, 50, 52-57, 59, 60, 62-68	< 0.01
ADM	lh	None	< 0.01
	rh	None	< 0.01

(a) Delta

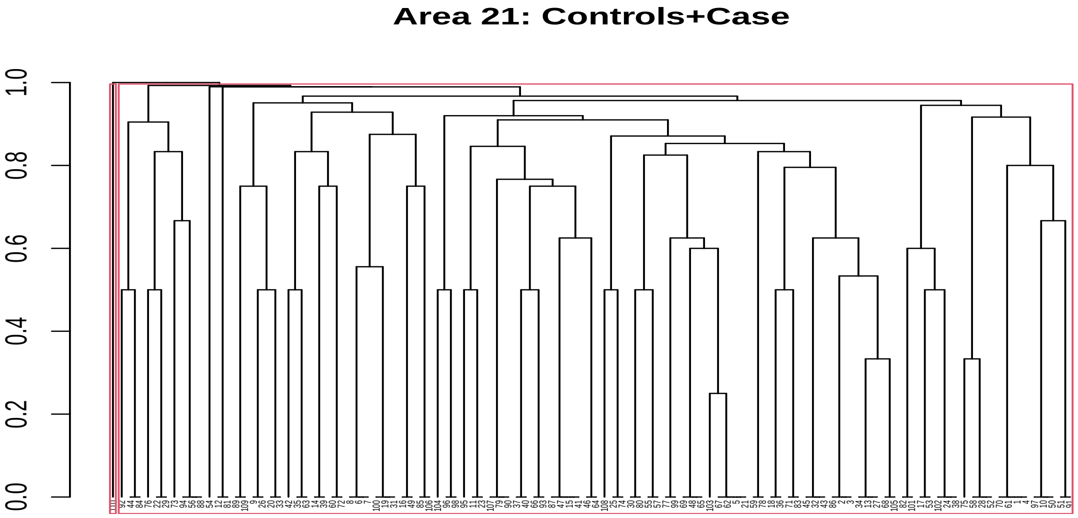

(b) Delta

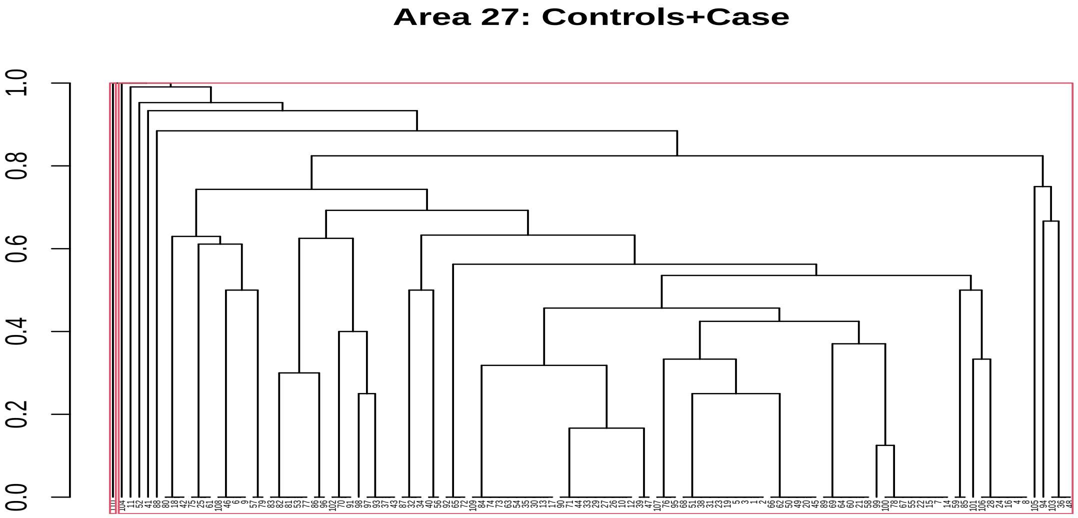

Figure 20: The FLR-HC dendrograms for the areas 21 and 27 in the delta band of Case 3 respectively. Red boxes indicate the cluster borders if we partition 110 subjects into two clusters.

(a) Delta

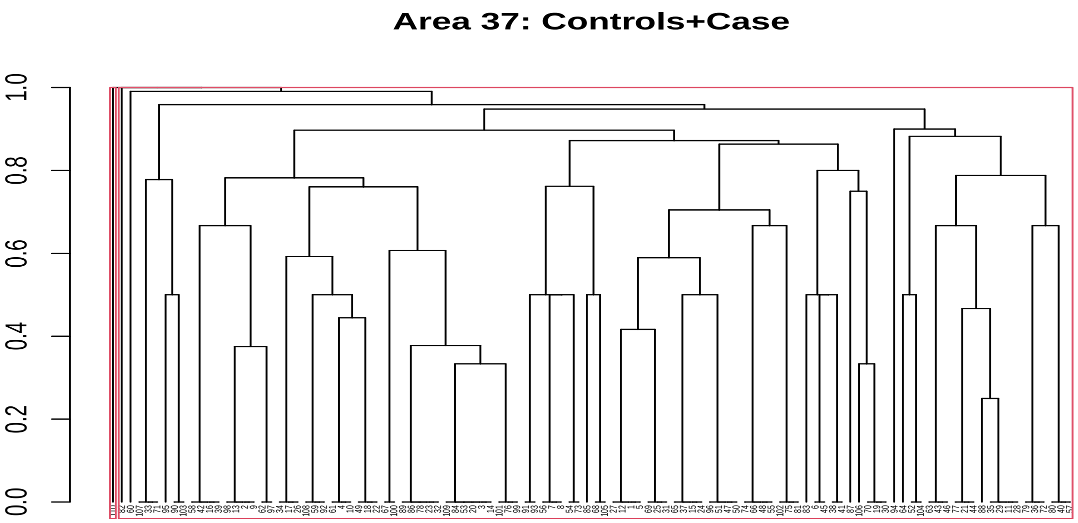

(b) Delta

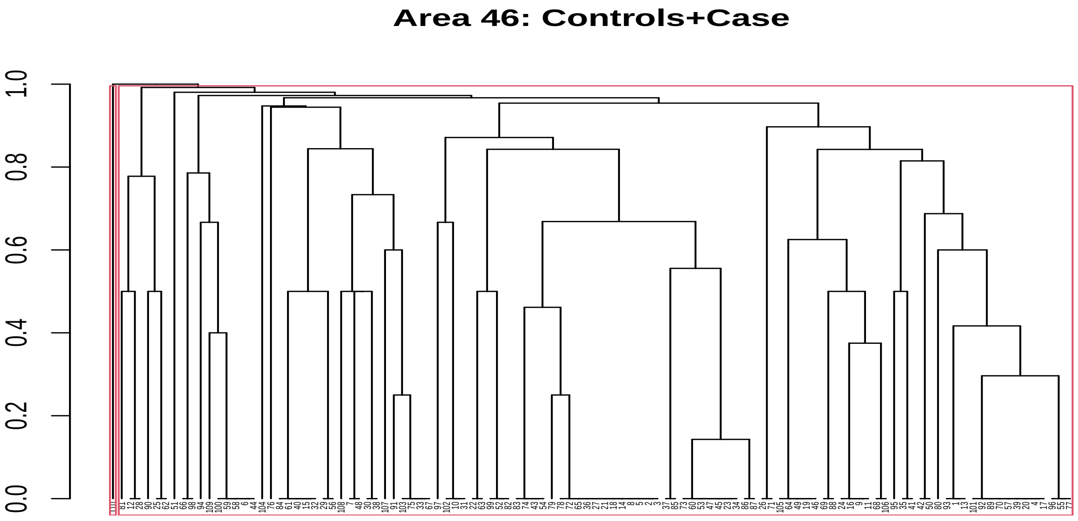

Figure 21: The FLR-HC dendrograms for the areas 37 and 46 in the delta band of Case 3 respectively. Red boxes indicate the cluster borders if we partition 110 subjects into two clusters.

(a) Delta

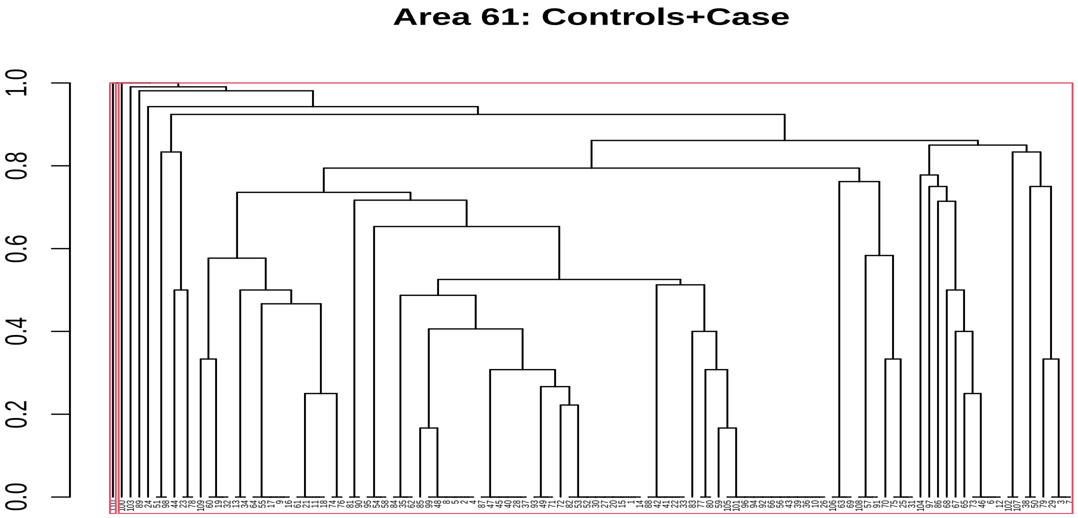

Figure 22: The FLR-HC dendrogram for area 61 in the delta band of Case 3. Red boxes indicate the cluster borders if we partition 110 subjects into two clusters.

(a) Gamma

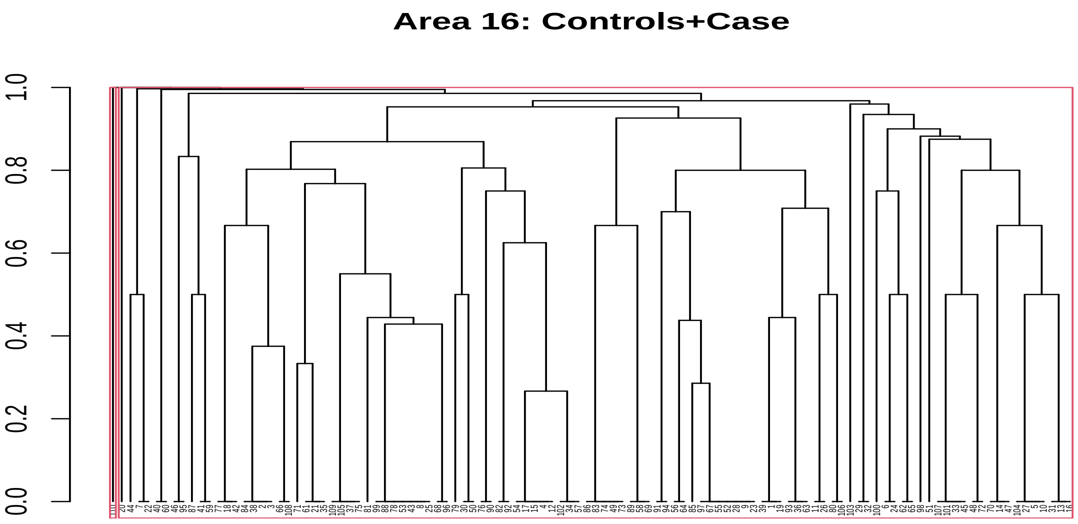

(b) Gamma

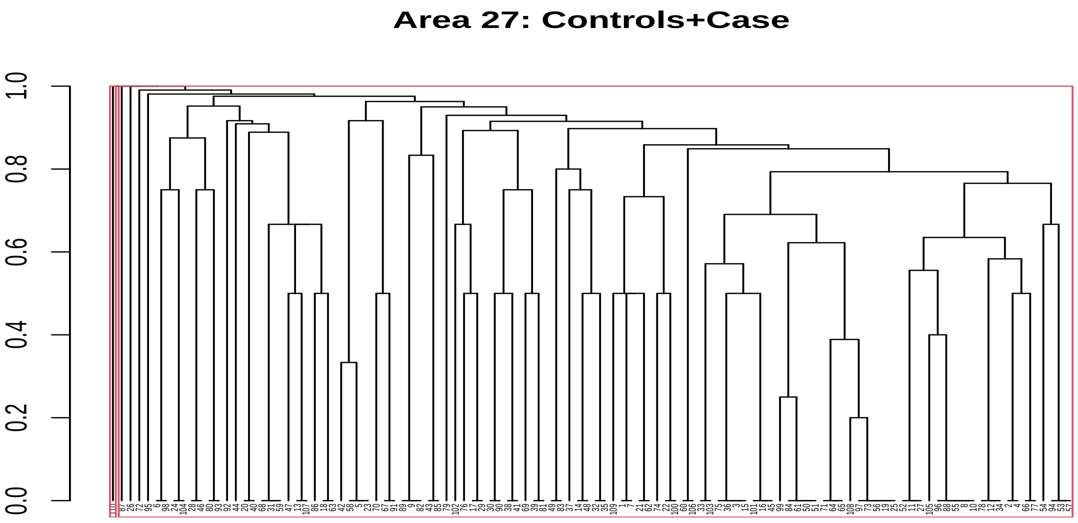

Figure 23: The FLR-HC dendrograms for the areas 16 and 27 in the gamma band of Case 3 respectively. Red boxes indicate the cluster borders if we partition 110 subjects into two clusters.

(a) Gamma

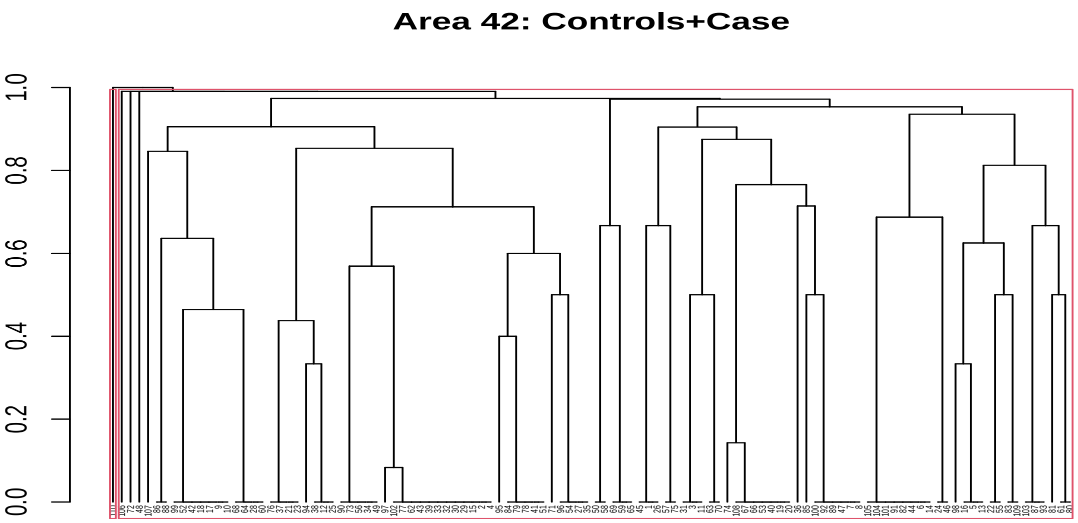

(b) Gamma

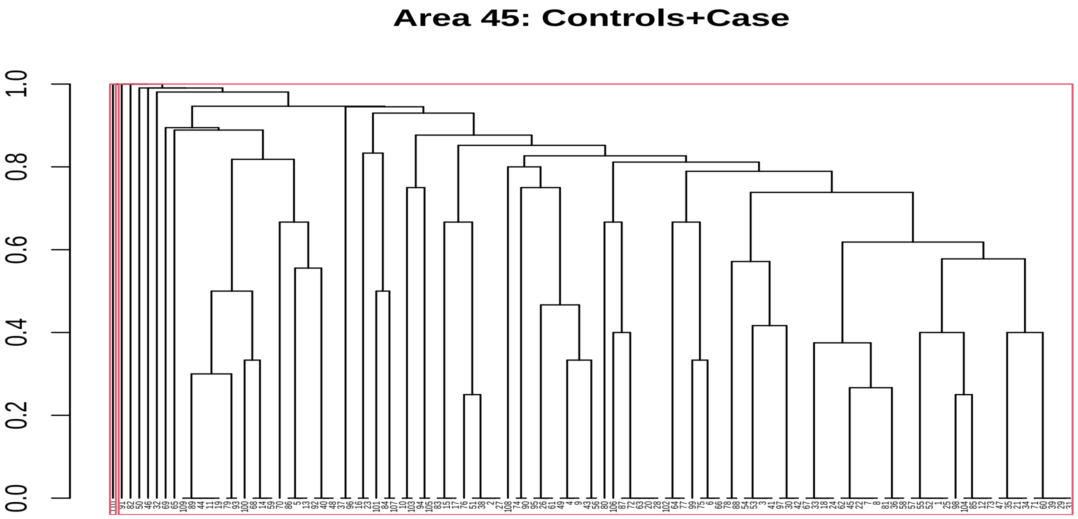

Figure 24: The FLR-HC dendrograms for the areas 42 and 45 in the gamma band of Case 3 respectively. Red boxes indicate the cluster borders if we partition 110 subjects into two clusters.

(a) Gamma

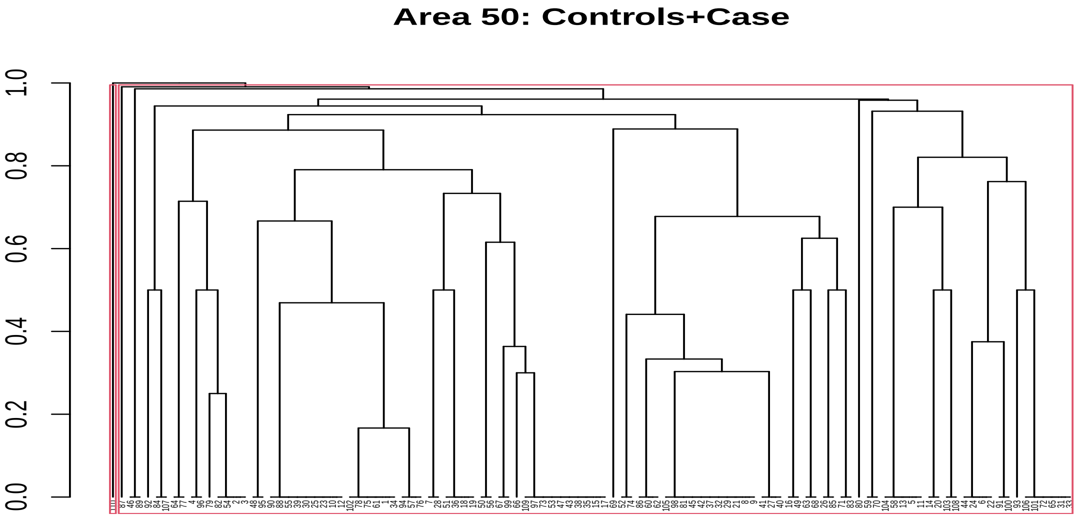

(b) Gamma

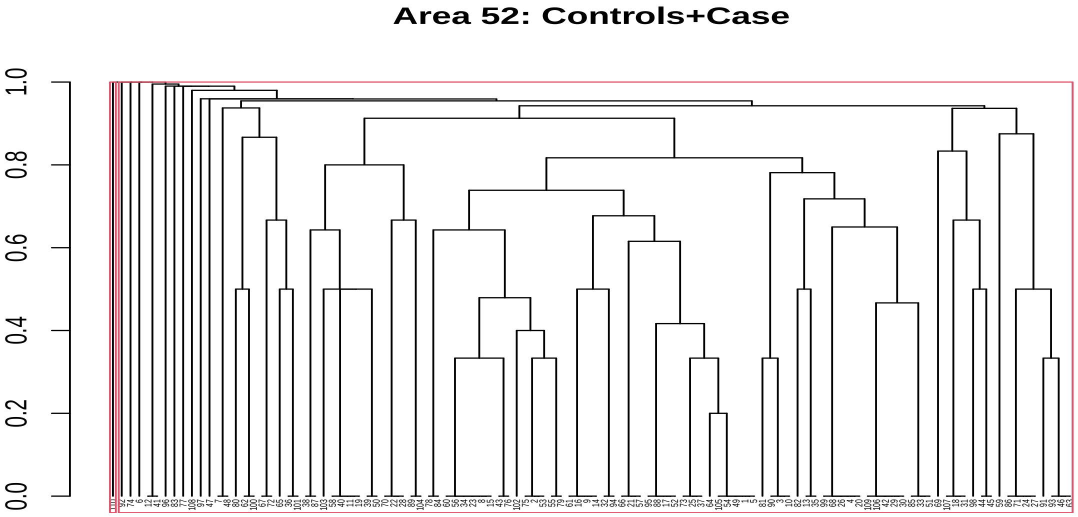

Figure 25: The FLR-HC dendrograms for the areas 50 and 52 in the gamma band of Case 3 respectively. Red boxes indicate the cluster borders if we partition 110 subjects into two clusters.
